# Definition of the Traditional Mexican Diet and Its Role in Health: A Systematic Review

**DOI:** 10.3390/nu11112803

**Published:** 2019-11-17

**Authors:** Selene Valerino-Perea, Laura Lara-Castor, Miranda Elaine Glynis Armstrong, Angeliki Papadaki

**Affiliations:** 1 Centre for Exercise, Nutrition and Health Sciences, School for Policy Studies, University of Bristol, 8 Priory Road, Bristol BS8 1TZ, UK; miranda.armstrong@bristol.ac.uk (M.E.G.A.); angeliki.papadaki@bristol.ac.uk (A.P.); 2 Friedman School of Nutrition Science and Policy at Tufts, Tufts University, 150 Harrison Avenue, Boston, MA 02111, USA; laura.lara_castor@tufts.edu

**Keywords:** Mexican diet, traditional diet, traditional eating, definition, non-communicable diseases, risk factors, systematic review

## Abstract

Promoting traditional diets could potentially reduce the current high rates of non-communicable diseases (NCDs) globally. While the traditional Mexican diet (TMexD) could be specifically promoted in Mexico, a concise definition of the TMexD and evidence of its association with NCDs are needed before its promotion. To evaluate what constitutes this diet pattern, we aimed to systematically review, for the first time, how the TMexD has been described in the literature to date. A secondary aim was to examine whether the TMexD, as described by available definitions, is associated with NCD outcomes. We searched for records describing a whole TMexD up to July 2019 in 12 electronic databases, reference lists, a relevant journal, and by contacting experts on the topic. We reported the results using the Preferred Reporting Items for Systematic reviews and Meta-Analyses (PRISMA) guidelines. We included 61 records for the definition of the diet and six for the association with NCD outcomes. The food groups characterising the TMexD that were consistently mentioned in all the study subgroups were grains and tubers, legumes, and vegetables; specific foods included maize, beans, *chile*, squash, tomato, and onion. Other groups also mentioned, although with lesser frequency, were maize products, fruits, beverages, fish and seafood, meats, sweets and sweeteners, and herbs and condiments. Only a few studies reported on the frequency of consumption or the amounts in which these foods were consumed in the TMexD. It was not possible to reach strong conclusions for the association between adherence to the TMexD and NCD outcomes. The TMexD was weakly associated with developing breast cancer, not associated with triglyceride levels, and inconsistently associated with obesity and diabetes outcomes. However, results were limited by the small number of studies (*n* = 6), of which most were of observational nature and evaluated diets using different TMexD definitions. These findings provide systematically identified evidence of the characteristics of the TMexD. More studies are needed to ascertain the exact quantities by which foods were consumed in the TMexD in order to establish whether this dietary pattern is associated with health and should be promoted within the Mexican population.

## 1. Introduction

Diverse international health organisations have proposed promoting traditional diets to tackle the growing global non-communicable disease (NCD) and obesity rates [1,2]. These diets are generally considered healthy diets [1,3], as they contain large amounts of plant-based foods such as grains, vegetables, legumes, tubers and fruits, and low amounts of foods from animal origin, such as red meat [3,4,5,6]. For instance, the traditional Mediterranean diet has been consistently associated with a reduced risk of developing cardiovascular diseases, cancer, and diabetes [7,8,9,10,11]. As such, adopting a Mediterranean diet is strongly encouraged in the literature [12]. However, promoting the Mediterranean diet might not be feasible in all countries, as different regions might have their own culturally and climate-appropriate food products [13]. 

In Mexico, promoting the traditional Mexican diet (TMexD) could potentially constitute a public health measure to address the country’s high NCD rates [14]. Mortality from NCDs in Mexico has increased by 27% between 1990 and 2017 [14], which has been largely attributed to the nutrition transition the country has experienced [15,16]. However, the literature currently presents diverse definitions of the TMexD, which is potentially due to the changing food culture in Mexico throughout its history [17,18]. These diverse definitions limit the possibility of evaluating the TMexD’s association with health, as different definitions can lead to different outcomes [19]. Determining what constitutes a ‘traditional diet’ is also challenging, as this term can refer to diets consumed in a specific region within a country [20], diets consumed by indigenous populations [2,21], diets present before the industrialisation period [22], or diets conformed by locally produced and culturally appropriate foods [23]. These factors render the definition of the TMexD challenging, and to our knowledge, no consistent definition of the TMexD exists. However, establishing a consistent definition of this traditional dietary pattern and evaluating its association with health outcomes would be invaluable before considering its promotion to reduce NCD rates in Mexico [24]. 

Therefore, the primary aim of this study was to systematically review the evidence, for the first time, of how the TMexD is defined in the literature. Specific objectives were to establish: (1) the specific food groups and individual foods that were consistently characterised as traditionally Mexican from an objective standpoint and by using rigorous and transparent methods [25]; and (2) the amounts consumed of these, if available. The secondary aim of this study was to systematically review the evidence from observational and experimental studies examining the association of the TMexD with NCD incidence or NCD risk factors. 

## 2. Materials and Methods 

This systematic review is reported following a registered protocol (PROSPERO registration number: CRD42018104985) and the PRISMA statement [26] (Supplementary Materials I, Table S1). 

### 2.1. Search Strategy

The search strategy (Supplementary Materials II) was designed by S.V.-P., M.E.G.A., and A.P. after consulting librarians in health sciences and anthropology, and was conducted by S.V.-P. Journal articles, books, and grey literature (i.e., reports and theses) published in English and Spanish were searched up to 9 July 2019. Abstracts and conference proceedings were excluded, as these could lack detailed information on the outcomes of interest. The search was not restricted by year, as current reports can still describe traditional diets, and some current populations might still follow them [27]. 

The databases searched included Anthropology Plus, CENTRAL and Cochrane Reviews, eHRAF World Cultures, Embase, LILACS, MEDLINE, ProQuest (Dissertations and Theses), PsycINFO, Redalyc, SciELO, and Web of Science-Core Collection. The search included a combination of key terms such as ‘Mexico’, ‘dietary pattern’, ‘dietary habits’, ‘traditional’, ‘native’, and ‘regional’ (Supplementary Materials II). We also contacted five experts in the subject, hand-searched the references of eligible documents, and hand-searched a relevant journal (Supplementary Materials II). This journal was hand-searched as it was the only nutrition-related Mexican journal that we identified that did not initially appear on the database search.

### 2.2. Study Eligibility Criteria

#### 2.2.1. Definition of the Diet

For the definition of the TMexD, records describing a dietary pattern and labelling it as traditional Mexican or characteristic of Mexico were included. Records describing the dietary patterns of a specific geographical area of Mexico and those describing the diets of native groups were also included, as these also referred to diets that are distinctive of Mexico. Studies conducted outside of Mexico but referring to a Mexican pattern were also included, as migrants can still follow their countries’ traditional diets [16]. Studies were excluded if they (1) did not focus exclusively on food consumption; (2) did not focus exclusively on Mexican populations (e.g., focused on Latinos or Hispanics); (3) focused on food preparations and popular dishes only; (4) did not describe a whole dietary pattern (e.g., reported single food items or single food groups); (5) were duplicate reports (i.e., different articles by the same authors containing the same information). In the case of duplicate reports, information was extracted from all articles, but the record with the most complete information or the oldest record was considered as the primary source of information. Given that we aimed to identify the foods that were consistently described as traditional Mexican, we also excluded studies that identified more than one dietary pattern consumed in Mexico but did not define any of them as characteristic of the country. For example, we excluded empirical studies comparing prudent/healthy versus unhealthy patterns, or reviews describing all the different dietary patterns consumed throughout Mexican history. We decided to exclude these records, as including them would involve assuming that one or more of the described patterns were traditional Mexican, potentially biasing the results towards our views of the TMexD. 

Both original studies and literature reviews were considered for inclusion in the definition of the diet. All original studies that described the diet as one of the exposures or outcomes and that met the criteria described above were automatically included. As for the literature reviews, only those meeting the criteria and citing multiple references were included. In case one of these ‘multiple references’ was eligible, we also included it. However, in most cases, these references referred to single food groups in the Mexican diet (e.g., plants consumed in Mexico rather than a whole Mexican-style dietary pattern), and thus were not eligible for inclusion. If a review defined the TMexD using a single eligible reference, this single reference was consulted directly and screened for inclusion in the study. In some occasions, some original studies did not describe the traditional diet as part of the outcomes but did describe it as part of the Introduction/Methods section. These studies were treated as literature reviews. That is, such a study was only considered to be eligible if it cited multiple references. Thus, these studies are hereafter also referred to as literature reviews. 

Given that including information from different study designs could provide a more comprehensive definition of the diet [28], we included both quantitative and qualitative records. For example, quantitative studies generally informed the foods that were consumed in large quantities or that provided most of the energy content of the diet. Qualitative studies, on the other hand, emphasised all foods frequently consumed, even if these provided little energy content to the diet (e.g., fruits or vegetables). Lastly, we also included qualitative literature reviews. These reviews were included as they generally used historical or anthropological studies to describe diets, which provided substantial information on the foods that have played a major role in a population’s diet since ancient times. Thus, we included all these sources to allow for a thorough description of the TMexD.

#### 2.2.2. Relationship between the TMexD and Non-Communicable Disease Outcomes

Among all studies meeting the criteria for the TMexD definition (Section 2.2.1), only those evaluating the relationship between a TMexD and NCD health outcomes were retained for further analysis. For this part of our study, both observational and experimental studies were included if they were published in peer-reviewed journals. Only studies focusing on human participants (irrespective of age or health status) and evaluating a TMexD against other types of diets (i.e., modern and/or Western-type diets or a control group) were included. Due to the limited evidence on the topic (found in preliminary searches), any study reporting metabolic risk factors for obesity (body mass index [BMI], waist circumference [WC], fat mass), hypertension (blood pressure), diabetes (glucose, insulin, glycated haemoglobin [HbA1c] concentrations, homeostasis model assessment of insulin resistance [HOMA-IR], and other insulin biomarkers) and dyslipidaemia (blood lipid concentrations) or other health outcomes related to NCDs (e.g., disease incidence) was included. Non-peer-reviewed studies and studies conducted on animals were excluded. These exclusion criteria were not applied for the first objective of the study (definition of the diet) because for that objective we were interested in including a variety of records and study designs to define the diet (i.e. grey literature), in order to provide a more comprehensive definition of the diet.

### 2.3. Study Selection

After eliminating the duplicates, two independent reviewers (S.V.-P. and L.L.-C.) screened the titles and abstracts of the records identified and independently assessed the full texts of the eligible records against the selection criteria. Discrepancies were resolved through discussion (inter-rater reliability: kappa = 0.67; 98% agreement). If no settlement was initially agreed, a third reviewer (M.E.G.A. and/or A.P.) was consulted to reach a consensus. 

### 2.4. Data Extraction

The data extraction form (Supplementary Materials I, Table S2) was piloted independently by two reviewers (S.V.-P. and L.L.-C.) in 10% of the studies. The data in the rest of the studies were also extracted independently by the same two authors (S.V.-P. and L.L.-C.). The data extracted were study characteristics (author, year, country), publication format, study design, period and geographical location corresponding to the diet described, population characteristics, diet assessment method, and dietary pattern description. For the secondary aim (association between the TMexD and NCD outcomes), the comparators (e.g., other types of diets such as modern and/or Western-type diets or a control group), time point of measurements, quantitative outcomes (i.e., odds ratios [OR], mean values), and covariates were also extracted. All disagreements in data extraction were resolved through discussion between the two reviewers. 

### 2.5. Method Used for Categorising the Foods Included in the Definition of the Diet

In order to provide an objective definition of the TMexD, all foods mentioned in the included studies were also extracted into an Excel document (Supplementary Materials I, Table S3). This method was considered appropriate, because both qualitative and quantitative data were included, and because we aimed to establish the foods that are consistently reported to be part of the TMexD. Thus, we calculated the frequency of citation of each food item in all studies. Foods reported using different names were categorised into the same items (e.g., maize and corn; *nopal* and cactus). Unfamiliar items were defined and categorised using the Nahuatl (a native Mexican dialect) dictionary [29] and a Mexican gastronomy dictionary [30]. Unknown animal products were categorised using the biological classification of Mexican species for fish [31,32], mammals and birds [33,34], insects [34,35], amphibians, and reptiles [36]. 

Since many sources cited only food groups (e.g., vegetables) rather than specific items (e.g., squash), all listed items were also categorised into food groups (Supplementary Materials I, Table S3) using Mexican classification food systems [37,38,39]. Thus, in addition to reporting the citation frequency of each item, the frequency of citation of overall food groups was also reported. For example, if one source mentioned “vegetables”, this was counted as one citation for the vegetable food group, and if one source reported “squash” or “squash, tomatoes, and green leaves”, this was also counted as one citation for vegetables. The results are presented for all studies combined as well as separately for literature reviews and original studies due to the different methodologies used by these reports for describing the diets. The results are also presented separately for the different geographical areas in Mexico [40] to illustrate the potential regional differences in the country (not pre-specified in the review protocol). Records referring to more than one geographical region were considered in all relevant analyses (e.g., those referring to both Central and Southern Mexico were included in the Central Mexico analyses and in the Southern Mexico analyses); records referring to non-Mexican regions (i.e., the United States) were excluded from these analyses. For each analysis, we reported the food groups cited in at least 50% and 75% of the studies and the individual foods mentioned in at least 25% of the studies. 

### 2.6. Data Analysis for the Association between the Traditional Mexican Diet and Health

Most of the studies evaluating the association between the TMexD and NCDs used different outcomes or assessed the diet using different methods. For this reason, the association between the TMexD and health outcomes is presented as a narrative synthesis rather than a meta-analysis.

### 2.7. Study Quality, Risk of Bias, and Quality of Reporting

For the studies reporting the definition of the TMexD, study quality was assessed by adapting an index used by Green et al. [41] in a systematic review that was used to establish a description of dietary patterns in India. This index evaluated whether studies described: (1) a population located in Mexico or of Mexican ancestry; (2) the foods included in the diet; (3) the proportions of the foods included; (4) the methodology followed to derive the dietary pattern; (5) the population represented; (6) the years/period represented; and (7) the geographical location of the diet described.

For the studies evaluating the association with NCD outcomes, we also evaluated the risk of bias and the quality of reporting. We evaluated the risk of bias using the Newcastle Ottawa Scale for case-control, cross-sectional, and cohort studies [42,43], and the Cochrane risk of bias tool for randomised controlled trials [44]. We evaluated the quality of reporting using the STROBE statement for case-control, cross-sectional, and cohort studies [45], and the CONSORT statement for randomised controlled trials [46]. These domains were assessed independently by two reviewers (S.V.-P. and L.L.-C.); all discrepancies were resolved through discussion. 

## 3. Results

**Figure 1:**
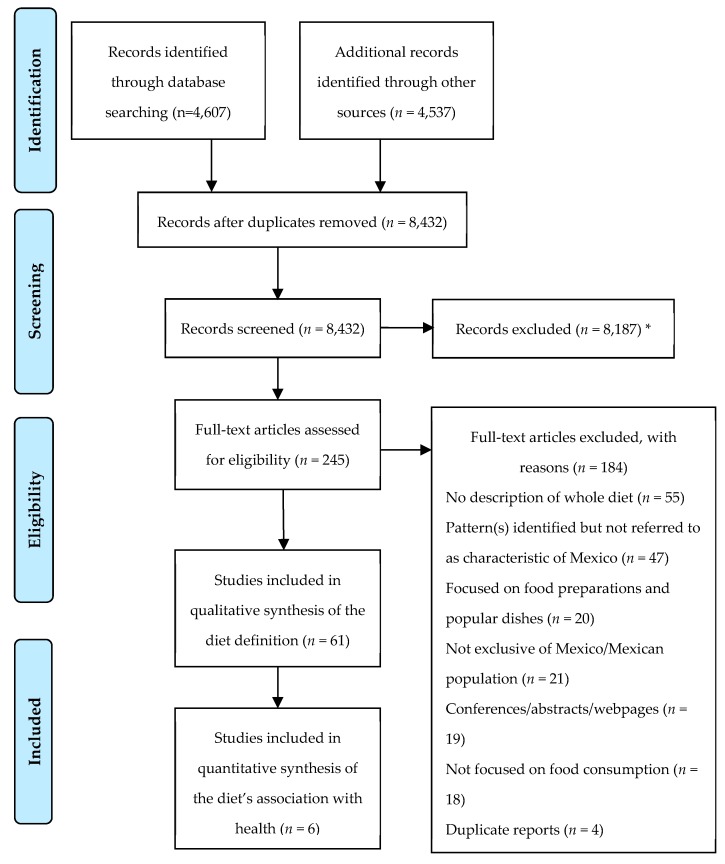
Preferred Reporting Items for Systematic reviews and Meta-Analyses (PRISMA) flow diagram of literature search and study selection. * Seven of these records could not be retrieved despite several attempts to contact the corresponding authors.

After removing the duplicates, 8432 records were identified, of which 8187 were removed after screening titles and abstracts. Most of the records eliminated at this stage referred to records not related to diet or to records describing the intake of one single food (e.g., maize consumption in Mexico). A total of 245 records were retained for full-text examination, and 184 were later removed (Figure 1; Supplementary Materials 1, Table S4). As such, 61 records were included for the establishment of the definition of the TMexD, and six were included for the evaluation of its association with NCD health outcomes. 

### 3.1. Findings on the Definition of the Traditional Mexican Diet

#### 3.1.1. Study Characteristics

Among the 61 included records, 43 were literature reviews [47,48,49,50,51,52,53,54,55,56,57,58,59,60,61,62,63,64,65,66,67,68,69,70,71,72,73,74,75,76,77,78,79,80,81,82,83,84,85,86,87,88,89], and 18 were original studies [90,91,92,93,94,95,96,97,98,99,100,101,102,103,104,105,106,107] (Table 1). Among the literature reviews, most (*n* = 26) used historical, archaeological, or ethnographic data to describe the TMexD. Historical data mostly consisted of Spanish manuscripts describing indigenous food habits before the Mexican colonisation. Archaeological data consisted of the remains of foods and cooking instruments, whereas ethnographic data consisted of direct observations of diets of indigenous populations. Among the original studies, 14 used quantitative methods to assess the TMexD, and four used qualitative methods to derive the diet. Most studies referred to diets consumed in Central Mexico (*n* = 24; three original studies and 21 literature reviews) (according to the National Health and Nutrition Survey geographical areas) [40], diets of indigenous populations (*n* = 40; nine original studies and 31 literature reviews), and diets present before the colonisation of Mexico (*n* = 30; 30 literature reviews) (Table 1). 

#### 3.1.2. Definition of the Traditional Mexican Diet in All Studies

The individual food items present in at least 50% of the studies were maize, maize tortillas, beans, squash, tomato, *chile*, and chocolate drinks (Table 2; Supplementary materials, Table S5). When the items were grouped into food groups, the most cited (75% of the studies) groups were grains and tubers, maize products, legumes, vegetables, fruits, meats, and herbs and condiments. Among these groups, the most cited items are presented in Table 2a. At least 50% of the studies also mentioned oils and fats, nuts and seeds, beverages, fish and seafood, and sweets and sweeteners. Among these groups, the most cited items are presented in Table 2b.

#### 3.1.3. Definition of the Traditional Mexican Diet in Literature Reviews Versus Original Studies

Due to the different methodologies used for describing diets in literature reviews and original studies, the results are also presented separately for these sources. Compared to the results in all studies combined, literature reviews additionally mentioned insects and reptiles to the food groups mentioned in at least 50% of the studies (Table 3a,b). Conversely, original studies did not mention nuts and seeds, insects, and reptiles as part of the TMexD (Table 4a,b), but added dairy and eggs to the food groups present in the diet. Another difference in original studies was that maize products were only mentioned in 50% of the original studies compared to 75% of literature reviews.

Some differences were also observed in the individual foods mentioned in literature reviews compared to original studies. Overall, the literature reviews included more comprehensive lists of food items than the original studies. Conversely, the original studies mentioned few individual items, most of which were also mentioned in the literature reviews, except for rice, bananas, vegetable oils, coffee, tea, soda, and sugar. 

#### 3.1.4. Definition of the Traditional Mexican Diet According to Different Geographical Regions

When the diet definition was assessed separately for the different country regions, some differences were also observed. Records referring exclusively to Northern Mexico (*n* = 7; six original studies and one literature review) reported a similar diet to the original studies. For example, these studies mentioned a low number of food items, and included eggs and dairy (in 50% of studies) to the TMexD’s description, and maize products in only 50% of the studies (Table 5). On the other hand, beer was mentioned in the North Mexican diet, which was not mentioned in other descriptions. 

The records referring exclusively to Central Mexico (*n* = 24; three original studies and 21 literature reviews) showed a diet similar to the literature reviews’ description. That is, most of the food groups and individual foods were mentioned in the same manner as in the literature reviews. However, fish and seafood were mentioned more frequently than in other studies, whereas fruits were mentioned less frequently. Central Mexican diets also included additional vegetables (*huauzontle*), meats (squirrel), and insects (*amoyotl*, *ahuahutle*, and other worms), which were not mentioned in other definitions (Table 6). 

The records referring exclusively to Southern Mexico (*n* = 11; two original studies and nine literature reviews) showed some differences compared to other descriptions. Nuts and seeds and insects were mentioned more frequently (75% of studies) compared to other geographical regions. Additionally, compared to other descriptions, the Southern Mexican diets also included additional maize products (*pinole*), vegetables (*guaje*, *papaloquelite, quintoniles*), fruits (*nanche*, *ramón*), lard and animal fats, meats (partridges), herbs and condiments (*acedera*, *chipilín*), and fish (catfish), which were not mentioned in other definitions (Table 7). 

Lastly, out of the 61 records, we also examined separately those referring specifically to all regions in Mexico (*n* = 14; three original studies and 11 literature reviews). These studies had the most extensive list of food groups. However, they did not include reptiles, which were mentioned in some literature reviews and regional diets. Studies referring to all Mexican regions also reported individual foods not mentioned in other definitions. These included more maize products (soups and other products), vegetables (carrot, lettuce, *huitlacoche*, and squash blossoms), fruits (apple, berries, mango, melon, pear, peach, and *pitahaya*), meats (lamb and chevon), herbs and condiments (*achiote*, coriander, garlic, parsley, and pepper), seeds (sesame seeds), oils and fats (cream), beverages (*tesgüino*, *aguas frescas,* and natural fruit juice), eggs (chicken eggs), and sweets and sweeteners (*pan dulce*, desserts, and sweets) (Table 8).

#### 3.1.5. Amounts of Foods Consumed in the Traditional Mexican Diet

Eight records that included original studies reported on the amount that specific foods were consumed in the TMexD [91,93,95,96,99,101,104,107]. Four assessed the percentage of contribution to the total energy intake (TEI) using a posteriori methods [93,95,96,99], three evaluated the quantities of foods consumed per week or per day [91,104,107], and one assessed the frequency of consumption of different foods [101]. One study also suggested a diet score to assess adherence to the TMexD [78]. However, this study was not included in this section, as the authors did not establish specific amounts for the different foods included. Instead, the score measured adherence to the TMexD based on participants’ median intakes [78]. A second study was also excluded from this analysis, as it did not specify quantitative recommendations for some of the food groups (e.g., vegetables) [50]. Lastly, one qualitative study was excluded as it mentioned the frequency of consumption of some, but not all, foods described [98]. 

It was not possible to reach a consensus on the amounts of the different food groups included in the TMexD, as all eight studies reported different amounts. For instance, even the products that were mentioned in high amounts, such as maize, differed widely within studies using the same method (Supplementary Materials I, Table S6). The TEI from maize products varied from 35% to 47% between studies using this method [84,86,87,90], while the grams of maize consumed per week varied from 847 to 4188 [92,95,98]. This wide variation also applied for other groups such as legumes (i.e., beans), red meat, dairy, fruits, and vegetables. Likewise, some studies grouped all foods differently. For example, some studies combined all grains together [90], while others separated maize, wheat products, and tubers [84,86,87]. Additionally, some food amounts were non-comparable between studies, as they were reported only in some studies. For example, some food groups, such as processed foods (e.g., breakfast cereals, cakes, sweets, and fast foods), eggs, alcohol, and other beverages were absent in some studies (Supplementary Materials I, Table S6). These differences limited the ability to reach a consensus on the amounts of foods and food groups included in the TMexD.

#### 3.1.6. Study Quality Assessment

**Figure 2:**
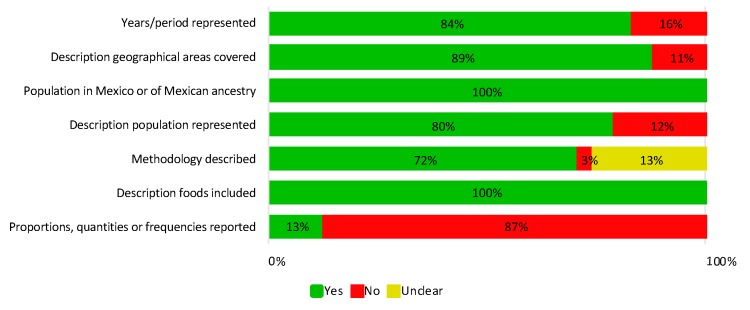
Proportion of included articles reporting the study quality index components.

Most indicators of the study quality index were reported in most studies (Supplementary Materials, Table S7). The only items with limited description were the quantities of the foods or foods groups included in the diet, and the description of the methodology used to derive the diet (Figure 2). 

### 3.2. Findings on the Association of the Traditional Mexican Diet and Health

#### 3.2.1. Study Characteristics

Among all 61 studies included to define the diet, six of them also assessed the relationship between the TMexD and NCD health outcomes [78,79,93,95,100,103]. Three were cross-sectional studies [93,95,103], one was a case-control study [100], one was a cohort study [78] and one was a randomised cross-over feeding trial (RCT) [79]. Most (*n* = 4) studies derived the diet using a posteriori methods [93,95,100,103]; one used a ‘traditional Mexican diet’ score using median intakes [78], and the RCT assigned a seven-day menu to its participants using the ‘traditional Mexican diet’ score as reference (Table 9). Four were conducted in the United States (US) [78,79,93,100] and two were conducted in Mexico [95,103]. Three studies reported differences in diabetes-related outcomes [78,79,103], while two studies reported differences in obesity [93,95], one studies reported differences in breast cancer [100], and one studies reported differences in dyslipidaemia [78] (Table 9).

#### 3.2.2. Breast Cancer

One case-control study reported a 32% reduced risk for breast cancer development in women with high adherence to the TMexD, compared to those with the lowest adherence (odds ratio (OR): 0.68; 95% confidence interval (CI): 0.55 to 0.85; *p* < 0.01) [100] (Table 9). 

#### 3.2.3. Diabetes-Related Outcomes

Three studies showed reduced odds of some diabetes-related outcomes (Table 9). One cross-sectional study reported a 51% reduced risk of having pre-diabetes (OR: 0.49; 95% CI: 0.31 to 0.79; *p*: 0.003) in adults following a traditional diet [103]. Similarly, a cohort study showed 15% lower insulin values in women with high adherence to the TMexD (mean: 12.2 ± 10.7 μIU/mL), compared to those with lower adherence (mean: 14.0 ± 11.1 μIU/mL, *p* < 0.05) [78]. Likewise, after following the TMexD for 24 days, an RCT also showed reductions in insulin levels by 14% (mean difference: 1.26 μU/mL, *p*: 0.02), in insulin-like growth factor binding protein-3 (IGFBP-3) by 6% (mean difference: 121 ng/mL, *p*<0.01), and in insulin-like growth factor-1 (IGF-1) by 4% (mean difference: 5.72 ng/mL, *p*: 0.06) in women of Mexican descent. [79]. 

However, no differences were observed in glucose values or in the IGF-1:IGFBP-3 ratio (Table 9). Blood glucose values were similar in women with high (mean 98.7 ± 20.5 mg/dL) and low (mean: 97.3 ± 17.3 mg/dL; *p* > 0.05) TMexD adherence in the cohort study [78]. Similarly, in the RCT, both the glucose values and the IGF-1:IGFBP-3 ratio were similar when women followed a TMexD, compared to when they followed a US diet (mean difference for glucose values: 0.82 mg/dL, *p*: 0.42; mean difference for IGF-1:IGFBP-3 ratio: −0.09, *p*: 0.38) [79].

Finally, findings for HOMA-IR outcomes were inconsistent (Table 9). The cohort study showed similar values in women with high (mean: 3.13 ± 3.31) and low (3.49 ± 3.36; *p* > 0.05) adherence to the TMexD score [78], whereas the RCT did show a 15% reduction in HOMA-IR values (mean difference: 0.30; *p*: 0.02), when women followed a TMexD [79]. 

#### 3.2.4. Obesity Outcomes

One cross-sectional study reported no differences in BMI (mean: 28.3 ± 0.5; *p* > 0.05) or WC values (mean women: 93.1 ± 1.7, *p* > 0.05; mean men: 97.8 ± 1.6, *p* > 0.05) in Mexican American adults following a TMexD, compared to those following other diets [93] (Table 9). However, another cross-sectional study did report an increased risk for being overweight or obese in Mexican adults following other diets when compared to a traditional one (Table 9). A refined diet (characterised by high amounts of alcohol, soft drinks, bread, and fast foods) was related to a 14% (OR: 1.14; 95% CI: 1.02 to 1.26; *p*: 0.01) and a 20% (OR: 1.20; 95% CI: 1.09 to 1.31; *p*: 0.001) higher risk for being overweight and obese, respectively [95]. The diverse diet (characterised by high amounts of whole-fat dairy, rice and pasta, meats, and eggs) was related to a 17% (OR: 1.17; 95% CI: 1.03 to 1.33; *p*: 0.01) and a 20% (OR: 1.20; 95% CI: 1.08 to 1.34; *p*: 0.001) increased risk for being overweight and obese, respectively [95]. 

#### 3.2.5. Lipid Outcomes

One cohort study reported no differences in triglyceride levels in women with high adherence to the TMexD (mean: 123 ± 58.4 mg/dL), compared to those with the lowest adherence (mean: 125 ± 49.3 mg/dL; *p* < 0.05) [78] (Table 9). 

#### 3.2.6. Risk of Bias and Quality of Reporting Assessment

There were heterogeneous results in the items with low, unclear, or high risk of bias, depending on the study design (Supplementary Materials I, Figure S1). Overall, the case-control study showed a potential risk of bias in the selection of controls, the ascertainment of the exposure, and the similarity in response rates for cases and controls. The cross-sectional studies showed a potential high or unclear risk of bias in the comparability between respondents and non-respondents and in controlling for confounding factors. The cohort study showed an unclear risk of bias for the ascertainment of the exposure, whereas the RCT showed an unclear risk of bias in the random sequence allocation and allocation concealment. 

As for the quality of reporting, none of the studies reported all items in the STROBE or the CONSORT statement checklist (Supplementary Materials I, Tables S8–9). In the STROBE statement, the items most underreported were the identification of study design in the title, study size, sensitivity analyses, reporting of flow diagrams, reporting of missing data, and translations of relative risks to absolute risk (Supplementary Materials I, Figure S2). In the CONSORT statement, the items most underreported were changes to methods, changes to outcomes, sequence generation, type of randomisation, allocation concealment mechanism, randomisation implementation, and harms and unintended effects (Supplementary Materials I, Table S9). 

## 4. Discussion

### 4.1. Definition of the Traditional Mexican Diet

The present study defined the TMexD by listing the food groups and food items most often referred to as traditional Mexican in the literature. The analysis was conducted for all included studies but also separately, according to the type of study and region of Mexico. Since most (71%) of the literature reviews referred to diets before or around the Spanish colonisation (i.e., the 16th century) and all original studies reported more current intakes (i.e., 1943 onwards), this separate analysis allowed the examination of variations in the TMexD definition through time. In addition, the subgroup analysis according to different geographical areas of Mexico (i.e., North, Central, South, and all regions) allowed the examination of potential marked differences across regions.

#### 4.1.1. Foods Present Consistently in the Traditional Mexican Diet

The results suggest that grains, legumes, and vegetables were the most representative food groups in the TMexD, as these were mentioned in most (75%) of the studies, including all the subgroups evaluated (Supplementary Materials I, Table S10). Additionally, maize (mostly as tortillas), beans, squash, tomato, *chile*, and onion are potentially fundamental elements of the TMexD, as these were the only individual foods cited in most studies, including all the subgroups evaluated (Supplementary Materials I, Table S11). Indeed, maize (a grain), beans (a legume), and squash (a vegetable) have long represented the basic foods in Mexico, as they form part of the ancient agro-ecosystem known as *milpa* [108]. *Chile*, while also present only in some *milpa* cultivations [108,109], is specifically characteristic of the Mexican diet and its cuisine [110], which might also explain its high citation. Often, *chile* is also combined with tomato and onion in sauces to condiment meals [56]. 

Other groups that were also mentioned in all the studies and subgroup analyses were maize products, fruits, beverages, fish and seafood, meats, sweets and sweeteners, and herbs and condiments (Supplementary Materials, Table S10). However, these were mentioned in different frequencies in the different subgroups assessed (i.e., in ≥75% of studies in some subgroups while in only 50% of studies in others). Specifically, maize products, beverages, meats, and herbs and condiments were mentioned in 75% of the literature reviews, but in only in 50% of the original studies. Likewise, studies referring to Northern and Southern Mexico mentioned meats, herbs and condiments, and maize products less frequently than other regions, respectively. On the other hand, studies referring to Central Mexico mentioned fruit less frequently but fish and seafood and beverages more frequently. Lastly, records referring to all the regions mentioned sweets and sweeteners more frequently than the rest of the specific geographical locations. 

Some possible explanations for these differences might exist. For instance, literature reviews could have described a more detailed diet, thus emphasising the ways in which maize was consumed, and the herbs and condiments used for cooking. A second explanation is that a posteriori analyses, which were used in 44% of the original studies, might have emphasised only the foods that have the largest contribution to the TEI [111]. Thus, they might have not reported foods that are characteristic of the TMexD but are generally non-energy dense, such as herbs and condiments [37]. While some Mexican condiments do provide large amounts of calories, mainly from fat (i.e., *adobo*, *mole*, *pipián*), all of the herbs and condiments identified in this review were non-energy dense [37,38,39], and thus these potentially do not contribute largely to the TEI. However, these reasons do not explain why meats and beverages were reported less frequently in original studies. As for the regional differences in the TMexD, the results indicate that beverages, fish and seafood, meats, fruits, and maize products are consumed differently throughout the country. However, these regional differences might have only been representative of past times, as current intakes of these food groups disagree with the results observed. For example, although meats were less reported in the north, the meat intake has long been higher in this region of Mexico [112]. Likewise, although fruits were less reported in Central Mexico, the fruit intake has been higher in this region both in the past [48] and present times [113,114]. Thus, given that the different study designs or the regional variations might not explain these differences in frequency of citation, these food groups might simply not have a primary presence in the TMexD. Therefore, while these food groups are highly likely to form part of the TMexD, they might be less representative of the diet than grains, tubers, legumes, and vegetables.

#### 4.1.2. Foods Not Consistently Mentioned in the Traditional Mexican Diet

Some food groups (oils and fats, nuts and seeds, eggs, dairy, insects, and reptiles) were mentioned only in some, but not all, the subgroups of the studies (Supplementary Materials I, Table S10). Specifically, nuts and seeds, insects, and reptiles were present in literature reviews but were not reported by original studies, which might imply that they have become less representative of the TMexD over time. Indeed, the current consumption of nuts and seeds in Mexico is low [114,115], and insects and reptiles no longer form part of the usual diet, as these are only consumed as exotic foods [116] or in some southern or rural areas in Mexico [110,117,118,119]. In contrast, dairy and eggs were only mentioned in original studies, but not in literature reviews, which might imply that these foods were only recent inclusions to the Mexican diet. As for the regional subgroup differences, only studies referring to the north mentioned eggs and dairy, while only studies referring to Central and Southern Mexico included oils and fats, nuts and seeds, insects, and reptiles. It is possible that these food groups were consumed to different extents in the different country regions in the past. However, some of these differences might not have persisted, as current intakes of oils and fats, and nuts and seeds, are similar in all regions of the country, and current intakes of dairy are higher in the center, rather than the north of Mexico [113,114]. 

Including these food groups that were not consistently mentioned in a definition of the TMexD might require careful consideration. Oils and fats, and nuts and seeds might still form part of the TMexD, although these might be less representative of the diet than the other groups previously mentioned. Oils and fats, and nuts and seeds are not only consumed in present times, but they were also mentioned both in all studies combined and in studies describing all geographical regions in Mexico. On the other hand, eggs and dairy might not be as representative of the TMexD as other foods that have long formed part of the Mexican food tradition. Insect consumption, while still well accepted at the population level, might only occur occasionally [116]. However, reptile consumption may no longer be as relevant in Mexico, especially if this diet was to be promoted in the general population. Reptiles were absent in all studies combined and those describing all geographical regions, and their commercialisation (i.e., turtles, iguanas, and snakes) is prohibited in order to preserve the species [117,120,121].

#### 4.1.3. Specific Food Items Mentioned in the Traditional Mexican Diet

There was a large variation in the individual foods mentioned according to the subgroup analyses. While all the food items mentioned in the results might be considered when defining the TMexD, special consideration might be needed for the items that, based on their absence in food classification systems, are no longer common in Mexico. This might be true for some meats (i.e., dogs, boar, *tlacuache* [opossum], squirrel, partridges, and gopher), vegetables (i.e., *Spirulina* algae, maguey, *mezquite*, *quintoniles*, and *Setaria*), fruits (i.e., *ramón*), and herbs (i.e., *acedera*). Conversely, since processed or industrialised products might represent less traditional items, foods such as vegetable oils, soda, *pan dulce* (sweet bread), desserts, and sweets might only be present in limited amounts. Future studies should examine the feasibility of integrating these unusual or processed foods in a traditional diet, especially if the purpose is to promote it as a public health strategy to reduce NCD rates. 

#### 4.1.4. Amounts of Food Groups Included in the Traditional Mexican Diet

There was high heterogeneity regarding the quantities of the foods in the diet reported in the literature. While many studies reported maize (*n* = 38), beans (*n* = 32), *chile* (*n* = 24), and squash (*n* = 20) as basic items of the diet and animal foods (*n* = 9) as limited items, establishing specific amounts of these items is essential when defining a dietary pattern, especially if this pattern is to be studied in relation to health [19]. This heterogeneity could have been the result of the inclusion in this review of different studies, which used different methods to describe the TMexD. Some authors reported amounts of food groups using percentages of contribution to the TEI, while others used frequencies and quantities consumed per week, all of which resulted in non-comparable outcomes. For example, when foods are assessed using percentages of contribution to the TEI, the results often depend on the energy density of the food evaluated [122,123]. That is, foods with lower energy densities such as fruits, vegetables, and herbs contribute less to the diet, even if they are consumed frequently or in large quantities. Further heterogeneity can be introduced when dietary patterns are derived using a posteriori analyses, as researchers might group the foods included differently [3] (e.g., grouping all maize products into one category or grouping them separately). As such, defining the amounts of foods that characterise the TMexD requires further examination. Future studies should refine the current diet definition results in order to establish a more concise and quantitative description of the TMexD, which might need to be specific to the target population (e.g., adults). The amounts of the food groups included in the TMexD might be more appropriately defined using frequencies and quantities, in order to avoid basing the food amounts on the energy density that these provide and allow easier interpretation by the public.

### 4.2. Association between the Traditional Mexican Diet and Non-Communicable Disease Outcomes

This study also summarised the current evidence relating the TMexD with NCD health outcomes. Given that most (83%) of the studies evaluated in this review were observational and given the possible risk of bias in some of the domains evaluated, it was not possible to reach strong conclusions on the health outcomes associated with adherence to the TMexD. Based on this evidence, the TMexD was inversely but weakly associated with breast cancer risk [100] but not related to blood triglyceride levels [78]. The associations with obesity and diabetes-related outcomes were inconsistent. In cross-sectional studies, the TMexD was associated with a reduced risk for being obese [95] but not with differences in BMI and WC [93]. Similarly, for the diabetes outcomes, high adherence to the TMexD was associated with lower insulin concentrations [78,79] and with a lower risk or having pre-diabetes in observational studies [103], but not with glucose levels (in either the cohort study or the RCT) [78,79]. Likewise, the changes in HOMA-IR and insulin biomarkers (i.e., IGF-1, IGFBP-3, IGF-1:IGFBP-3) in adults following a TMexD were inconsistent in the RCT [79] and in the cohort study [78]. However, these insulin biomarkers might only provide information about insulin resistance in obese individuals [124]; thus, they might not provide reliable information on diabetes outcomes. As such, all these results must be interpreted with caution, especially since only a small number of studies measured the same outcomes, and most used a different definition of the TMexD, limiting the ability to compare results [19].

However, the TMexD, as identified in the present study, could potentially be considered a healthy dietary pattern. While the amounts of the foods included in the TMexD were inconclusive, which is essential to characterise a dietary pattern as healthy or unhealthy, the identified TMexD possesses some similarities with public health guidelines [125]. The TMexD is potentially high in fibre, as it contains grains, legumes, and fruits and vegetables. High fibre intakes have been consistently inversely associated with some obesity- and diabetes-related outcomes (i.e., BMI, body fat, fasting glucose, and fasting insulin) [126], colon cancer [127,128], and cardiovascular disease [129]. The TMexD is also potentially high in antioxidants, provided by fruits, vegetables, and some legumes and seeds [51,125], which have been associated with a reduced risk of developing cardiovascular diseases and cancer [130]. While the TMexD might also contain meats and animal products, sugars, and caloric beverages, which have been discouraged in the literature, these can still be part of a healthy diet, especially if these are consumed in small quantities [131]. However, more research is needed to evaluate the proportions in which these foods are present in the TMexD before producing high-quality evidence of the TMexD’s association with health outcomes. Future studies could also evaluate the importance of traditional Mexican food preparation methods on NCD-related outcomes, as different preparations (i.e., different ingredients and cooking methods) can lead to different content in fibre, water, fat, and sugar, all of which could be relevant to foods’ energy density [132,133]. It could also be important to identify the traditional dishes that contain an appropriate nutrient composition. Then, food preparations that are most in line with dietary recommendations and that promote health the most might be recommended over other preparations. 

Future studies should use the current TMexD definition results and define the proportions in which these foods are present in the Mexican diet, preferably in the form of a dietary index. Then, an authentic Mexican diet, rather than a subjectively defined diet (i.e., using a posteriori analyses), could be examined with regard to its role in health outcomes. As previously discussed, although some TMexD indices [78] and quantitative recommendations [50] already exist, these might be complemented with some key items in the Mexican diet identified in the present study. For example, given the high presence of herbs and condiments in the TMexD, these might need to be evaluated separately, as they not only provide antioxidants to the diet [22], but they also improve the palatability of different foods and meals [134,135]. Likewise, more research is needed to establish whether beverages and more ‘unhealthy’ items, such as processed foods, could be included in limited amounts in the definition of the TMexD, particularly as these have been recently included in the Mexican diet. 

### 4.3. Strengths and Limitations 

The major strength of this study was the collation of extensive literature to define the TMexD, which was inclusive of different time periods and geographical areas. Other traditional diets have been defined by interviewing older individuals during 1993–1994 (i.e., the Mediterranean diet) [136], identifying regional and traditional products still consumed in present time (i.e., the Japanese diet) [137], or identifying the current locally grown products with potential health properties (i.e., the Nordic diet) [13,138,139]. However, since the food culture in Mexico has changed significantly over time [18], these methods alone were not considered appropriate to establish a definition of the TMexD. Instead, we described the TMexD as reported in the literature and identified the foods that were consistently characterised as traditional Mexican by studies with a variety of designs. Rather than describing the TMexD from our personal views, this method allowed us to report a more comprehensive and less biased record of the foods included in this traditional diet, while adhering to a rigorous and transparent systematic methodology, and following the PRISMA guidelines [25]. To our knowledge, this is the first attempt to define the TMexD using a systematic review of the literature. 

A second strength of the present study is the consideration of how the TMexD might differ according to study design (literature reviews and original studies), and the different geographical regions. As such, we could report the potential variations that the TMexD has experienced through time and the regional variations that exist in the country. We could also report the food groups and food items that are potentially the most representative of the diet (i.e., those reported in all subgroups) and those that are less representative (e.g., in different regions of the country). A final strength is the evaluation of the TMexD’s association with health using established guidelines for assessing the risk of bias and quality of reporting in these studies. 

The present study also has some limitations. Firstly, most of the included studies described diets consumed in Central Mexico (40%) or by indigenous populations (65%). As such, these areas of Mexico and these populations might be over-represented in the definition of the TMexD. However, we did include sources describing different regions and populations in Mexico, and we stratified the results by geographical location to observe whether some differences within them were evident. Nevertheless, these results might imply that the TMexD described in this study might be more appropriate for these populations and that interventions to potentially promote the TMexD, as described in this study, might be more feasible in these contexts. Indeed, the original studies that evaluated all regions in Mexico found a higher adherence to a traditional-like diet in people with indigenous backgrounds [95,96]. However, interestingly, some authors also found higher adherence in southern areas of Mexico [96], rather than central ones.

Secondly, half of the records referred to precolonial diets; thus, some of the food habits reported in this review might no longer be representative of, or acceptable, in modern Mexico. To overcome this limitation, we also analysed literature reviews and original studies separately. This subgroup analysis potentially highlighted the food items that may no longer be traditional Mexican, which is especially relevant since traditional foods must also be accepted by the population and characterised as desirable [23]. Likewise, most of these precolonial records relied on historical sources to describe the diet (i.e., Spanish manuscripts describing the food ways of the indigenous Mexicans). The reliability of these sources might be questioned given the time in which these were collected (i.e., 16th century) and the potential lack of valid protocols to collect these data. However, historical sources provided information on important food habits before the Spanish colonisation of Mexico. More importantly, the information included in historical sources was largely compatible with that reported in archaeological sources, and these often were used in conjunction to describe the TMexD in this review. 

Thirdly, the results might also have been influenced by the methods used to analyse the data. That is, the classification of some foods or food groups might have influenced the results. For example, when analysed separately, tubers were mentioned less than grains, and red meats were mentioned less than poultry (Supplementary Materials I, Table S3). To overcome this limitation and achieve an objective classification of the foods included, we grouped them using the Mexican food classification systems only [37,38,39]. In a similar manner, the results of the subgroup analysis by geographical region might have been biased by the type of methodology used. Specifically, studies describing the north area of Mexico were mostly (86%) original studies, while most (83%) of the studies describing the centre and south of Mexico were literature reviews. However, we still observed some differences in these subgroups. For example, oils and fats were mentioned in original studies but not in studies referring to the north of Mexico, and some groups were mentioned differently in Central and Southern Mexico despite both being based mainly on literature reviews.

The assessment of the relationship between the TMexD and health also presents limitations. Most studies were subject to some type of bias depending on the study design, which could have overestimated the associations observed. For example, the RCT did not report random sequence generation and allocation concealment appropriately, whereas cross-sectional studies did not describe non-respondents or control for all potential confounders. Accounting for these factors can help reduce selection bias and help ensure that no other factors are responsible for the differences observed between the groups compared [140,141,142,143]. An appropriate ascertainment of the diet consumed was also missing in the case-control and cohort studies. Lastly, the case-control study did not report the same response rates for cases and controls, which would ensure comparability between these groups [140]. Likewise, most results were based on observational, rather than interventional studies. Observational studies are more prone to recall bias and residual confounding, and are not as able to demonstrate temporality [144]. Nevertheless, the intervention study had a short-term duration, which might have also prevented the observation of positive changes to some NCD health outcomes. 

Lastly, the studies included in this review are limited by the definitions of the TMexD used. The case-control and the cross-sectional studies used a posteriori methods to evaluate the diet, which relied on what the current population consumes [3]. Since traditional diets also involve past dietary habits [22], it is questionable whether these studies evaluated an authentic traditional diet. Indeed, the diets assessed in these studies included large amounts of industrialised products (e.g., cakes, cookies, pastries), which are not typical of traditional diets [137,145,146]. While a traditional dietary pattern can include both healthy and unhealthy foods [147], it is not clear whether the proportions of these products are compatible with a traditional diet in Mexico. As such, the inclusion of non-traditional products in the TMexD’s definition may have also underestimated the association with health outcomes. Similarly, although the cohort study and the RCT evaluated the role of a diet high in maize, beans, rice, fruits, vegetables, and dairy [78,79], they did not include other potential key ingredients of the TMexD, such as herbs, condiments, and beverages. Future studies that use a more comprehensive definition of the TMexD and adhere to guidelines regarding the risk of bias are warranted. 

## 5. Conclusions

The present study systematically reviewed the evidence, for the first time, to establish the definition of the TMexD and evaluate its relationship with NCD outcomes. By conducting a systematic review of the literature, we provided a comprehensive description of this diet and evaluated the foods consistently characterised as traditional Mexican. The findings suggest that the TMexD is composed of grains, legumes, and vegetables, while specific food items include mainly maize, beans, and *chile*. Additionally, maize products, fruits, beverages, fish and seafood, meats, sweets and sweeteners, and herbs and condiments might still play an important role in the TMexD, although these might be considered complementary to the previously mentioned foods. The inclusion of oils and fats and nuts and seeds could still also be considered when defining the TMexD, as these items were still mentioned, even if infrequently. However, if the aim is to promote the TMexD as a public health strategy, more research is needed to evaluate the feasibility of including some foods that are not usually consumed by the general population (i.e., insects and reptiles, or some meats and plant-based foods that are no longer common in Mexico) and foods that were more recent inclusions to the Mexican diet (i.e., eggs, dairy, and processed foods). 

Findings regarding the TMexD’s association with health outcomes should be interpreted with caution. While we reported the findings following standard guidelines and evaluated the studies’ risk of bias and quality of reporting, the small number of studies, the high degree of heterogeneity between the studies included, the potential risk of bias in some studies, and the different TMexD definitions used hindered reaching strong conclusions on whether the TMexD is associated with health outcomes. 

Nevertheless, the current findings provide a detailed description of the TMexD, which could be used as a reference to promote the ingredients contained in a TMexD or as a reference to evaluate this traditional diet’s association with NCD outcomes. However, the present definition is limited by the lack of specific amounts in which these foods are consumed in the TMexD, particularly if the health properties of this diet are to be assessed. Elucidating the TMexD and operationalising it in the form of a dietary index would allow more robust associations with health outcomes to be established in future research, with potentially important implications for NCD prevention in Mexico.

## Figures and Tables

**Table 1:** Characteristics of included studies.

First Author (ref.)	Year	Country	Publication Format	Study Design	Years or Period the Data Represent	Geographical Location of the Diet *	Population Represented	Assessment Method Used to Define Diet ^†^
Literature Reviews								
Aguirre-Beltrán [47]	1994	Mexico	Book	Narrative review	16th century (precolonial period)	NS (Not specified)	Indigenous population	Historical and anthropological data
Allen [48]	1992	US and Mexico	Journal	Narrative review	Precolonial and colonial period	Mexico (all regions)	Indigenous population	Literature review ^1^
Algert et al. [49]	1998	US	Book	Narrative review	NS	Mexico (all regions)	NS	Literature review ^1^
Almaguer-González [50]	2018	Mexico	Ministry of Health report	Narrative review	Colonial period	Mexico (all regions)	NS	Literature review ^1^
Avila-Nava [51]	2017	Mexico	Journal	Experimental study ^2^	Precolonial period	Mesoamerican region (Central and Southern Mexico)	Aztec Indians	Literature review ^1^
Barros [52]	1999	Mexico	Journal	Narrative review	Precolonial period	NS	Mexica Indians	Historical data
Berdan [53]	2017	United States	Published essay	Narrative review	Early 1500 (precolonial period)	Basin of Mexico (Central Mexico)	Aztec Indians	Literature review ^1^
Bertran-Vilà [54]	2010	Mexico	Journal	Narrative review	Present time	Mexico City (Central Mexico)	Rural and lower-class populations	Literature review ^1^
BertranVilà [55]	2005	Mexico	Book	Narrative review	Present time	Mexico (all regions)	Indigenous populations	Literature review ^1^
Bertran [56]	2006	Mexico	Book	Narrative review	NS	Mexico (all regions)	NS	Literature review ^1^
Casillas [57]	1984	Mexico	Book	Narrative review	Precolonial period	Central plateau (Central Mexico)	Mexica Indians	Historical and archaeological data
Castelló and Turbide [58]	1986	Mexico	Book	Narrative review	Precolonial period	Mesoamerican region (Central and Southern Mexico)	Indigenous population	Historical data
Cook [59]	1979	United States	Book	Narrative review	1500–1650 (precolonial period)	Central Mexico	Lower and middle class, indigenous population	Historical data
Dávalos Hurtado [60]	1995	Mexico	Journal	Narrative review	NS	Valley of Mexico (Central Mexico)	Mexica Indians	Historical data
Flores and Escalante [61]	2004	Mexico	Book	Narrative review	Precolonial and colonial period	Mexico (all regions)	NS	Literature review ^1^
García Urigüen [62]	2012	Mexico	Book	Narrative review	Precolonial and colonial period	Mesoamerican region (Central and Southern Mexico)	Indigenous populations	Historical data
Harris [63]	2004	US	Thesis	Narrative review	Colonial period	NS	NS	Literature review ^1^
Katz [64]	1990	France and Mexico	Journal	Narrative review	Precolonial period	Mixteca Alta (Southern Mexico)	Mixteca Indians	Archaeological, ethnographic, and historical data
Kittler [65]	2008	US	Book	Narrative review	Precolonial and colonial period	Mexico (all regions)	NS	Historical data
Llamas [66]	1935	Mexico	Book	Narrative review	Precolonial period	Tenochtitlan (Central Mexico)	Aztec Indians	Historical data
Long-Solís [67]	2005	US	Book	Narrative review	16^th^ century (colonial period)	Mexico (all regions)	NS	Historical and cultural data
López Alonso [68]	1974	Mexico	Book	Narrative review	Precolonial period	Mexico (all regions)	Indigenous population	Historical and archaeological data
Márquez Morfin [69]	1991	Mexico	Journal	Narrative review	Precolonial period	Yucatán (Southern Mexico)	Maya Indians	Historical and archaeological data
Méndez y Mercado [70]	1993	Mexico	Journal	Narrative review	Precolonial and colonial period	La Mixteca (Southern Mexico)	Mixteca Indians	Historical data
Ojeda-Granados [71]	2017	Mexico	Journal	Cross-sectional study	5000 years ago (precolonial period)	Mesoamerican region (Central and Southern Mexico)	Indigenous population	Literature review ^1^
Ortiz de Montellano [72]	1990	US	Book	Narrative review	Precolonial period	Central Mexico	Aztec Indians	Historical data
Quevedo [73]	2004	Mexico	Book	Narrative review	Precolonial period	Tenochtitlan (Central Mexico)	Indigenous populations	Experience and oral tradition^3^
Quiñonez Tapia [74]	2019	Mexico	Journal	Narrative review	NS	Jalisco, Nayarit, Durango, Zacatecas (Northern and Central Mexico)	Wixárika Indians	Literature review ^1^
Quiroz [75]	2005	Mexico	Book	Narrative review	18^th^ century (postcolonial period)	Mexico City (Central Mexico)	NS	Historical data
Román [76]	2013	Mexico	Journal	Narrative review	Precolonial period	Mexico (all regions)	Indigenous populations	Literature review ^1^
Romero Gwynn [77]	1994	US	Book	Narrative review	From precolonial to 1860	Tenochtitlan (Central Mexico)	Aztec Indians	Historical data
Santiago-Torres [78]	2015	US	Journal	Prospective cohort study ^2^	NS	NS	Indigenous and rural population	Historical and scientific data
Santiago-Torres [79]	2016	US	Journal	Randomised crossover feeding trial ^2^	From precolonial period up to 1940	NS	NS	Historical and scientific data
Santley [80]	1979	US	Journal	Narrative review	Precolonial period	Basin of Mexico (Central Mexico)	Aztec Indians	Archaeological data
Shamosh [81]	2014	Mexico	Thesis	Narrative review	Precolonial, colonial and independent period	Mexico (all regions)	NS	Historical and archaeological data
Soustelle [82]	1970	France	Book	Narrative review	Precolonial period	Valley of Mexico (Central Mexico)	Aztec Indians	Historical data
UNESCO [83]	2010	Mexico	Report	NS	NS	Michoacán (Central Mexico)	NS	NS
Vargas [84]	1984	Mexico	Book	Narrative review	Precolonial period	Tabasco, Campeche, Yucatán, Quintana Roo and Chiapas (Southern Mexico)	Maya Indians	Historical and archaeological data
Vargas [85]	1988	Mexico	Book	Narrative review	Precolonial period	Mexican settlement (Central Mexico)	Mexican Indians	Historical data
Vargas [86]	2003	Mexico	Book	Narrative review	16^th^ century (precolonial period)	Mesoamerican region (Central and Southern Mexico)	Indigenous populations	Historical data
Velasco Lozano [87]	1995	Mexico	Book	Narrative review	Precolonial period	Valley of Mexico (Central Mexico)	Mexican Indians	Historical data
Wentworth [88]	1936	NS	Journal	Experimental study ^2^	NS	NS	Indigenous populations	NS
Wicke [89]	1959	Mexico	Book	Narrative review	16th century (precolonial period)	Tlatelolco-Tenochtitlan (Central Mexico)	Aztec Indians	Literature review ^1^
**Original studies**								
Anderson [90]	1946	Mexico and US	Journal	Cross-sectional study	1943–1944	Mezquital Valley (Southern Mexico)	966 Otomi Indians	One-week records of food consumption
Beals [91]	1943	US	Journal	Cross-sectional study	1941–1942	Michoacán (Central Mexico)	Tarascan Indians	List of foods, menus, and food recalls in a 15-day period
Burgos-Monzon [92]	2013	US	Thesis	Cross-sectional study	2010–2012	Texas (Southern US)	605 MA aged >18 y (mean age: 44 ± 13 ^4^ y)	FFQ + factor analysis
Carrera [93]	2007	US	Journal	Cross-sectional study	2001–2002	US (all regions)	659 MA aged >18 y (mean age: 36–39 y)	24-hour recall and cluster analysis
Crocker Sagastume [94]	2004	Mexico	Journal	Qualitative study	1999	Jalisco, Nayarit, Durango, Zacatecas (Northern and Central Mexico)	Wixárica Indians	Observation and in-depth interviews
Flores [95]	2010	Mexico and US	Journal	Cross-sectional study	2006	Mexico (all regions)	15,890 adults aged 20–59 y (mean age: 38 ^4^ y)	FFQ and cluster analysis
García-Chávez [96]	2017	Mexico and US	Journal	Cross-sectional study	2012	Mexico (all regions)	2751 children aged 5–11 y (mean age: 9 ^4^ ± 2 y)	24-hour recall + cluster analysis
McMurry [97]	1991	US	Journal	Clinical intervention	NS	Chihuahua (Northern Mexico)	13 Tarahumara Indians aged 12–35 y	Observed diet of the population
Mercado [98]	2012	US	Thesis	Qualitative study	NS	NS	10 MA aged 20–80 y	Focus group discussion on traditional diet
Moreno-Altamirano [99]	2017	Mexico and Italy	Journal	Ecological study	1961–1968	Mexico (all regions)	NS	Cluster analysis from the FAO Food Balance Sheet
Murtaugh [100]	2008	US	Journal	Case-control study	1999–2004	Arizona, New Mexico, Colorado, Utah (US)	4746 women aged 25–79 y	FFQ + factor analysis
Ravussin [101]	1994	US and Mexico	Journal	Cross-sectional study	1991	Maycoba, Sonora (Northern Mexico)	35 Pima Indians aged 17–74 y (mean age: 36–48 y)	FFQ
Rendón [102]	1947	Mexico	Journal	Qualitative study	1941–1942	Area of La Sierra (Central Mexico)	Tarascan Indians	Direct observation
Robles-Ordaz [103]	2017	Mexico	Journal	Cross-sectional study	2014–2015	Sonora (Northern Mexico)	227 Comcáac Indians aged >20 y	FFQ + principal component analysis
Rodríguez-Morán [104]	2009	Mexico	Journal	Two-stage cross-sectional study	1995–1996	Sierra Madre Occidental (Northern Mexico)	119 Tepehuano Indian adults	FFQ
Tseng [105]	1997	US	Thesis	Cross-sectional study	1988–1994	US (all regions)	4641 MA aged 20–74 y (mean age: 36–37 y)	FFQ + principal component analysis
Weitlaner [106]	1952	Mexico	Journal	Qualitative study	1935–1943	Oaxaca (Southern Mexico)	Chinanteca Indians	Direct observation
Wyatt [107]	1998	Mexico	Journal	Cross-sectional study	NS	Sonora (Northern Mexico)	550 adults aged >25 y	24-hour recall

* The areas in parentheses correspond to the geographical classifications used in the National Health and Nutrition Survey from Mexico [40]. * Historical data consisted of Spanish manuscripts describing the indigenous food habits before the Mexican colonisation; archaeological data consisted of remains of foods and cooking instruments; ethnographic data consisted of direct observations of diets of indigenous populations.^1.^ No methodology reported but referencing >1 sources. ^2.^ Definition of the diet located in Introduction/Methods. ^3.^ Terms used by the authors cited. ^4.^ Rounded to the nearest whole number. FAO: Food and Agriculture Organisation. FFQ: Food frequency questionnaire. MA: Mexican Americans. NS: Not specified. US: United States. Y: years.

**Table 2:** Most cited items and food groups present in all included studies.(*n* = 61)

	**(a) Food groups present in at least 75% of the studies, including their most cited items**									
**Grains and tubers**	**Maize products**		**Legumes**	**Vegetables**		**Fruits**		**Meats**		**Herbs and condiments**
Maize ^1^, amaranth, rice, wheat (as bread, pasta, tortillas) ^2^ potato, sweet potato	Tortillas ^1^, tamales, *atole* ^3^		Beans ^1^	Squash ^1^, c*hayote, nopales,* tomato ^1^, *tomatillo, quelites*		*Anona, capulín*, citrus fruits ^2,4^, guava, *jícama, mamey,* plums, prickly pear, z*apote*		Turkey, chicken, ducks, venison, rabbit dog, armadillo, beef		*Chile*^1^, salt, onion
	**(b) Food groups present in at least 50% of the studies, including their most cited items**									
**Oils and fats**		**Nuts and seeds**			Beverages *		**Fish and seafood**		**Sweets and sweeteners**	
Avocado ^5^		Pumpkin seeds, chia seeds			Chocolate drinks ^1^, *pulque* ^6^		- ^7^		Honey ^8^, sugar, and sugarcane	

* Excluding maize-based drinks. 1. Individual items (not based on food groupings) were also present in at least 50% of all studies. 2. These items were grouped, as some records did not specify the presentation/specific food consumed. 3. Hot beverage prepared with maize dough. 4. Includes orange, mandarin, grapefruit, lemon, and lime. 5. Referring to the avocado fruit. 6. Fermented maguey drink. 7. None of the individual items was highly cited; only the overall food group. 8. Including bee, ant, wasp, maize, maguey, and nopal honey.

**Table 3:** Most cited items and food groups present in literature reviews. (n = 43)

**(a) Food groups present in at least 75% of the studies, including their most cited items**												
**Grains**	**Maize products**		**Legumes**		**Vegetables**		**Fruits**	Beverages *		**Meats**		**Herbs and condiments**
Maize ^1^, amaranth, wheat (as bread, pasta, tortillas) ^2^, potato, sweet potato, yucca	Tortillas ^1^, *tamales* ^1,3^, *atole* ^1,4^		Beans ^1^		Squash ^1^, c*hayote, nopales* ^1^, maguey, tomato ^1^, *tomatillo, quelites,* mushrooms, S*pirulina* algae		*Anona, capulín,* citrus fruits ^2,5^, guava, *guanábana, jícama, mamey,* papaya, pineapple, plums, *tejocote,* prickly pear, z*apote* ^1^	Chocolate drinks ^1^, *pulque* ^6^		Turkey ^1^, chicken, ducks,venison ^1^, rabbit, hare, dogs, armadillo, *tlacuache* ^7^, boar, beef, pork		*Chile*^1^, *epazote*, onion, salt, vanilla
**(b) Food groups present in at least 50% of the studies, including their most cited items**												
**Oils and fats and**		**Nuts and seeds**		**Fish and seafood**		**Insects**			**Reptiles**		**Sweets and sweeteners**	
Avocado ^1,8^		Peanuts, pumpkin seeds, chia seeds		Shrimp		Grasshoppers and locusts, maguey worms ^9^, ants and their larvae ^10^			Snakes, turtles, iguana		Honey ^1,11^	

* Excluding maize-based drinks. 1. Individual items (not based on food groupings) were also present in at least 50% of all studies. 2. These items were grouped, as some records did not specify the presentation/specific food consumed. 3. Traditional dish prepared with maize dough. 4. Hot beverage prepared with maize dough. 5. Includes orange, mandarin, grapefruit, lemon, and lime. 6. Fermented maguey drink. 7. Opossum. 8. Referring to the avocado fruit. 9. Includes *chinocuiles*. 10. Also known as *chicatanas*, *escamoles*. 11. Including bee, ant, wasp, maize, maguey, and *nopal* honey.

**Table 4:** Most cited items and food groups present in original studies. (*n* = 18).

**(a) Food groups present in at least 75% of the studies, including their most cited items.**											
**Grains and tubers**		**Legumes**	**Vegetables**		**Fruits**		**Dairy**		**Meats**		**Herbs and condiments**
Maize ^1^, rice, wheat (as bread, pasta, tortillas) ^1,2^, potato		Beans ^1^	Squash, tomato		Banana, citrus fruits ^2,3^		Milk ^1^, cheese		- ^4^		*Chile*^1^, onion
**(b) Food groups present in at least 50% of the studies, including their most cited items**											
**Maize products**	**Oils and fats**			Beverages*		**Fish and seafood**		**Eggs**		**Sweets and sweeteners**	
Tortillas ^1^, *atole* ^5^	Avocado, vegetable oil			Coffee, soda, tea		- ^4^		- ^4^		Sugar and sugarcane	

* Excluding maize-based drinks. 1. Individual items (not based on food groupings) were also present in at least 50% of all studies. 2. These items were grouped, as some records did not specify the presentation/specific food consumed. 3. Includes orange, mandarin, grapefruit, lemon, and lime. 4. None of the individual items was highly cited; only the overall food group. 5. Hot beverage prepared with maize dough.

**Table 5:** Most cited items and food groups present in studies referring to Northern Mexico (*n* = 7).

**(a) Food groups present in at least 75% of the studies, including their most cited items**						
**Grains and tubers**		**Legumes**		**Vegetables**	**Fruits**	**Eggs**
Maize ^1^, amaranth, rice, wheat (as bread, pasta, tortillas) ^2^, potato		Beans ^1^		Squash, tomato ^1^, *nopales*, *guaje*, *quelites*, mushrooms	Banana, citrus fruits ^2,3^, prickly pear	- ^4^
**(b) Food groups present in at least 50% of the studies, including their most cited items**						
**Maize products**	Beverages *		**Fish and seafood**	**Meats**	**Sweets and sweeteners**	**Herbs and condiments**
Tortillas ^1^, *pinole* ^5^	Beer, coffee, soda		- ^4^	Chicken	Sugar and sugarcane	*Chile*^1^, onion

* Excluding maize-based drinks. 1. Individual items (not based on food groupings) were also present in at least 50% of all studies. 2. These items were grouped, as some records did not specify the presentation/specific food consumed. 3. Includes orange, mandarin, grapefruit, lemon, and lime. 4. None of the individual items was highly cited; only the overall food group. 5. Maize flour, occasionally sweetened and mixed with cacao, cinnamon, or anise.

**Table 6:** Most cited items and food groups present in studies referring to Central Mexico (*n* = 24).

**(a) Food groups present in at least 75% of the studies, including their most cited items**												
**Grains and tubers**	**Maize products**		**Legumes**		**Vegetables**		Beverages *	**Fish and seafood**	**Meats**		**Herbs and condiments**	
Maize ^1^, amaranth ^1^, sweet potato	Tortillas ^1^, *tamales* ^1,2^, *atole* ^1,3^		Beans ^1^		Squash ^1^, c*hayote, nopales,* maguey, tomato ^1^, *tomatillo* *huauzontle, mezquite, quelites* ^1^, mushrooms, S*pirulina* algae		Chocolate drinks ^1^, *pulque* ^1,4^, *pozol ^5^*	Shrimp	Turkey ^1^, chicken, ducks, venison ^1^, rabbit ^1^, hare, dogs ^1^, gopher, armadillo, boar, *tlacuache* ^6^		*Chile*^1^, *epazote*, onion, salt, vanilla	
**(b) Food groups present in at least 50% of the studies, including their most cited items**												
**Fruits**		**Oils and fats**		**Nuts and seeds**		**Insects**				**Reptiles**		**Sweets and sweeteners**
*Anona, capulín,* guava, *jícama, mamey,* plums, *tejocote,* prickly pear ^1^, *zapote* ^1^		Avocado ^1,7^		Peanuts, pumpkin seeds, chia seeds		Maguey worms ^8^, ants and their larvae ^9^, *amoyotl* ^10^, *ahuahutle* ^11^				Snakes, turtles, iguana		Honey ^1,12^

* Excluding maize-based drinks. 1. Individual items (not based on food groupings) were also present in at least 50% of all studies. 2. Traditional dish prepared with maize dough. 3. Hot beverage prepared with maize dough. 4. Fermented maguey drink. 5. Fermented maize drink. 6. Opossum. 7. Referring to the avocado fruit. 8. Includes *chinocuiles.* 9. Also known as *chicatanas*, *escamoles*. 10. Water fly. 11. *Axayacatl* (water-fly) eggs. 12. Including bee, ant, wasp, maize, maguey, and *nopal* honey.

**Table 7:** Most cited items and food groups present in studies referring to Southern Mexico (*n* = 11).

**(a) Food groups present in at least 75% of the studies, including their most cited items**													
**Grains and tubers**	**Legumes**	**Vegetables**		**Fruits**	**Oils and fats**		**Nuts and seeds**		Beverages *		**Meats**	**Insects**	**Herbs and condiments**
Maize ^1^, amaranth ^1^, sweet potato, yucca	Beans ^1^	Squash ^1^, c*hayote, nopales* ^1^, tomato ^1^, *tomatillo* ^1^*, guaje, huauzontle, mezquite,* *papaloquelite,* purslane, *quelites* ^1^, *quintoniles*, *Setaria,* mushrooms, S*pirulina* algae		*Anona, capulín*^1^, guava, *guanábana, jícama, mamey*^1^, *nanche,* papaya, plums ^1^, *ramón, tejocote,* prickly pear ^1^, *zapote* ^1^	Avocado ^1,2^, lard, animal fats		Pumpkin seeds, chia seeds		Chocolate drinks ^1^, *pulque* ^1,3^, *pozol* ^4^		Turkey ^1^, partridges, ducks, venison ^1^, rabbit ^1^, hare, pork, dogs, armadillo ^1^, squirrel, boar, gopher	Grasshoppers and locust, ants and their larvae ^5^	*Acedera*^6^, *acuyo*^7^, *chile*^1^, *chipilín*, *epazote*, onion, salt ^1^
**(b) Food groups present in at least 50% of the studies, including their most cited items**													
**Maize products**			**Fish and seafood**					**Reptiles**		**Sweets and sweeteners**			
Tortillas ^1^, *tamales* ^8^, *atole* ^1,9^, *pinole* ^10^			Catfish, shrimp					Turtles, iguana, lizard		Honey ^1,11^			

* Excluding maize-based drinks. 1. Individual items (not based on food groupings) were also present in at least 50% of all studies. 2. Referring to the avocado fruit. 3. Fermented maguey drink. 4. Fermented maize drink. 5. Also known as *chicatanas*, *escamoles*. 6. Also known as *lengua de vaca*. 7. Also known as *hierbasanta*. 8. Traditional dish prepared with maize dough. 9. Hot beverage prepared with maize dough. 10. Maize flour, occasionally sweetened and mixed with cacao, cinnamon or anise. 11. Including bee, ant, wasp, maize, maguey, and *nopal* honey.

**Table 8:** Most cited items and food groups present in studies referring to all regions of Mexico (*n* = 14).

**(a) Food groups present in at least 75% of the studies, including their most cited items**												
**Grains and tubers**	**Maize products**	**Legumes**		**Vegetables**	**Fruits**		**Oils and fats**		Beverages *	**Meats**	**Sweets and sweeteners**	**Herbs and condiments**
Maize ^1^, amaranth, rice ^1^, wheat (as bread, pasta, tortillas) ^1,2^, potato ^1^, sweet potato, yucca	Tortillas ^1^, *tamales* ^1,3^, *atole* ^1,4^, soups (*pozole* ^5^, *menud*o ^6^), others ^7^	Beans ^1^		Squash ^1^, *chayote* ^1^*, nopales* ^1^, tomato ^1^, *tomatillo* ^1^, carrot, lettuce, purslane, *quelites ^1^*, *quintoniles*, mushrooms, *huitlacoche*, squash blossoms	*Anona,* apple, banana, berries ^2^, *capulín,* citrus fruits ^1,2,8^, guava ^1^, *guanábana*, *jicama* ^1^, *mamey*, mango, melon, papaya, peach, pear, pineapple ^1^, *pitahaya*, plums, *tejocote,* prickly pear, *zapote*		Avocado ^1,9^, vegetable oil, cream		Chocolate drinks ^1^, *pulque* ^10^*, tesgüino* ^11^, coffee, *aguas frescas* ^12^, natural fruit juice	Turkey ^1^, chicken ^1^, venison, pork, rabbit, beef ^1^, lamb, chevon, dogs	Honey ^1,13^ *Pan dulce* ^14^, sugar and sugarcane, desserts, sweets	*Annato*, *acuyo*^15^, *chile*^1^, coriander, *epazote*, garlic, onion ^1^, parsley, pepper, vanilla
**(b) Food groups present in at least 50% of the studies, including their most cited items**												
**Nuts and seeds**			**Fish and seafood**			**Dairy**		**Eggs**			**Insects**	
Peanuts, pumpkin seeds ^1^, chia seeds, sesame seeds			Shrimp			Cheese ^1^, milk		Chicken eggs			Grasshoppers and locusts, ants and their larvae ^16^	

* Excluding maize-based drinks. 1. Individual items (not based on food groupings) were also present in at least 50% of all studies. 2. These items were grouped, as some records did not specify the presentation/specific food consumed. 3. Traditional dish prepared with maize dough. 4. Hot beverage prepared with maize dough. 5. Soup made with maize kernels, meat, *chile*, and seasonings. 6. Beef tripe in broth with *chile*. 7. Includes tacos, popcorn, *sopes*, *pellizcadas*, *gorditas*, *tostadas*, *peneques*, and *totopos*. 8. Includes orange, mandarin, grapefruit, lemon, and lime. 9. Referring to the avocado fruit. 10. Fermented maguey drink. 11. Fermented maize drink. 12. Water blended with fruit/flowers and sugar. 13. Including bee, ant, wasp, maize, maguey, and *nopal* honey. 14. Sweet bread, a traditional pastry prepared with sugar and fat. 15. Also known as *hierba santa*. 16. Also known as as*chicatanas*, *escamoles*.

**Table 9:** Key characteristics and findings of the studies examining the association of the Traditional Mexican diet with health outcomes.

First Author (ref.)	Year (Country)	Population Characteristics	DP Assessment Method	DP Definition	Comparators	Follow up	Results (95% CI)	Covariates
Case-control studies								
Murtaugh [100]	2008 (US)	4746 women aged 25–79 y (mean not reported)	Dietary history questionnaire + factor analysis	Native Mexican ^1^ (highest quartile)	Native Mexican ^1^ (lowest quartile)	NA	OR breast cancer Highest vs. lowest quartile: 0.68 (0.55, 0.85), *p* < 0.01	Age, study centre, education, smoking, total activity, calories, dietary fibre and calcium, height, parity, hormone exposure, family history of breast cancer, BMI x hormone exposure
Cross-sectional studies								
Carrera [93]	2007 (US)	659 adults aged ≥18 y (mean: 36.1 to 38.7 y)	24 h recall + cluster analysis	Traditional Mexican ^2^	Poultry/alcohol ^3^ Milk/baked products ^4^ Meat ^5^	NA	Mean BMI Traditional Mexican: 28.3 ± 0.5 vs. poultry/alcohol: 28.2 ± 0.5, milk/baked products: 27.9 ± 0.4, meat: 27.9 ± 0.5; *p* > 0.05 Mean WC women Traditional Mexican: 93.1 ± 1.7 vs. poultry/alcohol: 94.2 ± 1.9, milk/baked products: 92.0 ± 1.2, meat: 95.2 ± 1.7; *p* > 0.05 Mean WC men Traditional Mexican: 97.8 ± 1.6 vs. poultry/alcohol: 95.8 ± 1.6, milk/baked products: 95.6 ± 1.3, meat: 94.2 ± 1.5; *p* > 0.05	Total energy intake, smoking, and physical activity
Flores [95]	2010 (Mexico)	15,891 adults aged 20–59 y (mean: 37.4 y)	FFQ + cluster analysis	Traditional ^6^	Refined/sweets ^7^ Diverse ^8^	NA	**OR overweight** Refined/sweets vs. traditional: 1.14 (1.02, 1.26) *p*: 0.01; Diverse vs. traditional: 1.17 (1.03,1.33) *p*: 0.01 **OR obesity** Refined/sweets vs. traditional: 1.20 (1.09, 1.31), *p*: 0.001; Diverse vs. traditional: 1.20 (1.08, 1.34), *p*: 0.001	Age, gender, physical activity, socio-economic status, area, and region
Robles- Ordaz [103]	2017 (Mexico)	227 adults aged >20 y (mean not reported)	FFQ + principal component analysis	Traditional ^9^	Western ^10^ Hypercaloric ^11^	NA	**OR prediabetes** Traditional vs. Western and hypercaloric: 0.49 (0.31, 0.79), *p*: 0.003	Age and sex
**Cohort studies**								
Santiago- Torres [78]	2015 (US)	476 women aged 50–79; mean: 59 ± 6.3 y	FFQ + ‘Traditional Mexican diet score’	Mexican diet ^12^ (high scores)	Mexican diet ^12^ (low and medium scores)	15.4± 1.1y	**Mean glucose, mg/dL** High 98.7 ± 20.5 vs. low 97.3 ± 17.3; *p* > 0.05 **Mean insulin, μIU/mL** High 12.2 ± 10.7 vs. low 14.0 ± 11.1 (↓15%); *p* < 0.05 **Mean HOMA-IR** High 3.13 ± 3.31 vs. low 3.49 ± 3.36; *p* > 0.05 **Mean TG, mg/dL** High 123 ± 58.4 vs. low 125 ± 49.3; *p* > 0.05	Age, BMI, total energy intake, education level, acculturation, and baseline biomarker concentration levels
**Randomised crossover feeding trials**								
Santiago- Torres [79]	2016 (US)	53 women aged 18–45 (mean: 27± 6.8 y)	7-day menu and food dairies of adherence	Mexican diet ^13^	US diet ^14^	Diet for 24 days plus 28-day washout period	**Mean difference glucose, mg/dL** 0.82, *p*: 0.42 **Mean difference insulin, μU/mL** 1.26, *p*: 0.02 (↓ 14%) **Mean difference HOMA-IR** 0.30, *p*: 0.02 (↓15%) **Mean difference IGF-1, ng/mL** 5.72, *p*: 0.06 (↓ 4%) **Mean difference IGFBP-3, ng/mL** 121, *p* < 0.01 (↓ 6%) **Mean difference IGF-1:IGFBP-3** –0.09, *p*: 0.38	Diet sequence, feeding period, baseline and washout biomarker concentrations, age, acculturation, and BMI

The following underlined descriptions refer to descriptions of Mexican diets. 1: Mexican cheeses, soups, meat dishes, legumes, tomato-based sauces. 2: *Tortillas* and *tacos* flavoured and sweetened drinks, legumes, red meat, eggs, cakes and cookies, milk, non-citrus fruits. 3: Poultry, flavoured and sweetened drinks, alcoholic beverages, bread and wheat products, cakes and cookies. 4: Flavoured and sweetened drinks, cakes and cookies, milk, tortillas and tacos, pizza, bread and wheat products, and soups. 5: Red meat, flavoured and sweetened drinks, tortillas and tacos, and bread and wheat products. 6: Maize tortillas and maize-based foods, alcohol, Mexican snacks, sugar-sweetened beverages, and white bread and wheat tortillas. 7: Maize tortillas, maize-based foods, alcohol, sugar-sweetened beverages, white bread and wheat tortillas, Mexican snacks, dairy products, sweet bread, cookies, fast food, and red meat. 8: Maize tortillas, maize-based foods, whole-fat dairy products, alcohol, SSBs, white bread and wheat tortillas, fresh fruit, Mexican snacks, rice and pasta, high fibre, ready-to-eat cereal, and sweet bread. 9: Fish and seafood, low-fat cereals, fruits and vegetables. 10: Meat, chicken, desserts, and processed meat. 11: Beverages, legumes, tortilla*s*. 12: High in tortillas, beans, soups, Mexican dishes, fruits, vegetables, rice, full-fat milk and cheeses. Low in oil, solid fat, sugar, processed meats, and refined grains. 13: Beans, corn *tortillas*, traditional Mexican soups and Mexican mixed dishes, citrus fruits, vegetables, animal fats, full-fat milk, and *aguas frescas* (water blended with fruits). 14: Refined grains, vegetable oils, non- or low-fat milk, processed foods, processed meats, sugar-sweetened beverages, and grain-based desserts. BMI: Body mass index. CI: Confidence intervals. DP: Dietary pattern. FFQ: Food frequency questionnaire. HOMA-IR: Homeostasis model assessment of insulin resistance. IGF-1: Insulin-like growth factor. IGFBP-3: Insulin-like growth factor binding protein 3. NA: Non-applicable. OR: Odds ratio. SD: Standard deviation. TG: Triglycerides. US: United States. WC: Waist circumference.

## Supplementary Materials

The following are available online at https://www.mdpi.com/2072-6643/11/11/2803/s1, Supplementary Material I, Table S1: PRISMA statement, Table S2: Data extraction form, Table S3: Methodology used to categorise foods in the traditional Mexican diet in all included studies, Table S4: List of excluded articles, Table S5: Definition of the traditional Mexican diet according to different authors, Table S6 (a)(b)(c): Amounts of foods consumed in the traditional Mexican diet according to different authors, Table S7: Quality assessment of the included articles, Figure 1: Risk of bias assessment of the included articles, Table S8: Reporting quality assessment of the included articles, based on the STROBE 2007 checklist, Table S9: Reporting quality assessment of the included articles, based on the CONSORT 2010 checklist, Figure S2: Proportion of reported items in observational studies, based on the STROBE 2007 checklist, Table S10. Food groups mentioned in the different subgroups evaluated, Table S11. Individual foods mentioned in the different subgroups evaluated. Supplementary Material 2, Search strategy.
