# Supplementary material for: Definition of the Traditional Mexican Diet and Its Role in Health: A Systematic Review
Source: Nutrients. 2019 Nov 17;11(11):2803. doi: 10.3390/nu11112803 (PMC6893605; doi:10.3390/nu11112803)
Supplement: Supplementary file 1 [file nutrients-11-02803-s001.pdf]

Supplementary Materials I

Table S1. PRISMA statement

Section/topic	#	Checklist item	Reported on page #
**TITLE**			
Title	1	Identify the report as a systematic review, meta-analysis, or both.	1
**ABSTRACT**			
Structured summary	2	Provide a structured summary including, as applicable: background; objectives; data sources; study eligibility criteria, participants, and interventions; study appraisal and synthesis methods; results; limitations; conclusions and implications of key findings; systematic review registration number.	1
**INTRODUCTION**			
Rationale	3	Describe the rationale for the review in the context of what is already known.	1-2
Objectives	4	Provide an explicit statement of questions being addressed with reference to participants, interventions, comparisons, outcomes, and study design (PICOS).	2
**METHODS**			
Protocol and registration	5	Indicate if a review protocol exists, if and where it can be accessed (e.g., Web address), and, if available, provide registration information including registration number.	2
Eligibility criteria	6	Specify study characteristics (e.g., PICOS, length of follow-up) and report characteristics (e.g., years considered, language, publication status) used as criteria for eligibility, giving rationale.	2-4
Information sources	7	Describe all information sources (e.g., databases with dates of coverage, contact with study authors to identify additional studies) in the search and date last searched.	2
Search	8	Present full electronic search strategy for at least one database, including any limits used, such that it could be repeated.	Supplementary Materials II
Study selection	9	State the process for selecting studies (i.e., screening, eligibility, included in systematic review, and, if applicable, included in the meta-analysis).	4
Data collection process	10	Describe method of data extraction from reports (e.g., piloted forms, independently, in duplicate) and any processes for obtaining and confirming data from investigators.	4
Data items	11	List and define all variables for which data were sought (e.g., PICOS, funding sources) and any assumptions/simplifications made.	4 and Supplementary

			Materials I Table S2
Risk of bias in individual studies	12	Describe methods used for assessing risk of bias of individual studies (including specification of whether this was done at the study or outcome level), and how this information is to be used in any data synthesis.	4-5
Summary measures	13	State the principal summary measures (e.g., risk ratio, difference in means).	4
Synthesis of results	14	Describe the methods of handling data and combining results of studies, if done, including measures of consistency (e.g., I2) for each meta-analysis.	4
Risk of bias across studies	15	Specify any assessment of risk of bias that may affect the cumulative evidence (e.g., publication bias, selective reporting within studies).	5
Additional analyses	16	Describe methods of additional analyses (e.g., sensitivity or subgroup analyses, meta-regression), if done, indicating which were pre-specified.	4-5
**RESULTS**			
Study selection	17	Give numbers of studies screened, assessed for eligibility, and included in the review, with reasons for exclusions at each stage, ideally with a flow diagram.	6
Study characteristics	18	For each study, present characteristics for which data were extracted (e.g., study size, PICOS, follow-up period) and provide the citations.	8-14
Risk of bias within studies	19	Present data on risk of bias of each study and, if available, any outcome level assessment (see item 12).	Supplementary materials Table S7; and Supplementary materials Figure 1
Results of individual studies	20	For all outcomes considered (benefits or harms), present, for each study: (a) simple summary data for each intervention group (b) effect estimates and confidence intervals, ideally with a forest plot.	Supplementary materials Table S3 and S5; 24-26
Synthesis of results	21	Present results of each meta-analysis done, including confidence intervals and measures of consistency.	15 and 23
Risk of bias across studies	22	Present results of any assessment of risk of bias across studies (see Item 15).	22 and 27
Additional analysis	23	Give results of additional analyses, if done (e.g., sensitivity or subgroup analyses, meta-regression [see Item 16]).	15-21
**DISCUSSION**			
Summary of evidence	24	Summarize the main findings including the strength of evidence for each main outcome; consider their relevance to key groups (e.g., healthcare providers, users, and policy makers).	27-30

Limitations	25	Discuss limitations at study and outcome level (e.g., risk of bias), and at review-level (e.g., incomplete retrieval of identified research, reporting bias).	31-32
Conclusions	26	Provide a general interpretation of the results in the context of other evidence, and implications for future research.	32
**FUNDING**			
Funding	27	Describe sources of funding for the systematic review and other support (e.g., supply of data); role of funders for the systematic review.	33

From: Moher D, Liberati A, Tetzlaff J, Altman DG, The PRISMA Group (2009). Preferred Reporting Items for Systematic Reviews and Meta-Analyses: The PRISMA Statement. PLoS Med 6(7): e1000097.

Table S2. Data extraction form

**Traditional Mexican diet definition**	**Association between the traditional Mexican diet and health outcomes**
Author	Author
Year	Year and country
Country	Study design
Journal title or publication format	Population characteristics
Study design	Dietary pattern assessment method
Years/period represented	Dietary pattern definition
Geographical location represented	Comparators
Population represented	Time point of measurement
Diet assessment method	Results
Dietary pattern description	Covariates

Table S3. Methodology used to categorise foods in the traditional Mexican diet in all included studies

First author	Year	Main foods																										Limited foods									
		Maize	Beans	Chile or salsas	Squash	Vegetables (inc. Greens, nopal, maguey tomato)	Fruits (incl. Tuna)	Amaranth	Pulque and fermented beverages	Avocado	Milk cheese	Meat and organs	Peas, lentils, ayocotes and other legumes	Soups or stews	Fish and seafood (inc. charales)	Batrachian	Eggs	Roots	Pumpkin, chia and other seeds	Other grains/ Wheat and rice	Vanilla and chocolate	Turkey or chicken	Onion	Insects	Honey	Coffee/soda	Sage	Meats	Fruits	Pulque and alcoholic drinks	Oil	Solid fats	Desserts	Addedd sugars	White bread and refined grains	Eggs	Milk
Aguirre-Beltran	1994	1	1	1	1	1																						1									
Allen	1992																													1							
Almaguer Gonzalez	2018	1	1	1	1																																
Algert	1998	1	1		1																																
Anderson	1946	1	1	1																								1				1					1
Avila-Nava	2017	1	1																1																		
Barros	1999																																				
Beals	1943																																				
Berdan	2017	1	1	1	1	1																															
Bertran-Vila	2010	1	1	1		1	1																					1									
Bertrán	2005	1	1	1		1						1					1											1	1	1						1	
Bertrán	2006	1	1		1																							1									
Burgos-Monzon	2013	1	1	1									1				1			1						1											
Carrera	2007																																				
Casillas	1984	1		1	1			1																													
Castelló Yturbide	1986	1	1	1	1																																
Cook	1980	1	1	1	1	1	1		1									1	1																		
Crocker Sagastume	2004	1	1		1																																
Davalos Hurtado	1994	1																																			
Flores	2010																																				
Flores y Escalante	2004	1	1	1	1	1	1		1	1								1			1	1															
García Chávez	2017																																				
Garcia Uriguen	2012																																				
Harris	2004																																				
Katz	1990	1	1	1	1	1	1																														
Kittler	2007																																				
Llamas	1935	1	1	1					1																												
Long-Solis	2005	1	1		1																												1				
Lopez Alonso	1974	1																																			
Márquez-Morfín	1991	1	1	1											1																						
McMurry	1991	1	1																																		
Méndez y Mercado	1993	1	1	1	1																													1		1	
Mercado	2012	1	1	1		1	1							1						1		1				1											
Moreno-Altamirano	2017																																				
Murtaugh	2008					1					1	1	1	1																							
Ojeda-Granados	2017	1	1		1																																
Ortiz de Montellano	1990	1	1	1	1	1		1																													
Quevedo	2004	1	1	1	1	1		1																													
Quiñones Tapia	2019	1	1		1																																
Quiroz	2004																																				
Ravussin	1994																																				
Rendon	1947	1				1									1																						
Robles-Ordaz	2017					1	1								1					1																	
Rodríguez Morán	2009																																				
Roman et al	2013																																				
Romero-Gwynn	1994																																				
Santiago-Torres	2015	1	1			1	1				1			1														1			1	1		1	1		
Santiago-Torres	2016																																				
Santley et al.	1979	1	1	1	1		1	1																													
Shamosh	2014	1	1	1	1	1	1	1	1	1			1								1		1														
Soustelle	1970	1	1	1		1		1	1							1			1		1			1	1		1	1									
Tseng	1997	1	1	1							1	1																									
UNESCO	2010																																				
Vargas	1984	1																																			
Vargas	1988	1																																			
Vargas	2003	1	1	1	1	1		1		1									1																		
Velasco	1995	1	1																																		
Weitlaner	1952																																				
Wentworth	1936																																		1		
Wicke	1959	1	1	1	1																							1									
Wyatt	1998																																				
		39	33	24	21	17	9	7	5	3	3	3	3	3	3	1	2	2	4	3	3	2	1	1	1	2	1	8	1	2	1	2	1	2	2	2	1

% of documents that mention item

Main foods: 90% 55 | 75% 46 | 50% 31 | 25% 15

Limited foods: 90% 54 | 75% 45 | 50% 30 | 25% 15

First author	Year	Grains and tubers																						Total mentions (grains and tubers)
		Grains												Roots and tubers (excluding *jícama*)										
		Unspecific	Amaranth	Maize	Rice	Wheat bread, pasta or tortillas	Rye	Barley	Oat	Millet	Quinoa	Breakfast cereals	Total mentions (grains)	Unspecific	Chinchayote/ chayote root	Potato	Sweet potato (yams, boniato)	Yuca (guacamote, mandioca, cassava)	Others*: Oca, olluco, añu, ñame, boniato, macal, flor de tigre, pochote	Ayatito	Tule	Malanga	Total mentions (tubers)	
Aguirre-Beltran	1994			1		1							2										0	2
Allen	1992			1	1	1		1	1				5			1							1	6
Almaguer Gonzalez	2018		1	1									2		1		1	1					3	5
Algert	1998		1	1									2	1									1	3
Anderson	1946			1									1										0	1
Avila-Nava	2017			1									1										0	1
Barros	1999		1	1									2			1	1						2	4
Beals	1943			1									1										0	1
Berdan	2017			1									1										0	1
Bertran-Vila	2010			1									1										0	1
Bertrán	2005			1	1	1							3			1	1	1					3	6
Bertrán	2006			1									1										0	1
Burgos-Monzon	2013			1	1	1							3										0	3
Carrera	2007	1		1		1						1	4	1		1	1						3	7
Casillas	1984		1	1									2										0	2
Castelló Yturbide	1986		1	1									2		1	1	1			1			4	6
Cook	1980			1									1				1						1	2
Crocker Sagastume	2004		1	1									2										0	2
Davalos Hurtado	1994			1									1			1	1	1					3	4
Flores	2010	1		1	1	1						1	5			1							1	6
Flores y Escalante	2004		1	1	1	1	1	1	1	1	1		9		1	1	1	1	1				5	14
García Chávez	2017	1		1	1	1			1			1	6	1		1							2	8
Garcia Uriguen	2012		1	1									2										0	2
Harris	2004			1	1	1							3			1							1	4
Katz	1990			1									1				1	1					2	3
Kittler	2007			1	1	1							3			1	1	1					3	6
Llamas	1935			1									1	1									1	2
Long-Solis	2005		1	1	1	1							4										0	4
Lopez Alonso	1974			1									1	1		1	1						3	4
Márquez-Morfín	1991			1									1	1				1	1				3	4
McMurry	1991			1									1										0	1
Méndez y Mercado	1993		1	1		1							3										0	3
Mercado	2012			1	1	1			1			1	5			1	1						2	7
Moreno-Altamirano	2017	1											1										0	1
Murtaugh	2008												0										0	0
Ojeda-Granados	2017		1	1									2										0	2
Ortiz de Montellano	1990		1	1									2										0	2
Quevedo	2004		1	1									2			1	1	1					3	5
Quiñones Tapia	2019		1	1									2										0	2
Quiroz	2004	1		1		1							3				1						1	4
Ravussin	1994			1	1	1							3			1							1	4
Rendon	1947			1	1	1							3	1	1								2	5
Robles-Ordaz	2017	1											1										0	1
Rodríguez Morán	2009					1							1	1		1							2	3
Roman et al	2013		1	1									2										0	2
Romero-Gwynn	1994			1	1	1		1					4				1						1	5
Santiago-Torres	2015	1		1	1							1	4										0	4
Santiago-Torres	2016			1	1								2										0	2
Santley et al.	1979		1	1									2										0	2
Shamosh	2014		1	1	1		1	1					5			1	1	1		1	1	1	6	11
Soustelle	1970		1	1									2										0	2
Tseng	1997			1									1										0	1
UNESCO	2010			1									1										0	1
Vargas	1984			1									1				1	1				1	3	4
Vargas	1988		1	1									2										0	2
Vargas	2003		1	1									2				1	1	1				3	5
Velasco	1995		1	1									2	1	1		1	1	1		1		6	8
Weitlaner	1952			1	1	1							3				1	1					2	5
Wentworth	1936					1							1										0	1
Wicke	1959		1	1									2				1	1					2	4
Wyatt	1998			1	1	1							3			1							1	4
		7	22	56	18	21	2	4	4	1	1	5	60	9	5	18	21	14	4	2	2	2	32	60

% of documents that mention item: 90% 55 | 75% 46 | 50% 31 | 25% 15

* items grouped as they were mentioned in only one document

First author	Year	Maize products: Unespecific	Drinks (Atole)	Tortillas (incl totopoxtle)	Soups (pozole, menudo)	Tamales (incl. zacahuil)	Cob (incl. esquites)	Pinole	Snacks with vegetables (tlacoyos, chilaquiles)	Others: tacos, popcorn, sopes, pellizcadas, gorditas, tostadas, peneques, totopos)	Total mentions
Aguirre-Beltran	1994			1							1
Allen	1992		1	1	1	1					4
Almaguer Gonzalez	2018		1	1	1	1				1	5
Algert	1998										0
Anderson	1946			1							1
Avila-Nava	2017										0
Barros	1999		1								1
Beals	1943										0
Berdan	2017		1								1
Bertran-Vila	2010			1							1
Bertrán	2005		1	1							2
Bertrán	2006		1	1							2
Burgos-Monzon	2013			1							1
Carrera	2007			1						1	2
Casillas	1984		1	1	1	1	1			1	6
Castelló Yturbide	1986		1	1		1		1		1	5
Cook	1980		1	1		1					3
Crocker Sagastume	2004										0
Davalos Hurtado	1994		1	1	1	1	1	1			6
Flores	2010		1	1		1				1	4
Flores y Escalante	2004		1	1	1	1	1	1	1		7
García Chávez	2017	1	1	1						1	4
Garcia Uriguen	2012		1	1		1					3
Harris	2004	1		1							2
Katz	1990										0
Kittler	2007		1	1		1			1	1	5
Llamas	1935		1	1		1					3
Long-Solis	2005		1	1		1					3
Lopez Alonso	1974		1	1	1	1	1	1			6
Márquez-Morfín	1991		1	1							2
McMurry	1991			1				1			2
Méndez y Mercado	1993										0
Mercado	2012		1	1			1		1		4
Moreno-Altamirano	2017										0
Murtaugh	2008										0
Ojeda-Granados	2017										0
Ortiz de Montellano	1990										0
Quevedo	2004					1				1	2
Quiñones Tapia	2019		1	1	1	1		1		1	6
Quiroz	2004		1	1		1					3
Ravussin	1994			1							1
Rendon	1947		1	1		1	1			1	5
Robles-Ordaz	2017										0
Rodríguez Morán	2009			1							1
Roman et al	2013										0
Romero-Gwynn	1994										0
Santiago-Torres	2015			1		1					2
Santiago-Torres	2016	1		1	1	1					4
Santley et al.	1979			1							1
Shamosh	2014		1	1		1		1		1	5
Soustelle	1970		1			1				1	3
Tseng	1997										0
UNESCO	2010			1		1					2
Vargas	1984			1		1					2
Vargas	1988		1	1		1					3
Vargas	2003		1	1	1	1	1	1		1	7
Velasco	1995		1	1		1		1			4
Weitlaner	1952		1	1		1		1			4
Wentworth	1936										0
Wicke	1959		1	1							2
Wyatt	1998			1							1
		3	30	42	9	27	7	10	3	13	46

% of documents that mention item: 90% 55 | 75% 46 | 50% 31 | 25% 15

First author	Year	Legumes: Unespecific	Beans	Lentils	Chickpeas	Broad beans	Peas	*Others: Ibis, soybeans, canigwa, tarwi, molle	Total mentions
Aguirre-Beltran	1994		1						1
Allen	1992	1	1					1	3
Almaguer Gonzalez	2018		1			1		1	3
Algert	1998		1						1
Anderson	1946		1						1
Avila-Nava	2017		1						1
Barros	1999		1						1
Beals	1943		1						1
Berdan	2017		1						1
Bertran-Vila	2010		1						1
Bertrán	2005		1	1		1		1	4
Bertrán	2006		1						1
Burgos-Monzon	2013		1	1			1	1	4
Carrera	2007		1					1	2
Casillas	1984		1						1
Castelló Yturbide	1986		1						1
Cook	1980		1						1
Crocker Sagastume	2004		1						1
Davalos Hurtado	1994		1					1	2
Flores	2010		1		1			1	3
Flores y Escalante	2004		1	1		1	1	1	5
García Chávez	2017	1							1
Garcia Uriguen	2012	1	1						2
Harris	2004		1						1
Katz	1990		1						1
Kittler	2007		1		1		1	1	4
Llamas	1935	1	1						2
Long-Solis	2005		1						1
Lopez Alonso	1974		1						1
Márquez-Morfín	1991	1	1						2
McMurry	1991		1						1
Méndez y Mercado	1993		1						1
Mercado	2012		1						1
Moreno-Altamirano	2017	1							1
Murtaugh	2008	1							1
Ojeda-Granados	2017		1						1
Ortiz de Montellano	1990		1						1
Quevedo	2004		1						1
Quiñones Tapia	2019		1						1
Quiroz	2004	1	1		1	1		1	5
Ravussin	1994		1						1
Rendon	1947		1		1			1	3
Robles-Ordaz	2017								0
Rodríguez Morán	2009		1						1
Roman et al	2013		1						1
Romero-Gwynn	1994		1						1
Santiago-Torres	2015		1						1
Santiago-Torres	2016		1						1
Santley et al.	1979		1						1
Shamosh	2014	1	1	1	1		1	1	6
Soustelle	1970	1	1						2
Tseng	1997		1						1
UNESCO	2010		1						1
Vargas	1984	1	1						2
Vargas	1988		1						1
Vargas	2003		1						1
Velasco	1995		1						1
Weitlaner	1952		1						1
Wentworth	1936		1						1
Wicke	1959		1						1
Wyatt	1998		1						1
		11	57	4	5	4	4	12	60

% of documents that mention item: 90% 55 | 75% 46 | 50% 31 | 25% 15

* items grouped as they were mentioned in only one document

		Vegetables																						
						Cactus																		
First author	Year	Unespecific	Squash	Chayote	Chilacayote	Unspecific	Nopales/cactus	Biznagas	Peyote	Maguey/agave	Tomato/ red tomato	Green tomato/ tomatillo	Other tomatos: Miltomate, costomate, citlali tomato	Auberigne	Radish	Bell peppers	Carrot	Cabbage	Beetroot	Cauliflower	Cucumber	Artichoke	Others*: celery,, asparagus, quiote, parsnip, turnip, caper, brocoli	Total mentions
Aguirre-Beltran	1994	1	1															1						3
Allen	1992		1				1				1				1								1	4
Almaguer Gonzalez	2018		1	1	1		1				1	1	1			1								8
Algert	1998		1				1			1														3
Anderson	1946					1	1				1	1												4
Avila-Nava	2017						1				1													2
Barros	1999		1	1							1													3
Beals	1943																	1						1
Berdan	2017	1	1																					2
Bertran-Vila	2010	1																						1
Bertrán	2005		1	1			1				1	1			1		1							7
Bertrán	2006		1								1	1												3
Burgos-Monzon	2013																							0
Carrera	2007	1																						1
Casillas	1984		1			1	1			1	1	1	1											7
Castelló Yturbide	1986		1	1			1				1	1												5
Cook	1980	1	1				1			1	1				1		1	1						8
Crocker Sagastume	2004		1				1		1		1													4
Davalos Hurtado	1994		1	1	1		1			1	1	1	1											8
Flores	2010		1	1			1										1	1		1	1		1	7
Flores y Escalante	2004		1	1	1	1	1	1			1	1	1	1	1	1	1	1	1		1	1	1	17
García Chávez	2017	1																						1
Garcia Uriguen	2012		1				1				1													3
Harris	2004	1																						1
Katz	1990		1	1			1					1												4
Kittler	2007		1	1			1				1	1												5
Llamas	1935										1													1
Long-Solis	2005		1	1			1				1	1												5
Lopez Alonso	1974		1	1			1				1													4
Márquez-Morfín	1991			1							1													2
McMurry	1991	1																						1
Méndez y Mercado	1993		1				1																	2
Mercado	2012	1																						1
Moreno-Altamirano	2017	1																						1
Murtaugh	2008										1													1
Ojeda-Granados	2017	1	1								1													3
Ortiz de Montellano	1990		1							1	1													3
Quevedo	2004		1	1	1		1			1	1													6
Quiñones Tapia	2019		1				1		1		1													4
Quiroz	2004										1				1	1	1		1	1		1	1	7
Ravussin	1994		1								1							1						3
Rendon	1947		1	1	1					1	1						1	1						7
Robles-Ordaz	2017	1																						1
Rodríguez Morán	2009																							0
Roman et al	2013		1				1	1			1	1												5
Romero-Gwynn	1994		1								1													2
Santiago-Torres	2015	1																						1
Santiago-Torres	2016		1				1																	2
Santley et al.	1979		1	1			1			1	1													5
Shamosh	2014		1	1	1		1	1			1	1	1	1			1						1	10
Soustelle	1970										1													1
Tseng	1997																							0
UNESCO	2010		1								1													2
Vargas	1984		1																					1
Vargas	1988		1				1			1	1	1												5
Vargas	2003		1	1						1	1	1												5
Velasco	1995		1					1		1	1	1												5
Weitlaner	1952		1	1							1	1												4
Wentworth	1936																							0
Wicke	1959		1	1						1	1	1												5
Wyatt	1998										1													1
		13	39	19	6	3	26	4	2	12	39	18	5	2	5	3	7	7	2	2	2	2	5	57
% of documents that mention item		90%	55	75%	46	50%	31	25%	15															

* items grouped as they were mentioned in only one document

First author	Year	Vegetables (cont.)																					
		Greens/wild leafy plants																					
		Unespecific	Chaya	Others*: Chenopods, endivia, turnip leaves, huizache, mastuerzo, palmito	Green beans	Guaje	Huauzontle	Lettuce	Malva/mallow	Mezquite	Papaloquelite or papalo (incl. pipicha)	Purslane	Quelites (including jabonera, tequelite, nonaquilitl)	Quintoniles	Romeritos	Setaria grass	Spinach	Squash leaves	Watercress	Xoconostle	Wild rice	Others*: ahuchua, tzonpenciu, yzmiquilitl, achuacatzin, chiyitos, hierba mora, xocoyule, tules, giant reed, nymph, hueynacaxtle, choco, pipicha, pipizca	Total mentions (greens)
Aguirre-Beltran	1994	1							1														2
Allen	1992																						0
Almaguer Gonzalez	2018		1		1		1					1	1	1	1				1	1			9
Algert	1998	1																					1
Anderson	1946			1					1			1	1										4
Avila-Nava	2017																						0
Barros	1999						1						1										2
Beals	1943	1																					1
Berdan	2017																						0
Bertran-Vila	2010																						0
Bertrán	2005		1	1								1	1	1									5
Bertrán	2006												1					1					2
Burgos-Monzon	2013																						0
Carrera	2007																						0
Casillas	1984						1			1			1	1		1							5
Castelló Yturbide	1986					1	1			1	1	1	1	1									7
Cook	1980	1						1															2
Crocker Sagastume	2004					1							1										2
Davalos Hurtado	1994						1		1	1			1	1						1			6
Flores	2010	1			1			1															3
Flores y Escalante	2004					1		1	1		1	1	1				1		1			1	9
García Chávez	2017																						0
Garcia Uriguen	2012					1						1											2
Harris	2004																						0
Katz	1990					1				1	1		1	1		1						1	7
Kittler	2007	1						1															2
Llamas	1935																					1	1
Long-Solis	2005																						0
Lopez Alonso	1974	1					1		1	1			1	1									6
Márquez-Morfín	1991		1																				1
McMurry	1991																						0
Méndez y Mercado	1993			1								1	1										3
Mercado	2012																						0
Moreno-Altamirano	2017																						0
Murtaugh	2008																						0
Ojeda-Granados	2017												1										1
Ortiz de Montellano	1990									1													1
Quevedo	2004					1					1	1	1	1								1	6
Quiñones Tapia	2019					1						1	1										3
Quiroz	2004	1						1															2
Ravussin	1994																						0
Rendon	1947									1			1									1	3
Robles-Ordaz	2017																						0
Rodríguez Morán	2009	1																					1
Roman et al	2013									1			1										2
Romero-Gwynn	1994												1										1
Santiago-Torres	2015																						0
Santiago-Torres	2016																						0
Santley et al.	1979			1								1				1					1		4
Shamosh	2014		1		1	1	1	1		1	1	1	1	1						1		1	12
Soustelle	1970												1										1
Tseng	1997																						0
UNESCO	2010																						0
Vargas	1984		1													1							2
Vargas	1988						1						1			1							3
Vargas	2003	1	1				1			1	1		1	1	1	1							9
Velasco	1995						1				1		1									1	4
Weitlaner	1952												1										1
Wentworth	1936																						0
Wicke	1959																						0
Wyatt	1998							1															1
		10	6	4	3	8	10	7	5	10	7	11	25	10	2	6	1	1	2	3	1	7	40

% of documents that mention item: 90% 55 | 75% 46 | 50% 31 | 25% 15

* items grouped as they were mentioned in only one document

First author	Year	**Vegetables** (cont.) **Fungi**: Mushrooms	Huitlacoche	Others: anacate, añil	Total mentions (fungi)	**Blossoms**: Unespecific	Squash blossoms	Colorin blossoms	Flor de izote	Cacomite	Yucca blossoms	Maguey blossoms	Turnip/ nabo blossoms	Biznaga	Flor de mayo	Others: teonacaztli, tecomaxochitl	Garambullo	Total mentions (blossoms)	**Algae**: Others: amomoxtle, capulin, cuculito de agua	Spirulina or tecuitlatl	Total mentions (algae)	**Total mentions (all veg)**
Aguirre-Beltran	1994				0													0			0	5
Allen	1992				0													0			0	4
Almaguer Gonzalez	2018	1	1		2			1	1									2			0	21
Algert	1998				0													0			0	4
Anderson	1946				0						1	1	1				1	4			0	12
Avila-Nava	2017				0													0			0	2
Barros	1999				0													0		1	1	6
Beals	1943				0													0			0	2
Berdan	2017				0													0			0	2
Bertran-Vila	2010				0													0			0	1
Bertrán	2005	1			1													0			0	13
Bertrán	2006				0													0			0	5
Burgos-Monzon	2013				0													0			0	0
Carrera	2007				0													0				1
Casillas	1984				0													0		1	1	13
Castelló Yturbide	1986	1	1	1	3		1	1	1					1	1			5	1	1	2	22
Cook	1980				0													0			0	10
Crocker Sagastume	2004	1		1	2													0			0	8
Davalos Hurtado	1994	1			1					1								1		1	0	16
Flores	2010				0													0			0	10
Flores y Escalante	2004	1	1		2		1	1			1							3			0	31
García Chávez	2017				0													0			0	1
Garcia Uriguen	2012				0													0			0	5
Harris	2004				0													0			0	1
Katz	1990	1			1													0			0	12
Kittler	2007				0		1											1			0	8
Llamas	1935	1			1													0		1	1	4
Long-Solis	2005		1		1		1											1			0	7
Lopez Alonso	1974	1			1											1		1			0	12
Márquez-Morfín	1991				0													0			0	3
McMurry	1991				0													0			0	1
Méndez y Mercado	1993				0													0			0	5
Mercado	2012				0													0			0	1
Moreno-Altamirano	2017				0													0			0	1
Murtaugh	2008				0													0			0	1
Ojeda-Granados	2017				0													0	1		1	5
Ortiz de Montellano	1990				0													0		1	1	5
Quevedo	2004	1	1		2													0		1	1	15
Quiñones Tapia	2019	1			1													0			1	9
Quiroz	2004				0													0			0	9
Ravussin	1994				0													0			0	3
Rendon	1947				0													0			0	10
Robles-Ordaz	2017				0													0			0	1
Rodríguez Morán	2009				0													0			0	1
Roman et al	2013				0													0			0	7
Romero-Gwynn	1994				0													0			0	3
Santiago-Torres	2015				0													0			0	1
Santiago-Torres	2016				0													0			0	2
Santley et al.	1979				0													0		1	1	10
Shamosh	2014	1	1		2	1	1	1		1	1	1		1	1	1	1	10	1	1	2	36
Soustelle	1970				0													0		1	1	3
Tseng	1997				0													0			0	0
UNESCO	2010				0													0			0	2
Vargas	1984				0													0			0	3
Vargas	1988				0													0		1	1	9
Vargas	2003		1		1							1						1		1	1	17
Velasco	1995				0	1	1	1	1									4		1	1	14
Weitlaner	1952	1			1													0			0	6
Wentworth	1936				0													0			0	0
Wicke	1959	1	1		2													0	1		1	8
Wyatt	1998				0													0			0	2
		14	8	2	16	2	6	5	3	2	3	3	1	2	2	2	2	11	4	13	15	58

% of documents that mention item	90%	55	75%	46	50%	31	25%	15

* items grouped as they were mentioned in only one document

First author	Year	Fruits																						
		Unspecified	Anona/ chirimoya /custard apple/ sweet sop	Apple	Apricots	Banana	Berries (all types)	Capulin	Others: caimito, carambola, casimiroa, cosahuico,	Cherries	Chicozapote	Citrus fruits	Coconut	Coyol	Cuajilote	Cuajinicuil/ juakinicuil	Cuapinole	Fig	Grapes	Grapefruit	Guamuchil or huamuchil	Guava (inc. pomarrosa)	Guanabana and soursop	Jícama
Aguirre-Beltran	1994	1																						
Allen	1992			1	1	1						1						1						1
Almaguer Gonzalez	2018	1	1				1	1			1											1	1	1
Algert	1998																					1		
Anderson	1946																							
Avila-Nava	2017																							
Barros	1999							1														1		1
Beals	1943					1						1												
Berdan	2017																							
Bertran-Vila	2010	1																						
Bertrán	2005			1		1						1												
Bertrán	2006	1										1												
Burgos-Monzon	2013																							
Carrera	2007	1										1												
Casillas	1984							1	1													1		
Castelló Yturbide	1986		1					1	1		1		1									1	1	1
Cook	1980	1		1	1	1	1					1										1		
Crocker Sagastume	2004																							
Davalos Hurtado	1994		1					1							1	1	1					1	1	1
Flores	2010			1		1	1					1								1		1		
Flores y Escalante	2004		1	1			1	1		1		1		1				1	1			1	1	1
García Chávez	2017	1																						
Garcia Uriguen	2012							1																
Harris	2004	1																						
Katz	1990		1					1						1								1		
Kittler	2007		1			1	1		1			1	1									1	1	1
Llamas	1935																							
Long-Solis	2005		1	1		1	1			1		1							1	1		1	1	1
Lopez Alonso	1974		1					1														1	1	1
Márquez-Morfín	1991		1			1						1											1	1
McMurry	1991	1																						
Méndez y Mercado	1993			1														1						
Mercado	2012	1																						
Moreno-Altamirano	2017	1																						
Murtaugh	2008																							
Ojeda-Granados	2017																							
Ortiz de Montellano	1990																							
Quevedo	2004		1					1			1		1		1						1	1	1	1
Quiñones Tapia	2019																				1			
Quiroz	2004			1	1	1				1		1	1					1						
Ravussin	1994			1		1						1												
Rendon	1947		1				1			1		1												
Robles-Ordaz	2017	1																						
Rodríguez Morán	2009	1																						
Roman et al	2013	1																						
Romero-Gwynn	1994		1									1											1	1
Santiago-Torres	2015	1																						
Santiago-Torres	2016											1												1
Santley et al.	1979							1																
Shamosh	2014		1			1	1	1	1	1		1	1		1		1	1	1	1	1	1	1	1
Soustelle	1970																							
Tseng	1997																							
UNESCO	2010																							
Vargas	1984	1																			1	1		1
Vargas	1988		1					1														1		
Vargas	2003							1																
Velasco	1995		1					1														1		1
Weitlaner	1952		1			1		1			1	1	1	1					1	1		1	1	1
Wentworth	1936																							
Wicke	1959						1	1														1		1
Wyatt	1998					1						1												
		16	16	9	3	13	9	17	4	5	4	19	6	3	3	1	2	5	4	4	4	20	12	18

% of documents that mention item	90%	55	75%	46	50%	31	25%	15

* items grouped as they were mentioned in only one document

First author	Year	Fruits (cont.)																					
		Mamey	Mango	Others: Matasano, níspero, parota, passion fruit, pickle	Melon	Nance/nanche/huachacote	Orange	Papaya	Pear	Peach	Pineapple	Pitahaya	Pitaya	Plantains	Plums/xocotl/hobo/jobo, icaco, jocote)	Quince	Ramon	Tamarind	Tejocote	Prickly pear	Others*: pomegranate, guamocotl, raisins, mandarin, texalcapotl, uste, watermelon	Zapote	Total mentions (all fruits)
Aguirre-Beltran	1994																				1		4
Allen	1992				1					1	1				1								22
Almaguer Gonzalez	2018	1				1		1			1	1			1				1	1		1	33
Algert	1998																						3
Anderson	1946																			1			1
Avila-Nava	2017																						0
Barros	1999														1							1	11
Beals	1943						1		1														8
Berdan	2017																						0
Bertran-Vila	2010																						3
Bertrán	2005		1							1													11
Bertrán	2006																						6
Burgos-Monzon	2013																						0
Carrera	2007													1									7
Casillas	1984	1				1					1				1					1		1	15
Castelló Yturbide	1986	1				1		1							1				1	1		1	31
Cook	1980						1			1						1				1			25
Crocker Sagastume	2004																			1			1
Davalos Hurtado	1994	1				1		1			1	1			1				1	1		1	33
Flores	2010		1		1		1	1	1		1										1		25
Flores y Escalante	2004	1		1				1	1		1				1			1	1		1	1	46
García Chávez	2017																						3
Garcia Uriguen	2012														1				1			1	6
Harris	2004																						3
Katz	1990	1				1		1							1				1	1	1	1	20
Kittler	2007	1	1	1	1		1	1			1			1						1		1	37
Llamas	1935																						0
Long-Solis	2005		1		1		1	1	1	1	1	1		1	1					1	1	1	46
Lopez Alonso	1974	1				1					1	1			1				1	1		1	23
Márquez-Morfín	1991	1				1											1					1	19
McMurry	1991																						3
Méndez y Mercado	1993	1							1						1					1	1	1	12
Mercado	2012																						3
Moreno-Altamirano	2017																						3
Murtaugh	2008																						0
Ojeda-Granados	2017																						0
Ortiz de Montellano	1990																						0
Quevedo	2004	1		1		1		1							1			1	1	1		1	36
Quiñones Tapia	2019												1									1	5
Quiroz	2004	1					1		1		1				1	1			1		1	1	30
Ravussin	1994		1				1			1													12
Rendon	1947											1						1		1		1	16
Robles-Ordaz	2017																						3
Rodríguez Morán	2009																			1			4
Roman et al	2013																			1			4
Romero-Gwynn	1994	1						1					1							1		1	17
Santiago-Torres	2015																						3
Santiago-Torres	2016																						6
Santley et al.	1979																		1	1		1	6
Shamosh	2014	1	1	1	1	1	1	1	1	1	1	1	1		1		1	1	1	1		1	69
Soustelle	1970																						0
Tseng	1997																						0
UNESCO	2010																						0
Vargas	1984	1		1				1							1		1					1	18
Vargas	1988	1				1					1	1			1					1		1	16
Vargas	2003														1		1		1	1			7
Velasco	1995														1				1	1		1	16
Weitlaner	1952	1	1				1	1					1		1			1					43
Wentworth	1936																						0
Wicke	1959										1								1	1		1	16
Wyatt	1998						1																7
		17	7	5	5	10	10	13	7	6	13	7	4	3	20	2	4	5	14	23	7	24	50

% of documents that mention item								
90%	55	75%	46	50%	31	25%	15	

* items grouped as they were mentioned in only one document

First author	Year	Oils and fats									Nuts and seeds											
		Unespecific	Vegetable oil	Processed fats (margarine/ mayonaise)	Avocado	Butter	Cream and dressings	Lard and animal fats	Olives	**Total mentions**	Unespecific	Peanuts	Pumpkin seeds	Chia seeds	Pine nuts	Walnuts	Acorns	Almonds	Sesame seeds	Ohers*: flax, amaranth, Brazil nuts, chestnuts, cashew, nut pastes, sunflower, cotton, susi	Mezquite seeds	**Total mentions**
Aguirre-Beltran	1994									0												0
Allen	1992	1	1		1					2		1										1
Almaguer Gonzalez	2018				1					1		1	1	1	1							4
Algert	1998				1					1												0
Anderson	1946							1		1												0
Avila-Nava	2017									0			1	1								2
Barros	1999									0				1								1
Beals	1943									0												0
Berdan	2017									0												0
Bertran-Vila	2010									0												0
Bertrán	2005				1					1												0
Bertrán	2006				1			1		2												0
Burgos-Monzon	2013									0												0
Carrera	2007	1	1	1			1			3	1	1										2
Casillas	1984				1					1		1	1									2
Castelló Yturbide	1986				1					1		1										1
Cook	1980									0		1		1		1						3
Crocker Sagastume	2004		1					1		2												0
Davalos Hurtado	1994				1					1		1	1	1								3
Flores	2010			1	1	1	1			4	1											1
Flores y Escalante	2004				1				1	2		1	1	1		1			1	1		6
García Chávez	2017		1	1	1		1			4	1	1										2
Garcia Uriguen	2012				1					1												0
Harris	2004							1		1												0
Katz	1990				1					1	1				1		1			1		4
Kittler	2007				1	1		1		3			1		1				1	1		4
Llamas	1935									0				1								1
Long-Solis	2005				1	1	1	1		4			1						1			2
Lopez Alonso	1974									0		1	1				1					3
Márquez-Morfín	1991									0												0
McMurry	1991									0												0
Méndez y Mercado	1993				1					1			1	1								2
Mercado	2012		1					1		2												0
Moreno-Altamirano	2017		1							1												0
Murtaugh	2008									0												0
Ojeda-Granados	2017									0				1								1
Ortiz de Montellano	1990									0												0
Quevedo	2004									0		1	1	1					1			4
Quiñones Tapia	2019									0			1									1
Quiroz	2004							1		1					1			1	1			3
Ravussin	1994				1					1												0
Rendon	1947				1					1							1					1
Robles-Ordaz	2017									0												0
Rodríguez Morán	2009									0												0
Roman et al	2013				1					1			1	1								2
Romero-Gwynn	1994								1	1								1				1
Santiago-Torres	2015	1	1							1												0
Santiago-Torres	2016							1		1												0
Santley et al.	1979				1					1				1								1
Shamosh	2014		1		1		1			3		1	1	1	1				1	1	1	7
Soustelle	1970				1					1	1											1
Tseng	1997									0												0
UNESCO	2010				1					1												0
Vargas	1984				1			1		2					1							1
Vargas	1988				1					1		1		1								2
Vargas	2003				1					1			1	1							1	3
Velasco	1995				1					1			1	1								2
Weitlaner	1952				1			1		2			1					1		1		3
Wentworth	1936									0												0
Wicke	1959				1					1			1							1		2
Wyatt	1998									0												0
		3	8	3	29	3	5	11	2	39	5	13	17	16	6	2	3	3	6	6	2	34

% of documents that mention item	90%	55	75%	46	50%	31	25%	15

* items grouped as they were mentioned in only one document

First author	Year	Beverages																														
		Alcoholic beverages																Non-alcoholic beverages														
		Unespecific	Pulque	Mezcal	Tesguino	Tejuino	Maguey	Chicha	Tuba	Beer	Tequila	Wine	Chiringuito or aguardiente	Others*: balch'e, taberna, mate, guayusa, whiskey, charagua, charape, sototl, piznatem chorote, popo, tazcalate, tejate, mejengue, guasimo, chilacayota, huikimo, tuba, blossom liquors, bote, verde de xico,	From seeds: maize, amaranth, chia, cacao	Colonche	Tepache	Plain water	Maguey juice/ nectar	Maize	Chia	Bledos/ amaranth	Seeds	Coffee	Cocoa or cacao	Soda	Tea	Natura	Natural fruit drinks/ aguas frescas	Sports drinks	Industrialised drinks	Total menitons
Aguirre-Beltran	1994		1																						1							2
Allen	1992		1									1								1			1		1							5
Almaguer Gonzalez	2018				1	1												1	1						1							5
Algert	1998																															0
Anderson	1946		1																													1
Avila-Nava	2017																															0
Barros	1999																								1							1
Beals	1943																															0
Berdan	2017		1																						1							2
Bertran-Vila	2010																															0
Bertrán	2005		1	1	1									1				1						1	1	1		1	1		1	11
Bertrán	2006																											1	1			2
Burgos-Monzon	2013																									1						1
Carrera	2007	1																						1			1		1		1	5
Casillas	1984																			1					1							2
Castelló Yturbide	1986		1		1		1	1	1					1	1	1	1			1	1	1			1							13
Cook	1980		1																													1
Crocker Sagastume	2004																															0
Davalos Hurtado	1994		1				1								1				1	1	1	1			1							8
Flores	2010	1																						1		1	1	1	1			6
Flores y Escalante	2004		1		1			1	1					1	1										1							7
García Chávez	2017																							1			1	1	1	1	1	6
Garcia Uriguen	2012																								1							1
Harris	2004																															0
Katz	1990		1																1						1							3
Kittler	2007		1	1						1	1	1		1										1	1				1			9
Llamas	1935		1				1																		1							3
Long-Solis	2005												1												1						1	3
Lopez Alonso	1974		1												1					1	1	1			1							6
Márquez-Morfín	1991																															0
McMurry	1991																							1								1
Méndez y Mercado	1993																								1							1
Mercado	2012																							1	1		1		1			4
Moreno-Altamirano	2017																															0
Murtaugh	2008																															0
Ojeda-Granados	2017																															0
Ortiz de Montellano	1990		1																													1
Quevedo	2004		1									1													1							3
Quiñones Tapia	2019					1																										1
Quiroz	2004		1										1												1							3
Ravussin	1994									1	1													1		1						4
Rendon	1947			1									1				1	1	1					1		1	1					8
Robles-Ordaz	2017																															0
Rodríguez Morán	2009																															0
Roman et al	2013																															0
Romero-Gwynn	1994																								1							1
Santiago-Torres	2015																															0
Santiago-Torres	2016																												1			1
Santley et al.	1979																															0
Shamosh	2014		1		1	1		1	1			1			1	1		1						1	1		1					12
Soustelle	1970		1															1														3
Tseng	1997																															0
UNESCO	2010																								1							1
Vargas	1984													1	1										1							3
Vargas	1988		1															1							1							3
Vargas	2003		1	1		1													1						1							5
Velasco	1995																								1							1
Weitlaner	1952											1						1						1	1							4
Wentworth	1936																							1								1
Wicke	1959		1																						1							2
Wyatt	1998									1														1		1						3
		2	21	4	5	4	3	3	3	3	2	5	3	5	6	2	2	7	5	5	3	3	1	13	30	6	6	4	8	1	4	45

% of documents that mention item

90%	55	75%	46	50%	31	25%	15

* items grouped as they were mentioned in only one document

		Fish and seafood																									
		Fish																Seafood									
First author	Year	Unespecific	Catfish (bagre)	Devil ray (mantarraya)	Juiles/ cuiles	Sardines	Saw fish	Sea bass/ snook (robalo)	Shark/ cazon	Mojarra	Trout	Sole (lenguado)	Mullet/ bobo mullet and grey mullet (lisa)	Whitefish	Charales	Snapper (besugo, guauchinango, pargos)	Others*: carp, tuna, pampanos, congers (congrios) sturgeon (sollos), mextlapique	Others*: xalmichi, xohuili, pescadillos de arena, cuitlapetlate, michcahuon, michin, tlacamichin, pigfish, caballos, pepesca, jolote, boabino, roncador	Unespecific	Crayfish (acamaya)	Crab (incl. Jaibas)	Others*: eels, lobster	Shrimp /prawn	Freswater shrimp (acociles)	Shellfish (including mussels and oysters)	Octopi	Snails	Total mentions (fish and seafood)
Aguirre-Beltran	1994																											0
Allen	1992																											0
Almaguer Gonzalez	2018		1	1			1	1	1	1	1			1					1	1	1		1		1	1		14
Algert	1998																											0
Anderson	1946																											0
Avila-Nava	2017	1																										1
Barros	1999	1																	1									2
Beals	1943	1																										1
Berdan	2017	1																	1									2
Bertran-Vila	2010																											0
Bertrán	2005	1				1											1						1				1	5
Bertrán	2006	1																										1
Burgos-Monzon	2013																											0
Carrera	2007	1																	1									2
Casillas	1984	1																	1					1				3
Castelló Yturbide	1986		1						1								1						1	1				5
Cook	1980	1																		1	1		1	1				5
Crocker Sagastume	2004																											0
Davalos Hurtado	1994															1	1											2
Flores	2010	1				1											1				1		1		1	1		7
Flores y Escalante	2004																							1				1
García Chávez	2017	1																	1									2
Garcia Uriguen	2012	1																										1
Harris	2004	1																							1			2
Katz	1990																											0
Kittler	2007															1							1					2
Llamas	1935				1									1				1						1				4
Long-Solis	2005																											0
Lopez Alonso	1974																	1			1		1	1				4
Márquez-Morfín	1991		1	1			1	1		1	1	1	1					1				1	1		1	1	1	14
McMurry	1991																											0
Méndez y Mercado	1993																											0
Mercado	2012	1																										1
Moreno-Altamirano	2017																											0
Murtaugh	2008																											0
Ojeda-Granados	2017																											0
Ortiz de Montellano	1990	1													1													2
Quevedo	2004	1	1											1	1						1		1	1	1			8
Quiñones Tapia	2019	1																										1
Quiroz	2004	1																										1
Ravussin	1994																											0
Rendon	1947	1													1						1							3
Robles-Ordaz	2017	1																	1									2
Rodríguez Morán	2009																											0
Roman et al	2013	1																	1									2
Romero-Gwynn	1994	1																										1
Santiago-Torres	2015																											0
Santiago-Torres	2016																											0
Santley et al.	1979	1																										1
Shamosh	2014		1											1				1					1	1				5
Soustelle	1970	1																			1			1	1			4
Tseng	1997																											0
UNESCO	2010																											0
Vargas	1984	1		1		1		1		1		1	1												1	1		9
Vargas	1988	1																	1				1					3
Vargas	2003	1																	1									2
Velasco	1995				1									1	1									1				4
Weitlaner	1952		1		1			1		1	1		1					1			1		1				1	10
Wentworth	1936																											0
Wicke	1959	1																				1	1		1		1	5
Wyatt	1998	1																										1
		29	6	3	3	3	2	4	2	4	3	2	3	5	4	2	4	5	10	2	8	2	13	10	8	4	4	40

% of documents that mention item

90%	55	75%	46	50%	31	25%	15

* items grouped as they were mentioned in only one document

First author	Year	Poultry and mammals<br>Meats and meat dishes	Poultry<br>Unespecific	Waterfowl (incl. xalquani, pipitztli, quetzaltecoloton)	Turkey	Chicken/ hen	Quail	Pheasant	Partridges	Ducks (including mergansers, ánades, Metzacanauhtli, ehecatótotl, amanacoche, yacatextli, zolcanauhtl, chilcanauhtli, yacapatláhuac)	Geese (including ánsares)	Parrots	Chachalacas	Pigeon (including huilotas)	Doves	Plover (chichicuilote/tzitzicuilotl)	*Others: Gacetas, ibis, avetoros, martinetes, somorujos, zampollines, janacas, trinngas, falaropos, numénidos, pelican (atotolin), Chichicaxtle, zacacintli,, acitlin, tenitzli, couxin, quetzaltecoloton, guacoxtli, tzitzuia, nacaztzona	Heron (including Aztatl, axoque)	Crows	Cranes, rail and coots	Hawk and eagles/(cauauhtli	Total mentions (poultry)
Aguirre-Beltran	1994																					0
Allen	1992			1		1																2
Almaguer Gonzalez	2018				1	1																2
Algert	1998																					0
Anderson	1946		1																			1
Avila-Nava	2017		1		1																	2
Barros	1999		1		1		1															3
Beals	1943																					0
Berdan	2017				1																	1
Bertran-Vila	2010					1																1
Bertrán	2005	1				1																2
Bertrán	2006	1				1																2
Burgos-Monzon	2013																					0
Carrera	2007				1	1																2
Casillas	1984				1					1			1			1	1					5
Castelló Yturbide	1986				1					1		1				1						4
Cook	1980		1		1	1				1												4
Crocker Sagastume	2004																					0
Davalos Hurtado	1994				1		1	1	1	1			1	1	1					1		9
Flores	2010					1																1
Flores y Escalante	2004				1			1	1	1				1		1	1					7
García Chávez	2017	1																				0
Garcia Uriguen	2012		1		1	1																3
Harris	2004		1																			1
Katz	1990				1		1							1	1							4
Kittler	2007				1	1																2
Llamas	1935		1				1			1						1		1		1	1	7
Long-Solis	2005				1	1																2
Lopez Alonso	1974		1		1		1			1				1			1			1		7
Márquez-Morfín	1991				1	1		1	1	1			1									6
McMurry	1991																					0
Méndez y Mercado	1993																					0
Mercado	2012					1																1
Moreno-Altamirano	2017	1																				1
Murtaugh	2008	1																				1
Ojeda-Granados	2017																					0
Ortiz de Montellano	1990			1	1	1																3
Quevedo	2004				1		1	1	1	1	1			1		1						8
Quiñones Tapia	2019		1		1	1																3
Quiroz	2004				1	1																2
Ravussin	1994	1				1																2
Rendon	1947	1	1							1											1	4
Robles-Ordaz	2017																					0
Rodríguez Morán	2009	1																				1
Roman et al	2013				1																	1
Romero-Gwynn	1994		1		1	1																3
Santiago-Torres	2015																					0
Santiago-Torres	2016	1																				1
Santley et al.	1979			1	1																	2
Shamosh	2014				1	1	1	1	1	1		1		1	1	1						10
Soustelle	1970			1	1			1							1				1			5
Tseng	1997																					0
UNESCO	2010																					0
Vargas	1984		1																			1
Vargas	1988			1	1																	2
Vargas	2003		1		1				1	1												4
Velasco	1995			1	1					1						1	1	1		1		7
Weitlaner	1952				1				1	1		1	1	1								6
Wentworth	1936	1																				1
Wicke	1959				1		1	1	1	1										1		6
Wyatt	1998	1																				1
		11	13	6	30	19	8	7	8	15	1	3	4	7	4	7	4	2	1	5	2	48

% of documents that mention item: 90% 55 | 75% 46 | 50% 31 | 25% 15

* items grouped as they were mentioned in only one document

First author	Year	Mammals																																	Total mentions (poultry and mammals)
		Unespecific red meat (inc. blood and organs)	Processed meats (longaniza, moronga, queso de puerco, escabeches de pata, ham, chorizo, sausage, bacon)	Unespecific	Manatee	Venison	Pork	Tapir/antebурro	Rabbit	Hare	Weasel	Skunks	Otter	Badger	Beef	Lamb	Chevon	Dogs (including itzcuintli)	Tlacuache (opossum)	Cacomixtle/ racoon,	Coati	Marten	Bear	Paca (Tepezcuintle, tuza), pocket gophers	Armadillo	Coyote	Fox	Squirrel	Wild boar, peccari, hogs	Mole	Rats and mice	Tigrillo (wildcat)	Monkey	Total mentions (mammals)	
Aguirre-Beltran	1994											1			1			1	1												1			5	5
Allen	1992					1	1		1						1	1	1												1					7	9
Almaguer Gonzalez	2018					1	1		1						1	1	1																	6	8
Algert	1998																																	0	0
Anderson	1946	1							1							1	1																	4	5
Avila-Nava	2017																																	0	2
Barros	1999					1			1	1								1																4	7
Beals	1943														1																			1	1
Berdan	2017					1			1									1																3	4
Bertran-Vila	2010														1																			1	2
Bertrán	2005														1																			1	3
Bertrán	2006																																	0	2
Burgos-Monzon	2013																																	0	0
Carrera	2007	1	1				1								1	1																		5	7
Casillas	1984								1		1	1						1	1	1				1	1			1			1			10	15
Castelló Yturbide	1986					1			1									1	1					1	1			1	1				1	9	13
Cook	1980			1												1	1	1																4	8
Crocker Sagastume	2004																																	0	0
Davalos Hurtado	1994					1		1	1	1	1		1	1				1	1	1		1	1	1	1			1	1					16	25
Flores	2010	1	1				1								1																			4	5
Flores y Escalante	2004					1			1									1	1	1					1									6	13
García Chávez	2017		1																															1	1
Garcia Uriguen	2012			1		1	1			1					1			1																6	9
Harris	2004						1								1		1																	3	4
Katz	1990					1			1	1									1	1	1				1			1	1					9	13
Kittler	2007	1					1								1		1																	4	6
Llamas	1935					1			1	1																								3	10
Long-Solis	2005						1								1	1	1																	4	6
Lopez Alonso	1974					1			1			1						1	1				1							1				7	14
Márquez-Morfín	1991				1	1	1		1									1			1				1									7	13
McMurry	1991																																	0	0
Méndez y Mercado	1993								1	1																					1			3	3
Mercado	2012	1																																1	2
Moreno-Altamirano	2017																																	0	1
Murtaugh	2008																																	0	1
Ojeda-Granados	2017																																	0	0
Ortiz de Montellano	1990										1							1						1	1						1			5	8
Quevedo	2004				1	1	1	1	1	1	1	1	1					1	1			1	1	1	1	1			1				1	18	26
Quiñones Tapia	2019		1			1									1										1			1						5	8
Quiroz	2004		1				1								1		1																	4	6
Ravussin	1994																																	0	2
Rendon	1947	1		1		1			1			1							1					1	1		1	1	1		1			12	16
Robles-Ordaz	2017																																	0	0
Rodríguez Morán	2009																																	0	1
Roman et al	2013					1												1							1									3	4
Romero-Gwynn	1994					1									1		1												1				1	5	8
Santiago-Torres	2015		1																															1	1
Santiago-Torres	2016																																	0	1
Santley et al.	1979					1			1									1																3	5
Shamosh	2014					1	1		1	1		1			1	1	1	1	1	1				1	1			1	1	1	1		1	18	28
Soustelle	1970					1			1	1								1											1					5	10
Tseng	1997	1																																1	1
UNESCO	2010																																	0	0
Vargas	1984				1	1																												2	3
Vargas	1988				1	1			1	1								1	1					1	1				1					9	11
Vargas	2003					1	1		1	1								1						1	1				1					8	12
Velasco	1995					1			1	1	1							1	1	1				1	1			1			1			11	18
Weitlaner	1952	1					1	1	1					1	1					1				1	1			1				1		11	17
Wentworth	1936																																	0	1
Wicke	1959					1			1	1				1				1	1										1	1				8	14
Wyatt	1998																																	0	1
		8	6	3	4	25	14	3	24	13	5	6	2	3	17	7	10	21	13	7	2	2	3	11	16	1	1	9	12	3	7	1	4	45	54

% of documents that mention item								
	90%	55	75%	46	50%	31	25%	15

* items grouped as they were mentioned in only one document

First author	Year	Eggs					Dairy						Insects														Amphibians			
		Unespecific	Chicken and other poultry	Fish (inc. michpilli)	Reptile (iguana, turtle, snake)	Total mentions (eggs)	Unespecific	Cheese	Milk	Other: goat milk, evaporated milk	Yogurt and other milk products (jocoque)	**Total mentions**	Unespecific	Grasshoppers and locust	Maize worms	Maguey worms (chinicuiles/chilocuiles)	Ants (including chicatana, jicoteras and escamoles)	Bee and wasp larvae	Jumiles	Axacayacatl flyes	Water-fly or amayotl	Dragonfly and butterfly larvae (or aneneztli)	Eggs (ahuautle)	* Other insects: beetles, atetepitz, ticocos, cupiches, cuetla, stink bugs	* Other worms: jonote, guasimo, izcauitli, verde de la Mixteca, ocuiliztac, ahuihuitl	Total mentions	Unespecific	Frogs, toads, and tadpoles (including acacueyatl)	Ajolote or salamander	Total mentions
Aguirre-Beltran	1994					0						0	1	1		1	1		1	1			1		1	8		1		1
Allen	1992					0			1			1														0				0
Almaguer Gonzalez	2018		1			1		1				1		1		1	1		1	1						5				0
Algert	1998					0						0														0				0
Anderson	1946	1				1			1			1	1												1	2				0
Avila-Nava	2017					0						0	1													1				0
Barros	1999			1		1						0									1					1				0
Beals	1943					0		1	1			2														0				0
Berdan	2017					0						0	1				1									2				0
Bertran-Vila	2010	1				1		1	1			2														0				0
Bertrán	2005					0		1				1		1		1	1									3				0
Bertrán	2006	1				1		1				1	1													1				0
Burgos-Monzon	2013	1				1						0														0				0
Carrera	2007	1				1	1	1	1			3														0				0
Casillas	1984			1		1						0								1	1					2		1	1	2
Castelló Yturbide	1986					0						0		1		1	1		1	1	1		1	1		8		1	1	2
Cook	1980		1			1						0	1												1	2	1			0
Crocker Sagastume	2004	1				1						0														0				0
Davalos Hurtado	1994				1	1						0				1					1			1	1	4		1		1
Flores	2010	1				1		1	1		1	3														0				0
Flores y Escalante	2004			1		1						0		1												1		1	1	2
García Chávez	2017	1				1	1	1	1		1	4														0				0
Garcia Uriguen	2012					0						0														0				0
Harris	2004					0						0														0				0
Katz	1990					0						0		1	1		1									3		1		1
Kittler	2007		1			1		1	1	1		3														0				0
Llamas	1935					0						0					1			1	1		1	1		5		1	1	2
Long-Solis	2005					0		1				1														0				0
Lopez Alonso	1974		1		1	2						0		1			1		1	1	1	1	1		1	8		1		1
Márquez-Morfín	1991	1				1						0														0				0
McMurry	1991		1			1						0														0				0
Méndez y Mercado	1993					0						0														0				0
Mercado	2012					0			1		1	2														0				0
Moreno-Altamirano	2017					0			1			1														0				0
Murtaugh	2008					0		1				1														0				0
Ojeda-Granados	2017					0						0	1													1				0
Ortiz de Montellano	1990			1		1						0		1		1	1		1	1		1	1		1	8		1	1	2
Quevedo	2004					0						0		1		1	1		1			1			1	6		1		1
Quiñones Tapia	2019	1				1						0														0				
Quiroz	2004		1			1		1				1														0				0
Ravussin	1994	1				1			1			1														0				0
Rendon	1947	1				1		1			1	2					1								1	2				0
Robles-Ordaz	2017					0						0														0				0
Rodríguez Morán	2009					0			1			1														0				0
Roman et al	2013					0						0	1													1		1		1
Romero-Gwynn	1994					0	1					1	1													1		1		1
Santiago-Torres	2015					0		1	1			2														0				0
Santiago-Torres	2016					0			1			1														0				0
Santley et al.	1979					0						0														0				0
Shamosh	2014		1		1	2		1				1		1		1	1		1		1		1		1	7		1	1	2
Soustelle	1970					0						0				1	1				1	1	1			5		1	1	2
Tseng	1997					0			1			1														0				0
UNESCO	2010					0						0														0				0
Vargas	1984					0						0														0				0
Vargas	1988		1		1	2						0	1										1			2		1	1	2
Vargas	2003	1	1			2						0	1			1	1		1							4	1			0
Velasco	1995					0						0		1		1	1	1			1	1			1	7		1	1	2
Weitlaner	1952		1	1	1	3		1				1		1												1				0
Wentworth	1936					0						0														0				0
Wicke	1959				1	1						0		1		1	1		1				1			5		1	1	2
Wyatt	1998	1				1		1	1			2														0				0
		14	10	5	6	29	3	17	16	1	4	26	11	13	1	12	16	1	9	7	9	5	9	3	10	29	2	17	10	17

% of documents that mention item	90%	55	75%	46	50%	31	25%	15

* items grouped as they were mentioned in only one document

First author	Year	Reptiles							Sweets and sweeteners												Miscellaneous							
		Unespecific	Snakes	Turtles tortoise	Lizard	Alligator	Iguana or garrobo	Total mentions	Unespecific	Honey (bee, ant, wasp, maize, maguey, nopal)	Cakes	Pastries/sweet bread	Sugar and sugarcane (inc. Maize cane)	Cookies	Piloncillo or Panela/ raw brown cane sugar	Desserts	Dried or candied fruits and vegetables/ sugared fruit	Sweets/candy	Jam	Total mentions	Soups or broths (including instant soups)	Emulsifiers	Powdered chicken broth	Other snacks	Sage	Fast food (fried foods and processed snakcs)	Mexican snacks (tortas, sandwich, tacos)	Total mentions
Aguirre-Beltran	1994		1	1			1	3												0								0
Allen	1992							0		1			1			1				3								0
Almaguer Gonzalez	2018						1	1		1					1					2								0
Algert	1998							0												0								0
Anderson	1946							0												0								0
Avila-Nava	2017							0												0								0
Barros	1999							0		1										1								0
Beals	1943							0					1							1								0
Berdan	2017							0												0								0
Bertran-Vila	2010							0												0								0
Bertrán	2005							0		1	1		1	1					1	5	1		1			1		3
Bertrán	2006							0												0								0
Burgos-Monzon	2013							0												0								0
Carrera	2007							0			1			1		1		1	1	5	1					1	1	3
Casillas	1984							0		1										1								0
Castelló Yturbide	1986		1	1		1	1	3		1										1								0
Cook	1980	1			1		1	2		1			1							2								0
Crocker Sagastume	2004							0												0								0
Davalos Hurtado	1994		1	1			1	3		1										1								0
Flores	2010							0			1	1						1		3	1			1		1	1	4
Flores y Escalante	2004			1			1	2					1							1								0
García Chávez	2017							0			1	1		1		1		1		5	1	1		1		1	1	5
Garcia Uriguen	2012							0				1								1								0
Harris	2004							0												0								0
Katz	1990			1				1												0								0
Kittler	2007							0				1	1		1	1	1	1		6	1						1	2
Llamas	1935							0		1										1								0
Long-Solis	2005							0				1				1		1		3								0
Lopez Alonso	1974		1	1	1		1	4		1										1								0
Márquez-Morfín	1991			1			1	2		1										1								0
McMurry	1991							0					1							1								0
Méndez y Mercado	1993				1			1												0								0
Mercado	2012							0				1				1				2								0
Moreno-Altamirano	2017							0					1							1								0
Murtaugh	2008							0												0	1							1
Ojeda-Granados	2017							0												0								0
Ortiz de Montellano	1990		1				1	2		1										1								0
Quevedo	2004		1	1		1	1	3		1										1								0
Quiñones Tapia	2019						1	1							1					1	1							1
Quiroz	2004							0		1			1				1		1	4								0
Ravussin	1994							0					1							1								0
Rendon	1947							0					1		1					2	1							1
Robles-Ordaz	2017							0												0								0
Rodríguez Morán	2009							0												0								0
Roman et al	2013						1	1		1										1								0
Romero-Gwynn	1994			1				1				1	1							2					1			1
Santiago-Torres	2015							0					1							1	1							1
Santiago-Torres	2016							0												0								0
Santley et al.	1979	1	1					1												0								0
Shamosh	2014		1	1		1	1	3		1		1	1		1	1		1		6								0
Soustelle	1970			1			1	2		1										1					1			1
Tseng	1997							0												0								0
UNESCO	2010							0												0								0
Vargas	1984			1			1	2		1										1								0
Vargas	1988		1	1	1			3		1										1								0
Vargas	2003				1			1		1										1								0
Velasco	1995		1	1	1			3		1										1								0
Weitlaner	1952					1	1	1		1					1					2								0
Wentworth	1936							0												0								0
Wicke	1959		1				1	2		1										1					1			1
Wyatt	1998							0					1							1								0
		2	11	14	6	4	17	24	0	23	4	8	15	3	6	7	2	6	3	40	9	1	1	2	3	4	4	12

% of documents that mention item								
	90%	55	75%	46	50%	31	25%	15

		Herbs and condiments																														
First author	Year	Unespecific	Acedera/Xocoyoll/lengua de vaca	Achiote	Acuyo or hierba santa	Anise and aniseed or pericon	Arrayán	Chile/chilli,/peppers/salsa	Chipilin/chipil leaves	Cinnamon	Clove	Coriander	Cumin	Epazote	Garlic	Others*: avocado leaves, cochinilla, culantro, hibiscus, hierba de conejo	Lemon	Lime (fruit)	Lime (mineral)	Mustard	Nutmeg	Onion (including wild Xonacatl onion)	Oregano	Orejuela or veinacaztli	Parsley	Pepper	Salt/tequesquite/tequizquitl	Vanilla	Vinegar	Others*: mace, mint, moxtle, mecaxuchil, peppermint, rosita, soil breads, yoloxchitl, hediondilla	Zafron and azafrancillo	Total mentions
Aguirre-Beltran	1994							1																								1
Allen	1992	1						1							1					1					1							5
Almaguer Gonzalez	2018			1	1			1				1		1	1							1			1	1		1				10
Algert	1998							1																								1
Anderson	1946		1					1							1							1								1		5
Avila-Nava	2017							1																								1
Barros	1999							1														1					1					3
Beals	1943							1																								1
Berdan	2017							1																								1
Bertran-Vila	2010							1																								1
Bertrán	2005	1						1				1		1								1				1		1				7
Bertrán	2006							1							1		1					1	1				1					6
Burgos-Monzon	2013							1																								1
Carrera	2007	1						1														1										3
Casillas	1984							1											1													2
Castelló Yturbide	1986		1	1	1		1	1	1					1		1			1			1		1			1	1	1	1	1	16
Cook	1980							1									1															2
Crocker Sagastume	2004							1														1										2
Davalos Hurtado	1994			1				1						1								1				1		1	1			7
Flores	2010							1									1					1										3
Flores y Escalante	2004			1	1			1						1	1			1		1								1				8
García Chávez	2017							1																								1
Garcia Uriguen	2012							1						1									1									3
Harris	2004																															0
Katz	1990				1			1	1			1		1					1			1					1					8
Kittler	2007			1	1	1		1		1		1	1	1	1		1	1				1						1		1		14
Llamas	1935	1						1																1			1	1	1			6
Long-Solis	2005			1		1		1		1	1	1	1		1		1	1			1	1	1		1	1						15
Lopez Alonso	1974							1											1			1				1	1	1				6
Márquez-Morfín	1991							1																			1					2
McMurry	1991							1																								1
Méndez y Mercado	1993							1																								1
Mercado	2012							1																								1
Moreno-Altamirano	2017							1																								1
Murtaugh	2008																															0
Ojeda-Granados	2017							1																								1
Ortiz de Montellano	1990							1																								1
Quevedo	2004			1	1			1				1		1		1											1	1				8
Quiñones Tapia	2019							1														1										2
Quiroz	2004							1		1	1	1			1		1				1	1	1		1	1	1		1	1	1	15
Ravussin	1994							1														1										2
Rendon	1947							1		1				1	1		1		1			1					1					8
Robles-Ordaz	2017																															0
Rodríguez Morán	2009																															0
Roman et al	2013							1																								1
Romero-Gwynn	1994							1		1					1							1										4
Santiago-Torres	2015																															0
Santiago-Torres	2016	1						1							1							1										4
Santley et al.	1979							1											1													2
Shamosh	2014		1	1	1	1	1	1	1	1	1	1		1		1	1	1	1			1	1	1	1	1	1	1		1	1	24
Soustelle	1970							1																				1				2
Tseng	1997							1																								1
UNESCO	2010							1																				1				2
Vargas	1984							1																		1	1					3
Vargas	1988							1											1								1					3
Vargas	2003	1	1	1	1			1	1																	1	1		1			9
Velasco	1995			1	1			1						1													1	1				6
Weitlaner	1952				1			1						1		1	1	1				1					1					8
Wentworth	1936																															0
Wicke	1959							1														1						1				3
Wyatt	1998																															0
		6	4	10	10	3	2	54	4	6	3	8	2	13	11	4	9	5	8	2	2	23	5	3	5	9	16	14	5	5	3	54

% of documents that mention item	90%	55	75%	46	50%	31	25%	15

* items grouped as they were mentioned in only one document

Table S4. List of excluded articles (n=184)

Author	Title	Reason
Aceves-Martins et al.	Obesity-promoting factors in Mexican children and adolescents: challenges and opportunities.	Pattern(s) identified not referred as characteristic of Mexico
Afeiche M.C. et al.	Breakfast Dietary Patterns among Mexican Children Are Related to Total-Day Diet Quality.	No description of whole diet
Aguirre-Arenas et al.	Evaluación de los patrones alimentarios y la nutrición en cuatro comunidades rurales	Pattern(s) identified not referred as characteristic of Mexico
Akresh	Dietary assimilation and health among Hispanic immigrants to the United States.	Not excusive of Mexico/Mexican population
Anzo et al.	Impact of a digital Facebook campaign on the purchase and consumption of food in Mexican families with children under 12 years: A social marketing strategy.	Abstract/conference
Arenas-Monreal et al.	Cambios alimenticios en mujeres morelenses migrantes a Estados Unidos	No description of whole diet
Arias González	De lo que comían los tlatoque : una interpretaciónetnohistórica dentro del códice florentino y su comparación con la historia general de las cosas de Nueva España	Pattern(s) identified not referred as characteristic of Mexico
Arroyo et al.	Obesity and cultural environment in the Yucatan region...including commentary by Valdes R, Bourges H with author response	Abstract/conference
Arroyo et al.	Second Nestle Conference on Nutrition: Nutrition-Gene Interactions in Human Populations: the Amerindian Case, Mexico City, Mexico, January 29-30, 1998.	Abstract/conference
Arthur	The Florentine Codex: General History of the Things of New Spain	Food preparations and popular dishes
Avila-Nava et al.	Pre-Hispanic Mexican diet reduced dysbiosis and inflammation mediated by LPS in diet induced Obesity.	Abstract/conference
Avila-Nava et al.	A traditional Mexican diet decreased oxidative stress, improves carbohydrate and lipid metabolism and modifies the microbiota in a diet-induced obesity.	Abstract/conference
Aylwards	The Indigenous Foods of Mexico and Central America	Abstract/conference
Ayora Diaz et al.	The performance of Yucatecan subjectivity: cuisine, technology and taste	Food preparations and popular dishes
Ayora Diaz et al.	Translocality, globalisation and regionalism: how to understand regional Yucatecan gastronomy	No description of whole diet
Balcáza	Dieta, actividad física y estado de nutrición en escolares tarahumaras, México/ Diet, physicalactivity and nutritionstate in tarahumara schoolchildren in Mexico	No description of whole diet
Belderrama-Díaz et al.	Food practices of indigenous women according to the nutritional status and sex of their children	Not focused on food consumption

Batis et al..	Food acculturation drives dietary differences among Mexicans, Mexican Americans, and Non-Hispanic Whites	Pattern(s) identified not referred as characteristic of Mexico
Beltrán-Sánchez	Biological Risk in the Mexican Population at the Turn of the 21st Century	No description of whole diet
Benavides-Vaello et al..	Evaluating guiding questions for an ethnographic study of Mexican American women with diabetes	No description of whole diet
Benavides-Vaello et al.	Sociocultural construction of food ways in low-income Mexican-American women with diabetes: a qualitative study.	Food preparations and popular dishes
Bernard	Treasures at risk: Exploring regional cuisines in Mexico	Abstract/conference
Bertrán	La alimentación indígena de México como rasgo de identidad	Webpage
Blitz et al.	Dietary variability and social inequality at Monte Alban, Oaxaca, Mexico	Pattern(s) identified not referred as characteristic of Mexico
Bogin et al.	Globalization and children's diets: the case of the Maya of Mexico and Central America	Pattern(s) identified not referred as characteristic of Mexico
Bojorquez et al.	The social distribution of dietary patterns. Traditional, modern and healthy eating among women in a Latin American city.	Pattern(s) identified not referred as characteristic of Mexico
Brown et al.	Culturally tailored diabetes prevention in the workplace: Focus group interviews with Hispanic employees.	Not excusive of Mexico/Mexican population
Cachelin et al.	Associations between meal patterns, binge eating, and weight for Latinas.	Pattern(s) identified not referred as characteristic of Mexico
Callen	Dietary patterns in Mexico between 6500 a.C. And 1580 a.C.	No description of whole diet
Cano et al.	Elderly people in rural communities and traditional cuisine of Tabasco, Mexico.	Food preparations and popular dishes
Castellanos	Examining the Diet of Post-Migrant Hispanic Males Using the Precede-Proceed Model: Predisposing, Reinforcing, and Enabling Dietary Factors	No description of whole diet
Castro et al.	Ecodevelopmental contexts for preventing type 2 diabetes in Latino and other racial/ethnic minority populations.	Pattern(s) identified not referred as characteristic of Mexico
Cerqueira et al.	The food and nutrient intakes of the Tarahumara Indians of Mexico	No description of whole diet
Cinotto	The world on a plate: Globalization and national cuisines	Not excusive of Mexico/Mexican population
Coe et al.	The use of family rituals in eating behaviours in Hispanic mothers	Not focused on food consumption
Colby	The development of peer -led youth theater as a nutrition education tool to promote the healthy traditional Latino diet	No description of whole diet

Conor et al.	The plasma lipids, lipoproteins, and diet of the Tarahumara Indians of Mexico	No description of whole diet
Cox et al.	Eating the other: Ethnicity and the market for authentic Mexican food in Tucson, Arizona	Food preparations and popular dishes
Cravioto et al.	Composition of typical Mexican foods	No description of whole diet
Crocker Sagastume et al.	El costumbre y la soberanía alimentaria Wixárika	Duplicate report
Crockett et al.	Risk factors and consequences related to adolescent overweight among African-American, Caucasian-American and Mexican-American adolescents ages twelve through nineteen	Pattern(s) identified not referred as characteristic of Mexico
Daltabuit	Cambios de la dieta familiar en Yalcoba, Yucatán	No description of whole diet
de Leon	Amino acid composition of some Mexican foods	No description of whole diet
Dellasega et al.	Identifying culture-specific barriers to care through the Hispanic/Latino diabetes needs questionnaire (HLDNQ).	Abstract/conference
Dewalt et al.	Food and household ecology in a Mexican Community	Pattern(s) identified not referred as characteristic of Mexico
Dewey	Dietary change among migrant and nonmigrantMexican-American Families in Northern Califormia	No description of whole diet
Diaz et al.	Cultural conflicts in the weight loss experience of overweight Latinos.	Pattern(s) identified not referred as characteristic of Mexico
Dipnall et al.	The association between dietary patterns, diabetes and depression	Not excusive of Mexico/Mexican population
Dirks et al.	Diet and nutrition in poor and minority communities in the United States 100 years ago	Not excusive of Mexico/Mexican population
Dondero et al.	Generational status, neighborhood context, and mother-child resemblance in dietary quality in Mexican-origin families.	Pattern(s) identified not referred as characteristic of Mexico
Dondero et al.	Dietary Assimilation among Mexican Children in Immigrant Households: Code-switching and Healthy Eating across Social Institutions	No description of whole diet
Eilat-Adar et al.	Dietary patterns and their association with cardiovascular risk factors in a population undergoing lifestyle changes: The Strong Heart Study.	Not excusive of Mexico/Mexican population
Escalante Gonzalbo	La vida urbana en el periodo clasico mesoamericano	Not focused on food consumption
Espejel Blanco et al.	Alimentos tradicionales en Sonora, México: factores que influyen en su consumo	Food preparations and popular dishes
Estrada et al.	Comparison of analized and calculated energy, fat, protein, dietary fiber, iron and zinc values in diets from different socioeconomic levels in northern Mexico.	No description of whole diet

Evans	Traditional Foods and Practices of Spanish-Speaking Latina Mothers Influence the Home Food Environment: Implications for Future Interventions	Not excusive of Mexico/Mexican population
Falcon et al.	Dietary intake, maternal anthropometric parameters and birth weight, in a mother-child sample.	Abstract/conference
Falkowski et al.	More than just corn and calories: a comprehensive assessmentof the yield and nutritional content of a traditional Lacandon Maya *milpa*	No description of whole diet
Farga et al.	Historia de la comida en México	Food preparations and popular dishes
Fazzino et al.	Continuity and change in Tohono O'odham food systems: implications for dietary interventions	Not excusive of Mexico/Mexican population
Flores et al.	Making tortillas without lard: Latino Parents' perspectives on healthy eating, physical activity and weight management strategies for overweight Latino children.	Not focused on food consumption
Galvan et al.	Western habits in Mexico spur increase in diabetes.	Pattern(s) identified not referred as characteristic of Mexico
Gann	Ancient Cities and Modern Tribes: Exploration in Maya Lands.	Not excusive of Mexico/Mexican population
Garcia Acosta	[Mesoamerican food and processing techniques]. [Spanish]	Not excusive of Mexico/Mexican population
Garcia et al.	High fiber diet prepared with regional foods as an aid in the control of patients with diabetes [Spanish]	Pattern(s) identified not referred as characteristic of Mexico
García-Rivas	Cocina prehispánicamexicana	Food preparations and popular dishes
Gilboy et al.	Cultural immersion experience: Promoting an understanding of Mexican American nutrition and food traditions.	Food preparations and popular dishes
Gonzalez et al.	The Mexico City Diabetes Study: a population-based approach to the study of genetic and environmental interactions in the pathogenesis of obesity and diabetes	No description of whole diet
Gonzalez-Castell et al.	Contribution of processed foods to the energy, macronutrient and fiber intakes of Mexican children aged 1 to 4 years. [Spanish]	No description of whole diet
González de la Vara	La cocina mexicana a través de los siglos. Tomo II	Pattern(s) identified not referred as characteristic of Mexico
Goto et al.	Understanding Possible Roles of Locally-grown Ethnic Produce in Dietary Practices and Food Cultures: An Exploratory Study.	Not excusive of Mexico/Mexican population
Gotz et al.	Feedings of the prehispanic Maya from the view of zooarchaeology	No description of whole diet
Greenberg et al.	You are what you eat: Ethnicity and change in Yucatec immigrant house lots, Quintana Roo, Mexico	No description of whole diet

Grijalva Haro et al.	Chemical composition, dietary fiber and mineral content of frequently consumed foods in Northwest Mexico	Pattern(s) identified not referred as characteristic of Mexico
Guarnaccia et al.	We eat meat every day': Ecology and economy of dietary change among Oaxacan migrants from Mexico to New Jersey.	Pattern(s) identified not referred as characteristic of Mexico
Hanni et al.	Steps to a Healthier Salinas: Targeting the taqueria: Implementing healthy food options at Mexican American restaurants.	No description of whole diet
Harley et al.	Time in the United States, social support and health behaviors during pregnancy among women of Mexican descent.	Pattern(s) identified not referred as characteristic of Mexico
Herrera-Flores et al.	La alimentacion de los antiguos mayas de la peninsula de Yucatan: consideraciones sobre la identidad y la cuisine en la epocaprehispanica	No description of whole diet
Heuman et al.	Rural Hispanic populations at risk in developing diabetes: Sociocultural and familial challenges in promoting a healthy diet.	Pattern(s) identified not referred as characteristic of Mexico
Hindley	Reviving the Food of the Aztecs	No description of whole diet
Horowitz	How do urban African Americans and Latinos view the influence of diet on hypertension?	Pattern(s) identified not referred as characteristic of Mexico
Izquierdo et al.	Food perceptions among indigenous persons adult two Mayan communities. [Spanish]	Not focused on food consumption
Ituriaga	Las cocinas de Mexico	Pattern(s) identified not referred as characteristic of Mexico
Jardines et al.	Typical dishes consumed in Sonora: regionalization and nutrient contribution. [Spanish]	Food preparations and popular dishes
Jiménez Aguilar et al.	Dietary intake and adequacy in Mexican preschool children: National Health and Nutrition Survey 2012	Pattern(s) identified not referred to as characteristic of Mexico
Jones et al.	Dietary fiber, Hispanics, and breast cancer risk?	No description of whole diet
Juárez López	La lenta emergencia de la comida mexicana	Not focused on food consumption
Kaiser et al.	Acculturation of Mexican-American mothers influences child feeding strategies.	No description of whole diet
Kanter	Gender disparities in dietary intake and obesity prevalence among Mexican adults: A cross-country comparison between Mexico and the U.S.	Pattern(s) identified not referred as characteristic of Mexico
Kilanowski	Migrant farmworker mothers talk about the meaning of food.	Food preparations and popular dishes
King	Corn, Beer, and Marine Resources at Casas Grandes, Mexico: An Analysis of Prehistoric Diets Using Microfossils Recovered from Dental Calculus	No description of whole diet
Knepp	Tamaladas and the role of food in Mexican-immigrant and Mexican-American cultures in Texas.	No description of whole diet

Koehler et al.	Core, secondary, and peripheral foods in the diets of Hispanic, Navajo, and Jemez Indian children.	Not exclusive of Mexico/Mexican population
Kuczmarski	Food usage among Mexican American, Cuban, and Puerto Rican adults: Findings from the Hispanic NHAHES-Health and Nutrition Examination Survey	Pattern(s) identified not referred as characteristic of Mexico
Lagana et al.	Preventing low birthweight: cultural influences on Mexican immigrant and Mexican-American prenatal care. A community study.	Pattern(s) identified not referred as characteristic of Mexico
Laudan	Glancing backward to Spain or Looking Forward to Mexico	No description of whole diet
Lomntiz et al.	Planeación y tradición: La cultura de la alimentación en México	No description of whole diet
Long	Conquista y comida. Consecuencias del encuentro de dos mundos.	Pattern(s) identified not referred as characteristic of Mexico
Lopez-Ojeda	Characterization of the main trends of Mexican gastronomy in the framework of new social scenarios	Food preparations and popular dishes
López-Pentecost	Association between diet quality and obesity-related cancer in postmenopausal Hispanic women_ Results from the women's Health Initiative (WHI)	Duplicate report (cited a single reference)
Martínez et al.	La alimentación en México: un estudio a partir de la Encuesta Nacional de Ingresos y Gastos de los Hogares	Pattern(s) identified not referred as characteristic of Mexico
Martinez et al.	Comiendo bien: The production of Latinidad through the performance of healthy eating among Latino immigrant families in San Francisco.	Pattern(s) identified not referred as characteristic of Mexico
McArthur et al.	Maintenance and change in the diet of Hispanic immigrants in eastern North Carolina.	Not exclusive of Mexico/Mexican population
McClain et al.	Life course influences on food provisioning among low-income, Mexican-born mothers with young children at risk of food insecurity	No description of whole diet
McCrossin	The fat of the (border)land: Food, flesh, and Hispanic masculinity in Willa Cather's Death comes for the archbishop.	No description of whole diet
Melendez-Torres et al.	Traditional regional cuisine as an element of local identity and development: a case study from San Pedro El Saucito, Sonora, Mexico	Food preparations and popular dishes
Melius et al.	Exploring U.S. Hispanic parents' length of time in the United States: Influences on obesity outcomes among U.S. Hispanic children.	Pattern(s) identified not referred as characteristic of Mexico
Melo Ruiz et al.	Mexican native food contribution to the word.	Abstract/conference
Monarrez-Espino	Perception of food and body shape as dimensions of western acculturation potentially linked to overweight in Tarahumara women of Mexico	Not focused on food consumption
Monge	Western and Modern Mexican dietary patterns are directly associated with incident hypertension in Mexican women: a prospective follow-up study	No description of whole diet

Monge et al.	Dietary patterns and incident hypertension in Mexican women.	Abstract/conference
Monroe	The effects of dietary and other lifestyle behaviors on the risk of colorectal cancer among the Mexican -origin Latino population	No description of whole diet
Moreno-Altamirano et al.	Diabetes tipo 2 y patrones de alimentación de 1961 a 2009: Algunos de sus determinantes sociales en México	No description of whole diet
Moreno-Altamirano et al.	La transición alimentaria y la doble carga de malnutrición: Cambios en los patrones alimentarios de 1961 a 2009 en el contexto socioeconómico mexicano	No description of whole diet
Muñoz-Ibarra et al.	Food profile of a rural population in Michoacan and its relation with obesity, diabetes and hypertension. [Spanish]	Pattern(s) identified not referred as characteristic of Mexico
Nado	Dietary Practices, Socioeconomic Status, and Social Mobility at Teotihuacan, Mexico	No description of whole diet
Nalda et al.	Paleodieta en Dzibanché y Kohunlish: diferencias y tendencias preliminares	No description of whole diet
Narchi et al.	Social constructs, identity, and the ecological consequences of carne asada	No description of whole diet
Ojeda-Granados et al.	A regionalized genome-based mexican diet normalizes hypertriglyceridemia and improves insulin resistance in subjects with metabolic risk factors for chronic disease.	Abstract/conference
Ortega-Munoz	Dental Health and Alimentation Among the Quintana Roo Mayas: Coastal and Inland Sites of the Classic-Postclassic Periods	Not focused on food consumption
Ortega	Dietary risk factors and acculturation among Mexican migrant (Southwest United States) and non-migrant (Northwest Mexico) women	Pattern(s) identified not referred as characteristic of Mexico
Ortiz-Hernandez et al.	Cambios en factores relacionados con la transición alimentaria y nutricional en México	Pattern(s) identified not referred as characteristic of Mexico
Palerm et al.	Agricultura y Civilización en Mesoamérica	Not focused on food consumption
Pareo-Tubbeh et al.	Comparison of energy and nutrient sources of elderly Hispanics and non- Hispanic whites in New Mexico.	Not exclusive of Mexico/Mexican population
Pérez I et al.	Percepción de la imagen corporal y prácticas alimentarias entre indígenas Mayas de Yucatán, México/ Bodyimageperception and foodpractices of Mayan indigenous of Yucatan, Mexico	Pattern(s) identified not referred as characteristic of Mexico
Pérez Izquierdo et al.	Frecuencia del consumo de alimentos industrializados modernos en la dieta habitual de comunidades mayas de Yucatán, México	No description of whole diet
Perez et al.	Factors contributing to the adult population dietary change in indigenous maya.	Abstract/conference
Perez-Rodriguez et al.	Dietary patterns associated with incidence of metabolic syndrome and its components.	Abstract/conference

Perez et al.	Tasting culture: Food, family and lavour in Greater Mexico	Food preparations and popular dishes
Popovic-Lipovac et al.	A review on changes in food habits among immigrant women and implications for health.	Not exclusive of Mexico/Mexican population
Porcasi et al.	Pre-hispanic-to-colonial dietary transitions at Ezatlan, Jalisco, Mexico	No description of whole diet
Posadas et al.	Diet and caloric sufficiency in the migrant indigenous population of the Altos Region of Chiapas, Mexico. [Spanish]	Pattern(s) identified not referred as characteristic of Mexico
Price et al.	Calakmul as a central place: Isotopic insights on urban Maya mobility and diet during the first millenium	No description of whole diet
Ramirez et al.	Questioning the Dietary Acculturation Paradox: A Mixed-Methods Study of the Relationship between Food and Ethnic Identity in a Group of Mexican-American Women	Food preparations and popular dishes
Reid	Nutrient intake of Pima Indian women: relationships to diabetes mellitus and gallbladder disease	Not exclusive of Mexico/Mexican population
Reininger et al.	Healthy eating patterns associated with acculturation, sex and BMI among Mexican Americans.	Pattern(s) identified not referred as characteristic of Mexico
Rice et al.	The effects of acculturation, diet, and workload on bone density in premenopausal Mexican American women	No description of whole diet
Rivera	Mexico attempts to tackle obesity: the process, results, push backs and future challenges	Food preparations and popular dishes
Rivera et al.	Nutrition transition in Mexico and in other Latin American countries	No description of whole diet
Robles et al.	Predictors of receiving and adhering to hypertension therapeutic and lifestyle modification management.	Abstract/conference
Rodríguez Rivera	La comida en el México antiguo y moderno	Food preparations and popular dishes
Rodriguez-Moran et al.	Cardiovascular Risk Factors and Acculturation in Yaquis and Tepehuanos Indians from Mexico.	Not focused on food consumption
Romano et al.	Evaluation of dietary patterns and nutritional status of families living in an indigenous population in Chiapas, Mexico.	Abstract/conference
Romero-Contreras et al.	Formación del patrimonio gastronómico del Valle de Toluca, México	Food preparations and popular dishes
Romero-Gwynn et al.	Dietary acculturation among Latinos of Mexican descent	Duplicate report
Sahagún	Historia general de las cosas de Nueva España	Food preparations and popular dishes
Sanchez	Campesino; Food Consumption in the Context of Migration and Remittances	Pattern(s) identified not referred as characteristic of Mexico

Sanders	The agricultural history of the Basin of Mexico. In The Valley of Mexico: Studies in PreHispanic Ecology and Socie	Not focused on food consumption
Sanders	The Basin of Mexico: Ecological Processes in the Evolution of a Civilization	Not focused on food consumption
Santiago-Torres et al.	Does genetic ancestry influence the metabolic response to a traditional Mexican versus U.S. diet? A randomized crossover feeding trial among first and second generation women of Mexican descent	Abstract/conference
Santiago-Torres et al.	Genetic ancestry in relation to the metabolic response to a US versus traditional Mexican diet: a randomized crossover feeding trial among women of Mexican descent	Duplicate report
Schulz et al.	Effects of traditional and Western environments on prevalence of type 2 diabetes in Pima Indians in Mexico and the U.S. Diabetes Care	Pattern(s) identified not referred as characteristic of Mexico
Serrano-Cruz et al.	Factors associated with the consumption of traditional foods in central Mexico	No description of whole diet
Shattock	The Peninsula Of Yucatan: Medical, Biological, Meteorological And Sociological Studies	Pattern(s) identified not referred as characteristic of Mexico
Siguiura	La cocina mexicana a través de los siglos. Tomo I	Pattern(s) identified not referred as characteristic of Mexico
Smith et al.	Selected traditional and contemporary foods currently used by the Pima Indians	Not exclusive of Mexico/Mexican population
Smith-Morris	The traditional food of migrants: Meat, water, and other challenges for dietary advice. An ethnography in Guanajuato, Mexico	No description of whole diet
Sofianou et al.	Differences in diet pattern adherence by nativity and duration of US residence in the Mexican-American population	Pattern(s) identified not referred as characteristic of Mexico
Solomons et al.	Armonización de las Recomendaciones Nutricionales para Mesoamérica: ¿Unificación regional o individualización nacional?	No description of whole diet
Somerville et al.	Applying new approaches to modelling diet and status: isotopic evidence for commoner resiliency and elite variability in the classic Maya lowlands	Not focused on food consumption
Soto et al.	Exploring how bicultural and assimilated children of Mexican origininfluence their Latina mothers' diet: Perspectives from mothers and children	Not focused on food consumption
Soto et al.	Family environment, children's acculturation and mothers' dietary intakeandbehaviors among Latinas: An autoregressive cross-lagged study	Not focused on food consumption
Stoddard et al.	The influence of indigenous status and community indigenous composition on obesity and diabetes among Mexican adults.	Pattern(s) identified not referred as characteristic of Mexico
Sugiyama	Feeding Teotihuacan: integrating approaches to studying food and foodways of the ancient metropolis	No description of whole diet
Super et al.	The history and culture of food and drink in the Americas: Mexico and highland Central America	Not exclusive of Mexico/Mexican population

Sussner et al.	The influence of immigrant status and acculturation on the development of overweight in Latino families: A qualitative study. [References].	Not exclusive of Mexico/Mexican population
Tiedje et al.	A focus group study of healthy eating knowledge, practices, and barriers among adult and adolescent immigrants and refugees in the United States. [References].	Pattern(s) identified not referred as characteristic of Mexico
Togo Luna et al.	Comparación del consumo de alimentos de niños que habitan una zona urbana y una rural en la población de Arandas, México	No description of whole diet
Torres-Aguilar et al.	Factors correlated to protective and risk dietary patterns in immigrant Latino mothers in non-metropolitan rural communities. [References].	Pattern(s) identified not referred as characteristic of Mexico
Tozzer	A Comparative Study of the Mayas and the Lacandones.	Not focused on food consumption
Tseng	Food intake patterns and gallbladder disease in Mexican Americans.	Not exclusive of Mexico/Mexican population
Valencia et al.	The Pima Indians in Sonora, Mexico	Abstract/conference
Valle Berrocal	La alimentación en una comunidad Xochimilca: Santa Cecilia Tapetlap	Pattern(s) identified not referred as characteristic of Mexico
Vargas	El encuentro de dos cocinas: México en el siglo XVI	Pattern(s) identified not referred as characteristic of Mexico
Vargas	Old and new transitions and nutrition in Mexico	Not focused on food consumption
Vargas et al.	La alimentacion en México durante los primeros años de la Colonia	No description of whole diet
Velasco et al.	In quicua, se come: los alimentos en la cosmovisión mexica	No description of whole diet
Viladrich et al.	Picking fruit from our backyard's trees: The meaning of nostalgia in shaping Latinas' eating practices in the United States. [References].	Not exclusive of Mexico/Mexican population
Warinner	Disease, demography, and diet in early colonial new Spain: investigation of a sixteenth-century Mixtec cemetery at teposcolulayucundaa	No description of whole diet
Wing	A comparison of Olmec and Maya foodways	No description of whole diet
Wolff et al.	Maternal eating patterns and birth weight of Mexican American infants.	Pattern(s) identified not referred as characteristic of Mexico
Wyatt	r- and γ-Tocopherol Content of Selected Foods in the Mexican Diet: Effect of Cooking Losses	No description of whole diet
Zizumbo-Villareal	The Archaic Diet in Mesoamerica: Incentive for Milpa Development and Species Domestication	Not focused on food consumption
Zulauf et al.	Indigenous cuisine: An archaeological and linguistic study of colonial Zapotec foodways on the Isthmus of Tehuantepec	Food preparations and popular dishes

Table S5. Definition of the traditional Mexican diet according to different authors

First author (ref.)	Year	Diet definition
**Literature reviews**		
Aguirre-Beltrán [38]	1994	Basic foods are maize (consumed as *tortillas*), beans, *chile*, squash and wild vegetables. Complemented with insects, batrachian, rodents, reptiles. *Axayácatl*[1] and its eggs(*ahuauhtli*[1]), grasshoppers, *chicatana* ants and their larvae, *maguey* worms, *jonote*[1], *guásimo*[1], other worms, *jumiles*[1], iguanas, turtles, snakes, tadpoles, rats, *tlacuaches*[2], skunks, dogs, cacao, wheat, beef, cabbage, herbs (like *malva*), fruits (like *uste*), *pulque*[3].
Allen [39]	1992	Maize (consumed as *tortillas*, soups, drinks, and *tamales*), vegetables (beans, squash, potatoes, *nopales*, tomatoes, peppers and herbs), fruits (pineapples, avocado, plums, peanuts and *jícamas*), animal foods (deer, wild pigs, rabbits, and lake birds), and beverages (made from maize, seeds, and cocoa), honey, juice of maguey or maize, *pulque*[3](limited intake), sugar, milk, fat, garlic, parsley, celery, radishes, mustards greens, rice, legumes.
Algert [40]	1998	Basic foods are maize, beans and squash. Complemented with cactus, agave, wild and leafy plants, tubers, *chili*, amaranth, avocado, and guava.
Almaguez-González [41]	2018	Basic foods are maize (consumed as *atole*, *pozole*, *tamales,* and *tortillas* and other *tortilla* varieties), beans, squashes and *chile*. Complemented with water;vegetables (*nopales*, *quelites*, *quintoniles*, purslane, green beans, *romeritos*, *huauzontle*, red tomato, *ciltlali*tomato, green tomato, *miltomate*, bell peppers,*chayote*, *chilacayote*, *colorín* blossoms, *flor de izote*, *jícama*, watercress, *chaya*, *huitlacoche*, *achiote*, *epazote*, vanilla, *acuyo*, mushrooms, pepper, onion, garlic, coriander and parsley); pulses (broad beans) and nuts (pumpkin seeds, chia seeds, peanuts and pine nuts); fruits (*guanábana*, prickly pear, papaya, black *zapote*, *chicozapote*, *mamey*, guava, *tejocote*, *capulín*, pineapple, *anona*, *xoconoxtle*, *chirimolla*, *nance*, yellow plum, *pitahaya*, and blueberries and blackberries); avocado; amaranth; tubers (sweet potato, yucca and *chinchayote*); fish (catfish, trout, white fish, shark, sea bass, devil ray, *mojarra*, saw fish), seafood (crab, mussels, oysters, *acamaya*, octopus, and shrimp), and chicken eggs; beverages (maguey juice or *aguamiel*, *pozol*, chocolate, and *tesgüino*), cheeses (*requesón*, *Oaxaca*, white, *Panela*, *asadero*, *Chiapas*, *ranchero*, goat), and sweeteners (bee and maguey honey, *piloncillo*), poultry (turkey and chicken), insects (grasshoppers, maguey worms, *chinicuiles*, *chicatana*and honey ants, *jumiles*), and red meat (venison, rabbit, iguana, pork, beef, lamb, chevon).
Avila Nava [42]	2017	Basic foods are corn, black beans, and seeds (pumpkin seeds, chia seeds). *Nopal*[4], chili, turkey, fish, fowl and insects, tomato.
Barros [43]	1999	Maize, beans, squash, *chile*, tomato, *chayote*[4], *jicama* [5], *xonácatl* onion, chia, amaranth, fish eggs, water-fly, *Spirulina* algae, turkey, dogs, salt, quail, rabbit, hare, venison, poultry, maguey honey, bee honey, potato, sweet potato, *huauzontle*[6], *quelites*[6], cacao, *atole*[7,8], *zapotes*[5], fish, seafood, plums, *capulines*[5]and *guavas*[5].

Berdan [44]	2017	Basic foods are maize, beans, squashes, *chiles*, and other vegetables. Supplemented with turkeys, dogs, rabbits, deer, birds, fish and other aquatic sources, insects and their larvae, and beverages (*atolli*[8], cacao, and *pulque*[3]).
Bertran-Vilà [45]	2010	Maize (consumed as *tortillas*), beans, spicy *salsa*, vegetables, occasional animal foods (eggs, milk, cheese, small portions of beef) and fruit.
BertranVilà [46]	2005	Basic foods are maize(consumed as *tortillas*, *atole* or *pozol*), beans, *chile*, vegetables, limited meats and eggs. Complemented with green and red tomato, squash, purslanes, *quintoniles*[6], *chayote*[4], carrot, radish, mushrooms,*nopal*[4], chicken, beef, cheese, bread, pasta, biscuits, rice, coffee, cacao, honey bee, vanilla, sugar, limited intake of fruits (citrus fruits, banana, peach, apple, mangoes), canned tuna, cakes, soda, sugary drinks, fried foods, potato, sweet potato, *quelites*[6], *chaya*[6], avocado, onion, coriander, yucca, pepper, *epazote*[9], fish, shrimp, snails, *chicatana*ants, grasshoppers, *maguey* worms, aromatic herbs, canned sardines, instant soups, powdered chicken broth, broad beans, candies, legumes (lentils, *ibes*),heart of palm, yucca, water, fruit drinks, chocolate drinks, limited alcoholic drinks (*pulque*, *mezcal*, *tesgüino*, *taberna*).
Bertran [47]	2006	Basic foods are maize (consumed as *tortillas*), beans, and squash. Complemented with *chile*, salt, wild plants (squash leaves and *quelites*), red tomato, green tomato, garlic, onion, oregano, lemon, small amount of animal-source foods (cheese, eggs, meat, fish, chicken and insects), lard, avocado, *atole*[7,8],fresh fruit or fruit juice.
Casillas [48]	1984	Basic foods are maize (consumed on the cob, as *pozole*, *tortillas* and *tortilla* varieties, *tamales and atole*), squash, *chile*, and amaranth. Beans, avocado, plums, cacti, *mezcal* [3], *mezquite*leaves and *mezquite* seeds, *nopal* [4],*maguey* leaves, prickly pear, maize juice, turkey, tomato (red tomato, green, *costomate*, and *miltomate*), guava, peanuts, *Setaria*grass, lime, squash seeds, *huauzontle*[6], *quelites*[6] and *quintoniles*[6], fruits (*zapote*, *nanche*, *cosahuico*, *capulines*, pineapple, *chirimoya*, *mamey*), cacao, bee honey, turkeys, dogs, *tlacuache*[2], armadillo, rabbit, squirrels, gophers, rats, mice, *cacomixtle*[2], weasel, skunk, fish and seafood, tadpoles, frogs, *axolotl* [10], *acocilli*[11], *axacayácatl*insect, water-fly (*amoyotl*), *michpillimichpilteten*(fish eggs), *izcauitli*worms, waterpoultry (ducks, *ánsares, zacacintli, atzizicoílotl, atotolin, acitlin, tenitzli, axoque, couxin, quetzaltecoloton, metzacanauhtli, guacoxtli, ehecatótotl, amanacoche, yacatextli, tzitzuia, xalquani, nacaztzona, zolcanauhtli, chilcanauhtli, chalalacli, yacapatláhuac, pipitztli),* insects, *spirulina* algae.
CastellóYturbide [49]	1986	Basic foods are maize (consumed as *atole*, *tamal*, *tortillas*, popcorn, *pinole*, *pozol*), squash, beans, and *chile*. Complemented with amaranth, plants (squash stems,*chayote*,*nopal*, *cuitlacochin*,*chinchayote*, *huauzoncle*, *quelites*,*quintoniles*, purslanes, red and green tomato, sweet potato, potato, *ayatito*, avocado, *guaje*), herbs, condiments and colorants (honey, salt, *tequezquite*, *achiote*, maguey vinegar, *cochinilla*, *añil*, *azafrancillo*, vanilla, *orejuela*, *acedera*, *acuyo*, *arrayán*, *xonácatl* onion, *chipilín* leaves, *epazote*, *hierba de conejo*, *lengua de vaca*, *papaloquiliti*), blossoms (squash, *colorín*, *botón de biznaga*, *flor de mayo*, *izote*), fruits (*cuajinicuil*, *xícama*, *chirimoya*, *chicozapote*, *zapote*, guava, *tejocote*, *capulín*, plum, *guanábana*, *nanche*, *mamey*, papaya, coconut), mushrooms, algae (*Spirulina*, *amoxtle*, *cuculito del agua*), snacks (peanuts, *mezquite* pods), beverages (from maize, chia, *bledos* and cacao seeds), fermented beverages (from maize, chia, and maguey; *charagua*, *charape*, *sototl*, *teshuino*, *chicha*, *piznate*, *chorote*, *popo*, *taxcalate*, *tejate*, *mejengue*, *guásimo*, *tepache*, *chilacayota*, *huikimo*, *tuba*, blossomliquors, *colonche*, *bote*, *verde de xico*, *pulque*, minerals (lime, salt, *tequezquite*, soil breads), insects (*atetepitz*, maize andmaguey worms, *chimicuile*worms,*axayácatl*, *ahuautli*, water-fly larvae and pupa, grasshoppers, *jumiles*, *ticocos*, *cupiches*, honey ant, *escamoles*, *chicatana*

		ants, *cuetla*, wasps), crustaceans (shrimp, *acocil*), fish (*cazón*, catfish, *mextlapique*), batrachian (*ajolote*, frog), reptiles (iguana, turtle, snake, alligator), poultry (duck, parrot, *chichicuilote*, turkey), mammals (dog, squirrel, *tepeitzcuintli*, gopher, rabbit, monkey, *tlacuache*[2], wild boar, venison, armadillo).
Cook [50]	1980	Basic foods are maize (consumed as *tortillas*, *tamales* or *atole*), beans, squash, *chile*, *chía*[12], *cactus*[4], prickly pear, *maguey*[4](as *pulque*[3], blades and heart), and roots (mostly sweet potatoes). Fruits, vegetables, bee honey, ducks, dogs, turkeys, mammals, birds, fish, reptiles, amphibians, crustaceans, insects, worms, nuts, berries, greens, iguanas, lizards, grubs, lettuce, radishes, carrots, cabbage, apples, quinces, oranges, lemons, peaches, apricots, walnuts, bananas, guava, peanuts, sugarcane, chicken, tomatoes, chicken eggs, sheep, goat.
Dávalos Hurtado [51]	1994	Basic food is maize (consumed on the cob, in soups, *tortillas, tamales*, *pinole*, *pozol*and *atole*). Complemented with vegetables (beans, squash, *chilacayote*, *chayote*,potato, sweet potato, *guamamote*, *cuajilote*, *cuapinole*, *huachacote*, *mesquite*, *nopal*), herbs (*quelites*, *quintoniles*, *malva, huauzontle*), mushrooms, *cacomite*[13], maguey core, seasonings (*chile*, green tomato, red tomato, *miltomate*, *jaltomate*, squashseeds, *xonácatl* onion, *achiote*, *xoconoztli*, Tabasco pepper). Animals: mammals (venison, *coyametl*boar, rabbit, hare, badger, weasel, marten, squirrel, otter, *tlacuache*[2], armadillo, raccoon, bear, tapir, *tepezcuintle*, dogs), poultry (turkey, pheasant, pigeon, quail, *chachalacas*, partridges, turtledove, coots,*ánsares*, *ánades*, ducks, mergansers), frogs, snakes, turtles, iguanas and their eggs, fish (*pámpanos*, *pargos*, *guachinangos*, *congrios*, *sollos*, *besugos*). Fruits (pinepple, *mamey*, *chirimoya*, *guanábana*, *anona*, avocado, *zapote*, guava, *tejocote*, *capulín*, *xocotl* plum, *nance*, *hobo*, *pitahaya*, prickly pear, papaya, *jícama*, peanuts), *tecuitlatl*algae, water-fly eggs, worms (*maguey* worms, *ocuiliztac, atetepitz, atopinan, ahuihuitl),epazote*[9]*,maguey* juice, chia[12], beverages (from maize, chia[12], *bledos*[14], and cacao), vanilla, maize honey, maguey honey, bee honey, alcoholic beverages (from maize and chia[12];limited intake of *pulque), maguey* vinegar.
Flores Y Escalante [52]	2004	Basic foods are maize (consumed as *tortillas*, *atole*, *totopoxtle*, *pinole*, on the cob, *tlacoyos, tamales, zacahuil, pozole, chicha*, *esquite*, *huitlacoche*, *tesgüino, chicha*), *chile*, beans, squash, tomatoes (red, *jaltomate, jaltenate, costomate, miltomate*, *Milpa* tomato, green), vanilla, chocolate drinks, *nopal* [4], prickly pear, *pulque* [3], turkey, avocado, and potato. Vegetables: cabbage, lettuce, spinach, cucumber, aubergine, musk mallow, asparagus, watercress, garlic, artichoke. Roots: beetroot, parsnip, carrot, radishes, *mandioca,* sweet potato, *oca*, *olluco*, *añu*, *boniato*yucca, *ñame.* Fruits: apple, pear, plum, cherries, grapes, lime, fig, *chirimoya*, papaya, pineapple, *guanábana*, pickle, strawberries, raspberry, *capulín*, *tejocote*, guava,*mamey*, *zapote.* Nuts and seeds: walnuts, flax seed, olives, sesame seeds,cashew nuts, Brazil nuts, peanuts, squash seeds. Legumes: peas, lentils, soybeans, broad beans, *canigwa*, *tarwi*, *molle*. Cereals: wheat, barley, rye, oats, millet, rice, quinoa. Condiments: mustard, sugarcane, bellpeppers, *epazote*, *pápalo*,*acuyo*, *pipicha*, *guaje*. Beverages: *mate*, *guayusa*, *chicha*, *tuba.* Others: *chilacayote* [4], *chayote* [4], *chinchayote*[15], chiaseeds, tamarind, mushrooms, squash blossoms, *papaloquelite*[6],amaranth, *achiote*[9], *jícama*[5], *quelites*[6],*quiote*[6],grasshopers, *acociles*[11], *ajolote*[10], *atepocate*[10], *chichicuilote*[16], ducks, partridges, iguanas, venison, fish, pigeons, pheasant, dogs (*itzcuintli,*), *chichicaxtle*turtles, rabbits,*biznagas*[4] and othercacti, yucca and *colorín*blossoms, *cacomixtle*[2], *tlacuache*[2], armadillo,*coyol*[5], purslane.

García Urigüen [53]	2012	Maize (consumed as *tamales, atole,* and *tortillas)*, beans, *chile*,squash, amaranth, *nopal*[5], purslane, avocado, tomato, *guaje*[6], fruits (*tejocote*, *capulín*, plum, and white *zapote*), aromatic herbs (*epazote* and oregano), turkey, hare, venison, dog, rodents, poultry, fish, legumes, pork, chicken, beef, chocolate, 'sweet bread'.
Harris [54]	2004	Corn tortillas and corn products, beans, rice, bread, fruits, vegetables, eggs, fish, shellfish, beef, pork, poultry, goat, lard, potatoes.
Katz [55]	1990	Basic foods are beans, maize (consumed as *tortillas, tamales,* and *atole*), squash, *chile*, cacao, wild plants, and fruits. Complemented with mushrooms, *nopales*[4], prickly pear, wild onion, *Setaria*grass, *nanches*[5], *susí*seeds, *huizache*[4], *mezquite*[4], acorns, turtles, quail, rabbits, venison, *maguey*[4], peccary, *tlacuahces*[2], raccoons, squirrels, iguanas, turtledoves, pigeons, grasshoppers, *chicatana*ants, maize worms, armadillo, coati, turkey, hares, frogs, lime, salt, *papaloquelites*[6], *tejocote*[5], guava, avocado, *quamochotl*[5], *anona*[5], *zapote*[5], *capulines*[5],*maguey* juice and *pulque* [3], pine nuts, other seeds and nuts, sweet potato, *chayote* [4], *epazote* [9], *hierbasanta*[9], yucca or *mandioca*, plums, *coyol*[5], *guaje*[6], *mamey*[5], papaya, *tejocote*[5], spicy *salsa*, *quelites*[6], coriander, *chipil*[9], *hierba mora*[6], *jabonera*[6], *quintonil*[6], green tomato, *xocoyule*[6].
Kittler [56]	2007	Corn *tortillas*, beans, soups or stews, casseroles, meats (beef, pork, cuts, and organs), stuffed foods (*tacos, flautas, enchiladas, tamales, quesadillas,* burritos), vegetables (potatoes, greens, tomatoes, onions, *chile*peppers), sugar (sugar cane, sweets, dried fruits and vegetables, candied fruits and vegetables, sugared fruit, nut pastes, desserts with eggs or milk,)beverages (coffee, soft drinks, fruits blended with water and sugar, hot chocolate, beer, wine, tequila, *mescal*, whiskey). Other foods include milk and milk products (cow milk, goat milk, evaporated milk, and cheese), meats (goat), poultry (chicken and turkey), fish and seafood (shrimp, red snapper), chicken eggs, legumes (chickpeas), corn (consumed as flour and *pozole*), wheat (breads, roll, *pan dulce*, pasta), rice, fruits (banana, *carambola*, *casimiroa*, *cherimoya*, coconut, custard apple, passion fruit, *guanábana*, guava, lemon, lime, *mamey*, mango, melon, orange, papaya, pineapple, strawberries, sugar cane, sweet sop, prickly pear, *zapote*), vegetables (avocado, cactus, green pumpkin, *jícama*[7], lettuce, peas, plantains, *chayote* [5], squashes, blossoms, sweet potatoes, *tomatillo*, yams, yucca), seasonings (anise, *achiote*, coriander, cinnamon, cocoa, cumin, *epazote*, garlic, *hoja santa*, mace, vanilla), nuts and seeds (pine nuts, pumpkin seeds and sesame seeds), beverages (*atole*, *pulque),* fats and oils (butter and lard), sweeteners (raw brown cane sugar).
Llamas [57]	1935	Basic foods are maize (consumed as *tortillas*, *atolli*, and *tamales*), beans, *chile*, and pulque. Ducks, *cauauhtli*[16], heron, *aztatl*[16], coots, *tzitzicuilotl*[16],white fish, *xohuili*[11], *xalmichi*[11], *pescadillos de arena*[11], *cuitlapetlate*[11], *michcahuon*[11], *axolote*[10],*cueyatl* frogs, *acacueyatl* frogs, *acocili*[10], *ahuautle*[1], *tecuitlatl* algae, *axayácatl*flyes, roots from aquatic plants, flyes, fish, *atetepiz*[1], *atopinan*[1], larvae, *axayácatl*eggs (*ahuautle)*, chia seeds, cacao, *maguey* wine, *maguey* honey, *maguey* sugar, *maguey* vinegar, herbs, ants, *tequizquitl*[10], vanilla, bee honey, *veinacaztli*[9], mushrooms, venison, rabbit, hare, quail and other poultry, tomato, bread, legumes, *juiles*[11].
Long-Solís [58]	2005	Basic foods are corn (consumed as *tortillas, and tamales, atole* and other corn beverages, *huitlacoche*), beans, and squash (including seeds and blossoms). Complemented with *chile* peppers, amaranth (seeds, flowers, stems, and leaves), avocados, cactus paddles, *chayote*[4], chocolate, *jícama*[5], plantains, tomatoes and *tomatillos*[4], tropical fruits (pineapple, papaya, guava, *guanábana,* soursop, custard apple or *cherimoya*, *zapote*, prickly pear, and *pitahaya*), turkeys, citrus fruits (oranges, grapefruits, tangerines, lemons and limes), other fruits

		(apple, pear, cherries, mangoes, grapes, strawberries, plums, peaches, bananas, melons, watermelons), beef, regional cheeses, fresh cream, sheep, goat, pigs, lard, chicken, rice, spices (black pepper, cinnamon, cumin seeds, sesame seeds, aniseed, oregano, cloves, nutmeg, *achiote*, garlic, onions, parsley), sugar (sweets and candy, *chiringuito*[3], soft drinks, and small quantities of desserts), and wheat (bread, *pan dulce* or 'sweet bread' and pasta), butter, coriander, oregano.
López Alonso [59]	1974	Basic food is maize consumed on the cob, as *pinole*, *tortillas*, *tamales*, *atole* and *pozole*. Prickly pear, acorns, *mezquites*[6], roots, herbs, venison, bear, rabbit and poultry, beans, *chile*, squash, vegetables (*chayote,* potato, sweet potato, *mezquites*, *nopales)*, herbs (*quelites*, *quintoniles*, *malva*, *huauzontle*, mushrooms), seasonings (tomato, squash seeds, *xonácatl*onion, pepper, vanilla), fruits (pineapple, *mamey*, *chirimoya*, *guanábana*, *zapotes*, prickly pear, guava, *tejocote*, *capulín*, *nanche*, *hobo*, *pitahaya*, peanuts, and *jícama*), beverages (from maize, chia seeds, *bledos*[14] and cacao), blossoms (*teonacaztli, tecomaxochitl),* honey (from maize, maguey, and bees), alcoholic beverages (from maize and chia seeds; *pulque*), turkey, dogs, rabbit, moles, skunks, *tlacuaches*[2], venison, poultry (quail, ducks, crane, rail, *atotollin*, *huilotas*), fish *(michin, tlacamichin), jumiles*[1], shrimp, crab, turtles, frogs*, acociles*[11], insects (*aneneztli, axaxayácatl, amoyotl, ocuilistac,* and *ahuauhtli),* reptiles (iguana, snakes, lizards), ants, locust, grasshoppers, worms, eggs of birds and turtles, ant honey, wasp honey, salt, and lime.
Márquez Morfín [60]	1991	Basic foods are maize, beans, *aji* (*chile*), fish and seafood. Rabbits, venison, birds, fish, gastropods, oysters, eggs, armadillos, *coati*[2], iguana, turtles, salt, lobster, dog, *chachalaca*[16], turkey, pigfish, catfish, mollusks, trout, *lisa*mullet, sea bass, sole, saw fish, *caballos*[11], *mojarra*[11], octopus, ray, fruits, roots (yucca), honey, deer, partridges, pheasant, *jícama*[7], *macal*[14], cassava, *chaya*[4], tomato, *chayote*[5], *zapote*[7], *ramón*[7], *mamey*[7], *anona*[7], *guanábana*[7], *nance*[7], ducks, pork, chicken, banana, citrus fruits, manatee, other legumes.
Méndez Y Mercado [61]	1993	Basic foods are maize, beans, squash, and *chile*. Complemented with chia seeds, *huatli*[14], avocado, *mamey*[5], *texalcapotl*[5], *xocotl*[5], *zapote*[5], cacao, *quelites*[6], purslane, *mastuerzo*[6], pumpkin seeds, prickly pear, *nopal*[4], animal foods (lizard, mice, rabbits, hares), wheat, fig, pears, apple.
Ojeda-Granados [62]	2017	Essential foods are maize, beans and squash. Enriched with chili plants, leafy greens (*quelites*), tomatoes, amaranth, chia seeds, algae, insects, and other vegetables.
Ortiz de Montellano [63]	1990	Basic foods are corn, amaranth, beans, squash. Supplemented with *chile* and tomato. Other foods are *tecuitlatl* algae, *mesquite*[6]pods and seeds, maguey leaves, *pulque*[3]. Animal protein sources: water fowl, armadillos, pocket gophers, weasels, rattlesnakes, mice, iguanas, turkeys, dogs, fish, frogs, salamanders, fish eggs, corixid water beetles (*axayácatl*) and their eggs, dragonfly larvae, grasshoppers, ants, worms, *charales*[11], *jumiles*[1], *escamoles*[1], *ahuauhtle*[1], maguey worms, *chilocuiles*[1], *chicatana* ants, stink bugs, worms, sugar water of honey ants.
Quevedo [64]	2004	Basic foods are *maguey* and *aguamiel*, *nopal*[4], *chile*, maize (consumed as *tamales*, popcorn), beans, squash, and amaranth. Complemented with greens (purslane, *quelites, quintoniles,* coriander, *pipizca, pápalo),*mushrooms, *cuitlacochi*[17],avocado, peanuts, cacao drinks, tomato, potato, *jícama*[5], *tecuitlatl* algae, fruits (papaya, *anona* or *chirimoya*, guava, *mamey, zapote, chizozapote*, plums,*tejocote, capulín*, prickly pear, *chayote, chilacayote*, *guanábana, parota, nanche*), wine, butterfly larvae, *chicatana*ant larvae, *maguey* worms, grasshoppers, green *Mixteca*worms, *jumiles*[1], sweet potato, yucca, *cuajilote*[5], coconut, *epazote* [9], *guaje*[6]. Mammals (venison, wild pig, rabbit, hare, monkey,

		tepezcuintle, coyote, *tlacuache*, skunk, otter, weasel, tapir, marten, armadillo, bear, wild boar), poultry (turkey, pheasant, pigeon, quail, duck, goose, *chichicuilote*, partridges, hen), frogs, snakes, turtles, iguana, alligator, sea and freshwater fish (*acociles*, *charales*, *atepocates*, shrimp, white fish, oyster, *jaibas)*, dogs. Honey (from *maguey*, maize, bee, ant), carp, catfish, peccary, manatee, *tequesquite*[9]. Condiments: *acuyo*, *achiote*, squash seeds, *huamuchil*, sesame seeds, vanilla, hibiscus, tamarind, chia seeds.
Quiñonez Tapia [65]	2019	Basic foods are maize (consumed as *tamales*, *atole*, *pozole*, *gorditas*, *tortillas*, ball doughs, *pinole*, popcorn,*totopos*), beans, and squash. *Tejuino*[7], *peyote* [4], squash seed, purslane, *nopales*[4], *piloncillo* [18], *moronga* (cold cut), broths (with venison, veal, hen, fish or iguana), *zapote*[5], *pitaya*[5], *quelites*[6], mushrooms, *guaje*[6], amaranth, *guamúchiles*[5], squirrel, armadillo, turkey, poultry, eggs, *chile*, tomato, and onion.
Quiroz [66]	2005	Fruits and vegetables, meats,grains,*pulque*[3], maize (consumed as *atole*, *tortillas*, *tamales*), wheat flour, mutton, hen, chicken, beef, eggs, salt, bell peppers, pig lard, sugar, cheese, legumes, seeds, chocolate drinks, pork, turkey, *chiringuito* or *aguardiente*, cold cuts *(longaniza, moronga, queso de puerco, escabeches de pata*, ham, *chorizo)*, bread and pastas, *chile*, shrimp, fish, broad beans, beans, chickpeas, lemons, oranges, onion, caper, olives, leafy greens, tomato, lettuce, pineapple, pear, apple, sweet potato, banana, avocado, garlic, beetroot, carrots, artichokes, radishes, cauliflower, jam or fruit sweets,*zapote*[5]*, mamey*[5], *tejocote*[5], pomegranate, coconut, quince, apricot, fig, cherry, plum, bee honey, maguey honey, maize honey, condiments and spices(raisins, pine nuts, cinnamon, sesame seeds, almonds, pepper, clove, vinegar, peppermint, saffron, oregano, nutmeg, parsley, coriander).
Román [67]	2013	Corn, beans, cactus plants, greens (*quelites*), pumpkin seeds, chia, amaranth, c*hile*, avocado, squash, fruits, turkey, deer, dogs, iguana, frogs, armadillo, insects, sea and lake animals, *quelites*[6], prickly pear, *biznaga*[4]*, mesquite*[6], green and red tomato.
Romero Gwynn [68]	1994	Corn, beans, squash, tomatoes, chocolate, chilies, sweet potato, greens (*quelites*), fruits (*mamey, chirimoya, zapotes*, *guanábana*, prickly pear, *pitaya, jícama* and papaya), animals (deer, turkeys, birds, monkeys, tortoise, frogs, fish, insects), wheat, pastas, rice, barley, olives, citrus fruits, almonds, beef, goat meat, chicken, dairy products, onions, garlic, cinnamon, sugar cane, hogs, bread, pastries, pastas.
Santiago-Torres [69]	2015	High intakes of corn tortillas, beans, soups, Mexican dishes (e.g. *tamales*), vegetables, whole fruits, rice, full-fat milk, full-fat Mexican cheeses. Low intakes of oil, solid fats, added sugars, processed meats and refined grains.
Santiago-Torres [70]	2016	Corn based dishes, chilies, garlic, onions, herbs, beans, squash, citrus fruits, rice, meats, lard, corn tortillas, traditional Mexican soups (e.g. *menudo* and *pozole*), traditional Mexican mixed dishes (e.g. *tamales*), vegetables (i.e. *nopales* and *jícama*), animal fats, full-fat milk, and *aguas frescas*(fruits and flowers blended with sugar and water).
Santley [71]	1979	Basic foods are maize (consumed as *tortillas*), amaranth, beans, squashes, chili peppers, and prickly pear. Complemented with chia[12], tomato, *maguey*[4], *nopal*[4], chenopods, purslane, *chayote*[4], fruits (avocado, *capulín, tejocote*, white *zapote*), deer, rabbit, turkey, dog, waterfowl, rodents, reptiles, fish, foxtail grass (*Setaria)*, wild rice, *tecuitlatl* algae, lime.
Shamosh [72]	2014	Basic foods are maize (*tamales*, *tortillas*, *atole*, popcorn, *pinole*, *pozol*), beans, ayocote, squash, *chile*, tomato (green, red, *miltomate*, *jaltomate*), native onion, avocado, *nopal* [4] and prickly pear, amaranth[14], sauces, fermented beverages (from maize, chia seeds, amaranth [14] and cacao; *pulque*, *tesgüino*, *tejuino*, *tuba*, *colonche*), cacao drinks. Seeds and nuts: pumpkin seeds, chia seeds, peanuts, pine nuts,

		sunflower seeds, cotton seeds, mezquite seeds, sesame seeds. Roots (*jícama*, *chilacayote*, *chayote*, potato, sweet potato, *guacamote*, yucca, *tule*, *malanga*, *ayatito*). Vegetables, husks and leaves (*huauzontle*, green beans, *guaje*, *mezquite*, *guamuchil*, *hueynacaxtle*, *cuauhpinole*, *jinicuil*, *cuajilote*, *cuapinole*, *cuachacote*, *quelites*, *quintoniles*, *papaloquelite*, purslane, *tequelite*, *chaya*, *choco,* lettuce, carrots, nabos, auberigne, *bisnagas*, *chaya)*. Flowerblossoms (pumpkin, maguey, yucca, *colorín*, *flor de mayo*, *cabuches*, *beans*, *garambullo*, *huauzontle*, *golumbos*, *cacouite*, bisnaga, *alaches*, cacomite). Mushrooms (*huitlacoche*, wild fungi). Algae (*Spirulina*, *amomoxtle*, *capulín*). Fruits (*capulín*, *tejocote*, *zapote*, *anona*or*chirimoya*, guavas, *nopal* cactus fruit, pineapple, *mamey*, *guanábana*, plums, *nance*, *hobo*, *pitahaya*, papaya, avocado, *xicozapote*, *níspero*, *caimito*, coconut, strawberries, *garambullo*, *pitaya*, *icaco*, grapes, berries, mangoes, tamarind, citrus fruits, fig, peach, pear, banana, melons, cherry, oranges, lime, lemons, grapegruit,*ramón*). Condiments (Tabasco pepper, *epazote*, *acuyo*or*hoja santa*, *achiote*, nativeonion, *xoconotztli*, *moxtle*, *chipilín*, avocado leaves, *chile*leaves, oregano, *anis de monte*, *apio de río*, coriander, *pericón*, *azafrancillo*, vanilla, *yoloxochitl*, *mecaxuchil*, *rosita*, *acedera*, *arrayán*, *orejuela*, *lengua de vaca*). Minerals (salt, lime, *tequesquite*, water). Animal-source foods: mammals (dogs, turkey, monkeys, rabbit, hare, *tlacuache*, venison, wild boar, skunk, racoon, squirrel, pocket gopher, armadillo, mole, mice, pork beef, goat, lamb; cheese, lard, cream), poultry (ducks, *chichicuilote*, pigeon, parrot, pheasant, turtledove, partridge, quail, chicken; poultry eggs), fish (catfish, *michmolli*, and white fish), reptiles (iguana, turtles, alligator, snakes; reptile eggs), frogs, tadpoles, *ajolotes* (a salamander), shrimp, *acociles* (a crayfish), insects (maguey worms, *escamoles*, grasshoppers, *ahuautli*, water-fly, *jumiles,* ants). Beverages (tea, wine, coffee). Sweets: sweeteners (bee, ant, maguey and cactus fruit honey, sugar cane, *piloncillo*), Mexican sweets and milk-based desserts. Cereals: wheat ('sweet bread'), rice, barley, and rye. Legumes: peas, chickpeas, lentils. Condiments: olive oil, cinnamon, onion, parsley, coriander, clove, cinnamon, mint.
Soustelle [73]	1970	Basic foods are maize (cakes/tamales, pottage, *atolli*), beans, amaranth, seeds, chian or sage, plants, batrachians, insects, cocoa drinks, honey, *octli*or*pulque*[3], pimento (or tomato sauce), water. Rarely consumption of meat: venison, poultry (turkey), dogs, rabbits, hares, wild pigs (peccaries), pheasants, crows, doves, lake fowl, frogs, *axolotls*[10], *atepocatl* tadpoles, fresh-water shrimp(*acociltin*), water flies (*amoyotl*), aquatic larvae (*aneneztli*), white worms (*ocuiliztac*), eggs of *axayácatl* (*ahuauhtli*), *tecuitlatl*[19], iguana, ants, agaveworms, sea-fish, turtles, crabs, oysters, *quelites*[6], pulses, peppers, vanilla.
UNESCO [74]	2010	Corn tortilla and tamales, beans and *chile*. Tomatoes, squashes, avocado, cocoa, vanilla.
Vargas [75]	1984	Basic food is maize (consumed as *tortillas*, *tamales*, and*pozol*). Oysters, mussels, shellfish, turtles, fish, venison, *jocote*[5], *matasano*[5], avocado, *Setaria*[6], *huamuchil*[6], *lisa* mullet, sea bass, sardine, *lenguados*[11], sole, *mojarra*[11], octopus, devilfish, manatee meat and lard, pumpkin, beans, fruits (papaya, *zapote*, guava, *mamey*, *ramón), plants (chaya*), roots(*jícama*, sweet potato, yucca, *malanga*,), bee honey, *balch'e*[3], salt, cacao drinks.
Vargas [76]	1988	Basic food is maize (consumed as *tortillas*, *atole, tamales*, and*pozol*). Rabbits, hares, venison, lizards, gopher, *maguey* leaves, *nopales*[4], prickly pear, *setaria*[6], squash, red tomato, green tomato, peanuts, cacao, amaranth, beans, *chile, quelites*[6], *huauzontle*[6], avocado, *zapote*[5],*capulín* [5], guava, *nanche*[5], *mamey*[5], bee or *maguey* honey, dogs, turkeys, peccary, armadillo, *tlacuache*[2], waterfowl, fish and seafood, turtles, manatee, snakes, frogs, *ajolotes*[10], shrimp, insects and their eggs (like *ahuauhtli*), *tecuitlatl*algae, salt, eggs (from turkey, iguanas,

		poultry, and reptiles), fruits (pineapple, *chirimoya*, avocado, *pitahaya*, plum), beverages (water with chia seeds, cacao drinks, *pulque*), lime.
Vargas [77]	2003	Basic foods are maize (consumed as *esquites*, in soups, salads, popcorn, *pozole,* pinole, *tascalate,* tortillas and its variations, *atole*, *pozol*, *tejuino*, *huitlacoche*, *tamales*), beans, squash, green tomato, red tomato, amaranth, avocado, *huauzontle*[6] and *chiles.Setaria* grass, *pochote* root, *mezquite* (leaves, stems and seeds), *mezcal*[3], fruits (prickly pear, *tejocotes*, *capulines*, plums), animals (rabbit, hares, lizards, venison, insects),*maguey*[4](consumed as juice, *pulque*[3], vinegar, core, leaves, blossoms),*quelites*[6], cacao drinks, *achiote*[9], *chipilín*[9], turkey, fish, herbs, sauces, *chayote*[4], sweet potato, yucca, *hoja santa*[9], pepper, dogs, peccary, armadillos, gopher,*maguey* worms, *escamoles*[1], *jumiles*[1], seafood, batrachian, *tecuitlatl* algae, ducks, poultry eggs, salt, chia seeds, wild pigs, pumpkin seeds, wild boar, other eggs, poultry, honey, *ramón*[5], *chaya*[6], edible herbs (purslane, *papaloquelite, lengua de vaca, chivitos, quintoniles, romeritos*, others).
Velasco Lozano [78]	1995	Main foods are maize (consumed as *tortilla*, *tamal*, *atole*, *pozol*, and *pinole*), and beans. Complemented with amaranth, *huauzontle*[6], squash and squash seeds, chia, native fruits (*anona, chirimoya, zapote*, plums, guava, cacao, prickly pear, jicamas, *tejocote*, *capulines*), *maguey*[4], *biznaga*[4], *tule* roots, blossoms (colorín, *izote*, squash), *quelites*[6], roots (*chayote*, *chinchayote*, sweet potato, yucca, *cacomite*, *flor del tigre* root), seasonings (*achiote*, green tomato, red tomato, avocado, *papaloquelite*, *epazote*, *vanilla*, *acuyo*, *chile*, salt and *tequesquite*), animal-source foods (dog, turkey, venison, *tlacuache*[2], armadillo, rabbit, hare, gopher, mice, *cacomixtle*[2], lizards, weasel, squirrel, snakes), insects (ants and their eggs and larvae, bees and wasps larvae), bee honey, maguey honey, grasshoppers, *maguey* worms, *maize* worms, aquatic flora (*tules*, giant reed, nymph, *spirulina* algae), *acociles*[11], worms, fly, dragonfly larvae, amphibians and reptiles (frogs, tadpoles, *ajolotes*, turtles), freshwater fish (*juiles,* whitefish, *charales*), lake poultry (*ocas, ánades,* ducks, heron, *gacetas, ibis, avetoros, martinetes, coot, cranes, somorujos, zampollines, janacas, tringas, falaropos, numénidos, chichicuilotas*, pelicans*).*
Wentworth [79]	1936	Chilli with meat, boiled pinto beans, black coffee, small portions of white bread.
Wicke [80]	1959	Main foods are maize (consumed as *tortillas*, *atole*, and *pozol*), beans,*chile* and squash. Red tomato, honey, chocolate, vanilla, amaranth, pumpkin seeds, fish. Limited intake of meats (rabbit, venison, wild boar, dog, opossum, weasel, mole, snakes, iguana and their eggs, frogs, toad), maguey worms, ants, grasshoppers, *jumiles*[1], white and dark fish, shrimp, snails, salamanders, algae, oysters, turtle and their eggs, water-fly eggs, eels, tadpoles, poultry (ducks, cranes, turkeys, partridge, quail, pheasant), green tomato, *chayote* [4], wild onion, amaranth seeds, sage, avocado, maguey core, c*uitlacoche*[17], mushrooms, tubers (sweet potato, *casabe,* yucca, *jícama*), fruits (prickly pear, *capulín*, guava, *zapote*, pineapple, *tejocote*, blueberries), *pulque*[3].
**Original studies**		

Anderson [81]	1946	Basic foods are corn (consumed as *tortillas*), beans, and chilipeppers. Complemented with *pulque*[3], small quantities sheep or goat (often just blood is consumed), milk, eggs, poultry, rabbit, onion, garlic, *tomate*[4]and *jitomate*[4], lard, cacti, worms, insects, *malva*[6], *hediondilla*[6], prickly pear, *nopales*[4], flowers (of *maguey*,*garambullo*[4], yucca), purslane, *quelites*[6], *xocoyoll*[6], *nabo*leaves and flowers, *lengua de vaca*[6], *endivia*[6].
Beals [82]	1943	Maize, beef, milk, greens, bananas, oranges, beans, pears, cheese, cabbage, *chile*, sugar, fish.
Burgos-Monzon [83]	2013	High intakes of rice, corn and flour tortillas, beans, peas, lentils, eggs, *salsa*[8], and soda.
Carrera [84]	2007	Foods listed from most contribution to total energy intake to least contribution:*tortillas* and *tacos*, flavoured and sweetened drinks, legumes (beans, soy bean), red meat (beef, pork, veal, game, lamb), eggs, cakes, cookies pies, doughnuts, milk and milk products, non-citrus fruits and non-citrus fruit juices, chips and processed snacks, alcoholic beverages, starchy vegetables (potato, sweet potato, plantain, other root crops), soups, bread and other wheat products, candy, chocolate, jams and jellies, other grain products and rice, citrus fruits and citrus fruit juices, breakfast cereals and ready-to-eat cereals, poultry (chicken and turkey), seafood and fish, cheese, vegetables (onion, chili peppers, condiments), processed meats (sausage, bacon), processed fat (margarine, nondairy creamer, other fats), coffee and tea, dairy desserts, nuts and peanut butter, pizza, oils and salad dressings, pastas.
Crocker Sagastume [85]	2004	Basic foods are corn, beans and squash. Complemented with *jitomate* [4] and *chile*, fungus, *quelites* [6], *nopal* [4], *guajes* [6], lard, oil, eggs, onion, *anacates* [17], prickly pear, amaranth.
Flores [86]	2010	Foods listed from most contribution to total energy intake to least contribution: Maize tortillas and maize-based foods (*tamales, atole*), alcohol, Mexican snacks (*tacos* and other tortilla dishes), soft drinks (juice, soda), and white bread and wheat tortillas, beans and legumes (chickpeas), whole-fat dairy products (milk, cheese, yogurt, cream, soups with milk), sweet bread (cake and sweet bread), cookies (sweet and salty), eggs, rice and pasta, low-fat dairy products (milk), fresh fruit (banana, papaya, aple, pear, melon, watermelon, orange, mandarin, guava, mango, pineapple, grapefruit, strawberries), coffee and tea, red meat (pork, beef), high-fibre and ready-to-eat cereal and bread, fast food, sweets and candies, salty snacks, fresh fruit juices, low-fibre and ready-to-eat cereals, processed meats (sausage, ham), poultry (chicken), fish (tuna, sardine), oily seeds and vegetable oils (avocado, nuts), fresh vegetables (broccoli, cauliflower, onion, chayote, cabbage, green beans, lemon, cactus, cucumber, zucchini, chili, carrots, lettuce, green leaves), saturated fat (butter, margarine, mayonnaise), potatos, industrialised vegetables and soups, seafood (shrimp, oyster, crab, octopus).
García-Chávez [87]	2017	Foods listed from most contribution to total energy intake to least contribution: Tortillas (corn and flour), legumes, eggs, SSBs (natural juices, sports and energy drinks, *atole*, coffee, tea, *aguas frescas*, yakult), and bread and other cereals (oatmeal, whole grain cereals), cereals with sugar (sweet bread, cookies), meat and sausages, soups and broths, meals made of tortilla corn dough (*tacos*, *sopes*, *quesadillas*), industrialized beverages, fruits, snacks made from flour, corn or potato, dairy drinks, rice and pasta, desserts, pastries, and sweets, milk drinks with sugar, juices, cheeses, vegetable based stews,potato and other tubers, breakfast cereals with sugar,

		miscellaneous (sauce, seasonings, dressings, creams, emulsifiers), *tortas* and sandwich, fish and seafood, seeds and oils (peanut, nut, seeds, oils, margarine, avocado), yogurt, fast food, vegetables, drinkable yogurt.
McMurry [88]	1991	Basic foods are beans and corn (consumed as *tortillas* and *pinole*). Fruits, vegetables, chili peppers, coffee, and small amounts of sugar and egg whites.
Mercado [89]	2012	Corn (consumed as *tortillas, chilaquiles,atole*, on the cob), beans, bread (plain or 'sweet bread'), coffee, eggs, breakfast cereals, oatmeal, yogurt, *fideo*[14] soup, rice, meats (mainly chicken; few organs), stews, vegetables, *salsa* [9]and chilies, hot chocolate, cinnamon or mint tea, fruit, potatoes, sweet potatoes, fish, home-made drinks made with fruits or flowers, lemonade, whole milk, desserts, vegetable oil, vegetable shortening and lard.
Moreno-Altamirano [90]	2017	Most of the diet is composed of cereals, sugar and sweeteners, meat, legumes, vegetable oils, fruits and vegetables including *chile*, milk and milk products excluding butter.
Murtaugh [91]	2008	High intakes of Mexican cheeses, soups, meat dishes, legumes, tomato-based sauces.
Ravussin [92]	1994	Corn *tortillas*, beans, coffee, sugar, flour tortillas, eggs, potatoes, milk, rice, sodas, pasta soups, green peppers, tomato, cabbage, squash, apples, peaches, oranges, meat, tequila, onion, avocados, bananas, mangoes, chicken and beer.
Rendón [93]	1947	Basic foods are maize (consumed as *tortillas*, *tamales*, *atole*, *totopos*, on the cob, *esquites*, cane), *charales*[11], *quelites*[4]. Complemented with meat (mainly as blood), meat broth, beans, *chile*, dairy products (cheese and *jocoque*), sugar and *piloncillo*[18], salt, lime, eggs, coriander, vegetables (tomato, cabbage, carrots, maguey, *mezquite*, berry leaves, *nopal*, *juakinicuiles*, squash, *chayote* and *chayote* roots, *chilacayote*), condiments (cinnamon, *epazote*, onion, garlic, sauces), wheat, roots, chickpeas, rice, acorns, beverages (water, home-made sodas, *tepache*[3], maguey nectar, *aguardiente*[3], lemon tea, *mezcal*[3], coffee), fruits (tamarind, blackberry, *pitahaya*, prickly pear, lemon, avocado, *chirimoyas*, *zapote*, cherries), animal-foods (fresh fish, squirrels, deer, ducks, poultry, rodents, *jicoteras*[1] and other worms, rabbits, moles,wild boar, hares, fox, skunk, armadillo, lake crabs, *tlacuache*[2], hawk).
Robles-Ordaz [94]	2017	Fish and seafood, low-fat cereals, fruits and vegetables.
Rodríguez-Morán [95]	2009	Mixed root's tortillas, beans, potatoes, prickly pear, green vegetables, bread, milk, fruits, and meat.
Tseng [96]	1997	Beans, corn *tortillas*, chili peppers, whole milk, low-fat milk, and organ meats.
Weitlaner [97]	1952	Maize (consumed as *tortillas*, *tamales*, *atole*, *pinole*, *pozol*), beans, rice, *chile*, squash, squash seeds, wheat, sweet potato, fish, epazote, salt, panela[19], red and green tomato, *quelites*[6], yucca, *jícama*[5], *cebollín*[9], *chayote* [4], cacao drinks, banana, mango, orange, lime, lemon, grapefruit, *chicozapote* [5], guava, avocado, *mamey*[5], *anona*[5], *guanábana*[5], chestnut, tamarind, papaya, *pomarrosa*[5], almond, *capulín*[5], *jobo*[5], coconut, plum,

		acuyo[9], *coyol*[5], *culantro*[9], grapes, *pitaya*[5], mushrooms, beef, pork, blood, lard, cheese, chicken eggs, honey, rabbit, *anteburro*[2], *tigrillo* [2], *tepexcuintle*[2], raccoon, badger, gopher, armadillo, squirrel, wild turkey, *chachalaca* [16], duck, pigeon, parrot, partridge, pigeon and pheasant eggs, *mojarra* [11], bobo mullet, boabino, grey mullet, sea bass, *pepesca*[11], *cuiles*[11], trucha, *jolote*[11], *roncador*[11], crab, snails, shrimp, prawn, grey mullet eggs, turtle eggs, alligator, iguana, *garroba*[20], grasshoppers, water, coffee, wine, honey, bread.
Wyatt [98]	1998	Beans, milk, soda, coffee, corn tortilla, meat, flour tortilla, eggs, potatoes, tomatoes, white bread, pasta soup, Mexican white cheese, rice, beer, oranges, bananas, fish, sugar, and lettuce.

* Foods listed in the order reported by the author(s). Whenever basic foods were mentioned, these were listed first; whenever diverse preparations of the same foods were mentioned, these were grouped. 1: Insects. 2: Mammals. 3: Fermented/alcoholic beverages. 4: Vegetables. 5: Fruits.6: Greens.7: Beverages. 8: Maize preparations. 9: Herbs and condiments. 10: Amphibians. 11: Fish and seafood. 12: Nuts and seeds. 13: Blossoms. 14: Grains. 15: Tubers. 16: Poultry. 17: Fungi. 18: Sweeteners. 19: Algae. 20: Reptiles.

Table S6. Amounts of foods consumed in the traditional Mexican diet according to different authors

a. Amounts reported as percentages of total energy intake

<table>
<tr><th></th><th>Carreraet al. [84]</th><th>Flores et al. [86]</th><th>García-Chávez et al. [87]</th><th>Moreno-Altamirano et al. [90]</th></tr>
<tr><td>Maize *</td><td>35</td><td>47.4</td><td>32.3</td><td rowspan="3">56.1</td></tr>
<tr><td>Bread and wheat products</td><td>1.9</td><td>4.1</td><td>5.1</td></tr>
<tr><td>Other grain/tuber products†</td><td>4.0</td><td>3.2</td><td>2.5</td></tr>
<tr><td>Breakfast-cereals, ready-to-eat cereals</td><td>1.6</td><td>3.0</td><td>0.6</td><td>-</td></tr>
<tr><td>Legumes</td><td>5.6</td><td>3.9</td><td>10.9</td><td>5.8</td></tr>
<tr><td>Vegetables</td><td>1.0</td><td>1.0 [1]</td><td>1.0 [1]</td><td rowspan="2">3.9</td></tr>
<tr><td>Fruits</td><td>5.0</td><td>4.0 [2]</td><td>2.7</td></tr>
<tr><td>Oils, nuts, and fats</td><td>1.7</td><td>1.8</td><td>0.4</td><td>6.9</td></tr>
<tr><td>Alcohol</td><td>2.2</td><td>8.7</td><td>-</td><td>-</td></tr>
<tr><td>Coffee/tea or industrialised beverages</td><td>0.7</td><td>2.2</td><td>3.9</td><td>-</td></tr>
<tr><td>SSBs</td><td>10.8</td><td>4.9</td><td>8.9</td><td>-</td></tr>
<tr><td>Fish and seafood</td><td>1.3</td><td>1.1</td><td>0.5</td><td>0.2</td></tr>
<tr><td>Dairy</td><td>5.0</td><td>6.4</td><td>3.3</td><td>3.8</td></tr>
<tr><td>Poultry</td><td>1.4</td><td>1.1</td><td rowspan="2">5.0</td><td rowspan="2">5.9</td></tr>
<tr><td>Red meat and processed meat</td><td>5.9</td><td>3.3</td></tr>
<tr><td>Eggs</td><td>4.5</td><td>2.8</td><td>8.0</td><td>0.6</td></tr>
<tr><td>Cakes, cookies, desserts, pastries</td><td>4.4</td><td>6.3</td><td>8.0</td><td>-</td></tr>
<tr><td>Sweets, candies, chocolate, jams, jellies</td><td>1.9</td><td>1.6</td><td>-</td><td>-</td></tr>
<tr><td>Sugars and sweeteners</td><td>-</td><td>-</td><td>-</td><td>11.7</td></tr>
<tr><td>Fast foods and salty snacks ‡</td><td>2.9</td><td>8.3</td><td>2.8</td><td>-</td></tr>
<tr><td>Miscellaneous meals, soups/broths</td><td>2.3</td><td>-</td><td>4.6</td><td>-</td></tr>
</table>

* Including maize-based foods. †Starchy vegetables, rice, pasta. ‡ Including Mexican snacks. 1. Includes vegetable soups and stews. 2. Includes fruit juices.

b. Amounts reported as weekly frequencies of consumption

<table>
<tr><th></th><th>4-7 days</th><th>1-3 days</th><th colspan="2"><1 day</th></tr>
<tr><td>Ravussin et al. * [82]</td><td>Corn tortillas
Beans
Coffee
Sugar
Flour tortillas
Eggs
Potatoes</td><td>Milk
Rice
Soda
Pasta soups
Green pepper
Tomato
Cabbage</td><td>Squash
Apples
Peaches
Oranges
Meat
Tequila</td><td>Onion
Avocados
Bananas
Mangos
Chicken
Beer</td></tr>
</table>

* Referring to diets from the Pima Indian's in Northern Mexico in 1991.

c. Amounts reported as weekly quantities consumed (grams/week)

	Beals et al. † [92]	Rodríguez-Morán et al. ‡ [95]	Wyatt et al. * [98]
Maize/maize tortilla	4188	810	847
Wheat [1]: flour tortilla, white bread, pasta soup	-	210	987
Rice	-	-	140
Potatoes	-	310	294
Beans	99	2100	1477
Vegetables [1]	227	750	294
Fruits [1]	367	2400	397
Fish	28	-	70
Beer	-	-	245
Coffee	-	-	1092
Soda	-	-	1344
Dairy [1]	283	630	1652
Meat	226	120	469
Eggs	-	-	364
Sugar	28	-	91
Chile	42	-	-

* Referring to diets in Northern Mexico (no date specified). To facilitate comparisons, the original amounts were converted from grams per day to grams per week. †Referring to diets of Tarascan Indians in Central Mexico in 1941-1942. To facilitate comparison, the original amounts were converted from ounces per week to grams per week. ‡Referring to diets of Tepanhuano Indians in Northern Mexico in 1995-1996. 1. Items grouped to facilitate comparisons.

Table S7. Quality assessment of the included articles

First author	Year	Years or period represented	Description of geographical areas covered	Population located in Mexico or of Mexican ancestry	Description of population represented	Methodology described	Description of foods included	Proportions, quantities or frequencies reported
Aguirre-Beltrán	1994	+	−	+	+	+	+	−
Allen	1992	+	+	+	+	?	+	−
Algert	1998	−	+	+	−	?	+	−
Almaguer	2018	+	+	+	−	?	+	−
Anderson	1946	+	+	+	+	+	+	−
Avila-Nava	2017	+	+	+	+	?	+	−
Barros	1999	+	−	+	+	+	+	−
Beals	1943	+	+	+	+	+	+	+
Berdan	2017	+	+	+	+	?	+	−
Bertran Vilà	2010	+	+	+	+	?	+	−
Bertrán	2005	+	+	+	+	?	+	−
Bertran	2006	−	+	+	−	?	+	−
Burgos-Monzon	2013	+	+	+	+	+	+	−
Carrera	2007	+	+	+	+	+	+	+
Casillas	1984	+	+	+	+	+	+	−
Castelló Yturbide	1986	+	+	+	+	+	+	−
Crocker Sagastume	2004	+	+	+	+	+	+	−
Cook	1980	+	+	+	+	+	+	−
Dávalos Hurtado	1994	−	+	+	+	+	+	−
Flores et al.	2010	+	+	+	+	+	+	+
Flores y Escalante	2004	+	+	+	−	?	+	−
Garcia-Chavez	2017	+	+	+	+	+	+	+
Garcia-Uriguen	2012	+	+	+	+	+	+	−
Harris	2004	+	−	+	−	?	+	−
Katz	1990	+	+	+	+	+	+	−
Kittler	2007	+	+	+	−	+	+	−
Llamas	1935	+	+	+	+	+	+	−
Long-Solis	2005	+	+	+	−	+	+	−
Lopez Alonso	1974	+	+	+	+	+	+	−
Márquez-Morfín	1991	+	+	+	+	+	+	−
McMurry	1991	−	+	+	+	+	+	−

First author	Year	Years or period represented	Description of geographical areas covered	Population located in Mexico or of Mexican ancestry	Description of population represented	Methodology described	Description of foods included	Proportions, quantities or frequencies reported
Mendez y Mercado	1993	+	+	+	+	+	+	−
Mercado	2012	−	−	+	+	+	+	−
Moreno-Altamirano	2017	+	+	+	−	+	+	+
Murtaugh	2008	+	+	+	+	+	+	−
Ojeda-Granados	2017	+	+	+	+	?	+	−
Ortiz de Montellano	1990	+	+	+	+	+	+	−
Quevedo	2004	+	+	+	+	?	+	−
Quiñones Tapia	2019	−	+	+	+	?	+	−
Quiroz	2004	+	+	+	−	+	+	−
Ravussin	1994	+	+	+	+	+	+	+
Rendon	1947	+	+	+	+	+	+	−
Robles-Ordaz	2017	+	+	+	+	+	+	−
Rodríguez-Morán	2009	+	+	+	+	+	+	+
Román	2013	+	+	+	+	?	+	−
Romero-Gwynn	1994	+	+	+	+	+	+	−
Santiago-Torres	2015	−	−	+	+	+	+	−
Santiago-Torres	2016	+	−	+	−	+	+	−
Santley	1979	+	+	+	+	+	+	−
Shamosh	2014	+	+	+	−	+	+	−
Soustelle	1970	+	+	+	+	+	+	−
Tseng	1997	+	+	+	+	+	+	−
UNESCO	2010	−	+	+	−	−	+	−
Vargas	1984	+	+	+	+	+	+	−
Vargas	1988	+	+	+	+	+	+	−
Vargas	2003	+	+	+	+	+	+	−
Velasco	1995	+	+	+	+	+	+	−
Weitlaner	1952	+	+	+	+	+	+	−
Wentworth	1936	−	−	+	+	−	+	−
Wicke	1959	+	+	+	+	?	+	−
Wyatt	1998	−	+	+	+	+	+	+

a. Case-control study

	Adequate case definition	Representativeness of cases	Selection of controls	Definition of controls	Comparability of cases and controls	Ascertainment of exposure	Cases and controls: same ascertainment method	Cases and controls: same non-response rate
Murtaugh et al., 2008	+	+	+	?	+	−	+	−

b. Cross-sectional studies

	Representativeness of sample	Sample size	Non-respondents	Ascertainment of the exposure	Controlling for confounding factors	Assessment of outcome	Statistical test
Carrera et al., 2007	+	+	−	+	?	+	+
Flores et al., 2010	+	+	−	+	?	+	+
Robles-Ordaz et al., 2017	+	−	−	?	?	+	+

c. Cohort study

	Representativeness exposed cohort	Selection of non-exposed cohort	Ascertainment of exposure	Outcome of interest was not present at start	Controlling for confounding factors	Assessment of outcome	Follow-up long enough	Adequacy of follow-up
Santiago-Torres et al., 2015	+	+	?	+	+	+	+	+

d. Randomisedcross-over feeding trial

	Random sequence generation	Allocation concealment	Blinding participants and personel	Blinding of outcome assessment	Incomplete outcome data	Selective reporting	Other bias
Santiago-Torres et al., 2016	?	?	+	+	+	+	+

Figure S1: Risk of bias assessment of the included studies examining the association of the traditional Mexican diet with health outcomes

Table S8. Reporting quality assessment of the included articles, based on the STROBE 2007 checklist

First author [ref.]	Items reported, total reported (%)	Items partially reported, total partially reported (%)	Items not reported, total not reported (%)	Not applicable items, total (%)
Carrera [82]	2, 3, 4, 6, 7, 9, 12a, 12b, 14a, 15, 17, 18, 20, 21 **(41%)**	1b, 5, 8, 11, 12c, 13a, 13b, 16a, 19 **(26%)**	1a, 10, 12d, 12e, 13c, 14b, 22 **(21%)**	6b, 14c, 16b, 16c **(12%)**
Flores [84]	2, 3, 4, 5, 6a, 7, 8, 11, 12a, 12c, 12d, 14a, 15, 16b, 18, 20, 22 **(50%)**	1b, 9, 10, 13a, 13b, 16a, 19, 21 **(23%)**	1a, 12e, 13c, 14b, 16c, 17 **(18%)**	6b, 12b, 14c **(9%)**
Murtaugh [89]	1b, 2, 3, 4, 5, 6a, 7, 8, 11, 12a, 12b, 12d, 14a, 15, 16b, 17, 18, 20, 22 **(56%)**	6b, 9, 16a, 19 **(12%)**	1a, 10, 12c, 12e, 13a, 13b, 13c, 14b, 16c, 21 **(29%)**	14c **(3%)**
Robles-Ordaz [92]	1b, 2, 3, 4, 5, 6a, 7, 11, 12a, 13a, 14a, 15, 16b, 18, 20, 22 **(47%)**	8, 9, 10, 13b, 16a, 19 **(18%)**	1a, 12c, 12e, 13c, 14b, 16c, 17, 21 **(23%)**	6b, 12b, 12d, 14c **(12%)**
Santiago-Torres [67]	1b, 2, 3, 4, 5, 6a, 7, 8, 11, 12a, 12b, 12e, 14a, 15, 16b, 17, 18, 20 **(53%)**	9, 12c, 13a, 13b, 14c, 16a, 19, 21, 22 **(26%)**	1a, 10, 12d, 13c, 14b **(15%)**	6b, 16c **(6%)**

Table S9. Reporting quality assessment of the included articles, based on the CONSORT 2010 checklist

First author [ref.]	Items reported, total reported (%)	Items partially reported, total partially reported (%)	Items not reported, total not reported (%)	Not applicable items, total (%)
Santiago-Torres [68]	1a, 1b, 2a, 2b, 4a, 4b, 5, 6a, 7a, 12a, 12b, 13a, 13b, 14a, 15, 16, 18, 21, 22, 23, 24 **(57%)**	3a, 11a, 17a, 20, 25 **(13%)**	3b, 6b, 8a, 8b, 9, 10, 19 **(19%)**	7b, 11b, 14b, 17b **(11%)**

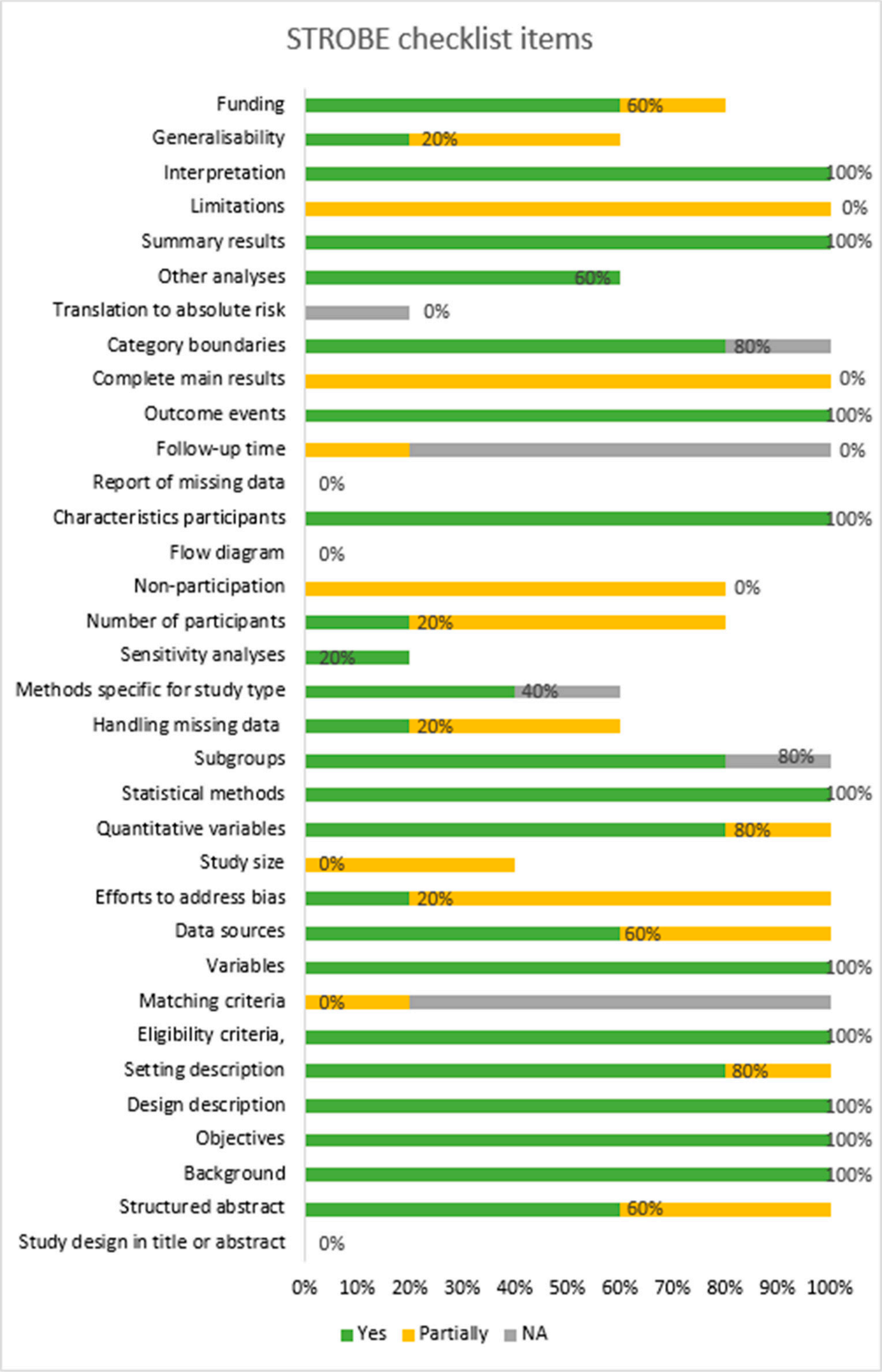

Figure S2: Proportion of reported items in observational studies, based on the STROBE 2007 checklist.

Table S10. Food groups mentioned in the different subgroups evaluated

	Grains and tubers	Maize products	Legumes	Vegetables	Fruits	Oils and fats	Nuts and seeds	Beverages	Fish and seafood	Meats	Eggs	Dairy	Insects	Reptiles	Sweets and sweeteners	Herbs and condiments
All studies	75%	75%	75%	75%	75%	50%	50%	50%	50%	75%	>50%	>50%	>50%	>50%	50%	75%
Reviews	75%	75%	75%	75%	75%	50%	50%	75%	50%	75%	>50%	>50%	50%	50%	50%	75%
Original	75%	50%	75%	75%	75%	50%	>50%	50%	50%	75%	50%	75%	>50%	>50%	50%	50%
North	75%	50%	75%	75%	75%	>50%	>50%	50%	50%	50%	75%	>50%	>50%	>50%	50%	75%
Centre	75%	75%	75%	75%	50%	50%	50%	75%	75%	75%	>50%	>50%	50%	50%	50%	75%
South	75%	50%	75%	75%	75%	75%	75%	50%	50%	75%	>50%	>50%	75%	50%	50%	75%
All regions	75%	75%	75%	75%	75%	75%	50%	75%	50%	75%	50%	50%	50%	>50%	75%	75%

Table S11. Individual foods mentioned in the different subgroups evaluated

	Grains and tubers							Maize products						Legumes	Oils and fats				Nuts and seeds				Fish and seafood	
	Maize	Amaranth	Rice	Wheat	Potato	Sweet potato	Yucca	Tortillas	Tamales	Drinks	Soups	Pinole	Other	Beans	Avocado	Lard	Cream	Veg oil	Peanuts	Pumpkin seeds	Chia seeds	Sesame seeds	Catfish	Shrimp
**All studies**	Yes	Yes	Yes	Yes	Yes	Yes	No	Yes	Yes	Yes	No	No	No	Yes	Yes	No	No	No	No	Yes	Yes	No	No	No
**Reviews**	Yes	Yes	No	Yes	Yes	Yes	Yes	Yes	Yes	Yes	No	No	No	Yes	Yes	No	No	No	Yes	Yes	Yes	No	No	Yes
**Original**	Yes	No	Yes	Yes	Yes	No	No	Yes	No	Yes	No	No	No	Yes	Yes	No	No	Yes	N/A	N/A	N/A	N/A	No	No
**North**	Yes	Yes	Yes	Yes	Yes	No	No	Yes	No	No	No	Yes	No	Yes	N/A	N/A	N/A	N/A	N/A	N/A	N/A	N/A	N/A	N/A
**Centre**	Yes	Yes	No	No	No	Yes	No	Yes	Yes	Yes	No	No	No	Yes	Yes	No	No	No	Yes	Yes	Yes	No	No	Yes
**South**	Yes	Yes	No	No	No	Yes	Yes	Yes	Yes	Yes	No	Yes	No	Yes	Yes	Yes	No	No	No	Yes	Yes	No	Yes	Yes
**All regions**	Yes	Yes	Yes	Yes	Yes	Yes	Yes	Yes	Yes	Yes	Yes	No	Yes	Yes	Yes	No	Yes	Yes	Yes	Yes	Yes	Yes	No	Yes

	Vegetables																		
	Squash	Chayote	Nopales	Tomato	Tomatillo	Guaje	Quelites	Maguey	Mezquite	Mushrooms	Algae	Huauzontle	Carrot	Lettuce	Purslane	Papaloquelite	Quintoniles	Huitlacoche	Squash blossoms
**All studies**	Yes	Yes	Yes	Yes	Yes	No	Yes	No	No	No	No	No	No	No	No	No	No	No	No
**Reviews**	Yes	Yes	Yes	Yes	Yes	No	Yes	Yes	No	Yes	Yes	No	No	No	No	No	No	No	No
**Original**	Yes	No	No	Yes	No	No	No	No	No	No	No	No	No	No	No	No	No	No	No
**North**	Yes	No	No	Yes	No	Yes	Yes	No	No	Yes	No	No	No	No	No	No	No	No	No
**Centre**	Yes	Yes	Yes	Yes	Yes	No	Yes	Yes	Yes	Yes	Yes	Yes	No	No	No	No	No	No	No
**South**	Yes	Yes	Yes	Yes	Yes	Yes	Yes	No	Yes	Yes	Yes	Yes	No	No	Yes	Yes	Yes	No	No
**All regions**	Yes	Yes	Yes	Yes	Yes	No	Yes	No	No	Yes	No	No	Yes	Yes	Yes	No	Yes	Yes	Yes

	Fruits																						
	Anona	Apple	Banana	Berries	Capulin	Citrus fruits	Guava	Guanabana	Jicama	Mamey	Mango	Melon	Nanche	Papaya	Peach	Pear	Pineapple	Pitahaya	Plums	Ramon	Tejocote	Prickly pear	Zapote
**All studies**	Yes	No	No	No	Yes	Yes	Yes	No	Yes	Yes	No	No	No	No	No	No	No	No	Yes	No	No	Yes	Yes
**Reviews**	Yes	No	No	No	Yes	Yes	Yes	Yes	Yes	Yes	No	No	No	Yes	No	No	Yes	No	Yes	No	Yes	Yes	Yes
**Original**	No	No	Yes	No	No	Yes	No	No	No	No	No	No	No	No	No	No	No	No	No	No	No	No	No
**North**	No	No	Yes	No	No	Yes	No	No	No	No	No	No	No	No	No	No	No	No	No	No	No	Yes	No
**Centre**	Yes	No	No	No	Yes	No	Yes	No	Yes	Yes	No	No	No	No	No	No	No	No	Yes	No	Yes	Yes	Yes
**South**	Yes	No	No	No	Yes	No	Yes	Yes	Yes	Yes	No	No	Yes	Yes	No	No	No	No	Yes	Yes	Yes	Yes	Yes
**All regions**	Yes	Yes	Yes	Yes	Yes	Yes	Yes	Yes	Yes	Yes	Yes	Yes	No	Yes	Yes	Yes	Yes	Yes	Yes	No	Yes	Yes	Yes

	Meats																	Eggs	Dairy		Insects					
	Venison	Pork	Rabbit	Hare	Beef	Lamb	Gopher	Boar	Tlacuache	Chevon	Dogs	Armadillo	Squirrel	Turkey	Chicken	Ducks	Partridges	Chicken	Milk	Cheese	Grasshopers	Larvae	Maguey worms	Other worms	Ahuahutle	Amoyotl
**All studies**	Yes	No	Yes	No	Yes	No	No	No	No	No	Yes	Yes	No	Yes	Yes	Yes	No	N/A	N/A	N/A	No	No	No	No	No	No
**Reviews**	Yes	Yes	Yes	Yes	Yes	No	No	Yes	Yes	No	Yes	Yes	No	Yes	Yes	Yes	No	N/A	N/A	N/A	Yes	Yes	Yes	No	No	No
**Original**	No	No	No	No	No	No	No	No	No	No	No	No	No	No	No	No	No	No	Yes	Yes	No	No	No	No	No	No
**North**	No	No	No	No	No	No	No	No	No	No	No	No	No	No	Yes	No	No	No	Yes	No	No	No	No	No	No	No
**Centre**	Yes	No	Yes	Yes	No	No	Yes	Yes	Yes	No	Yes	Yes	No	Yes	Yes	Yes	No	N/A	N/A	N/A	No	Yes	Yes	Yes	Yes	Yes
**South**	Yes	Yes	Yes	Yes	No	No	Yes	Yes	No	No	Yes	Yes	Yes	Yes	No	Yes	Yes	N/A	N/A	N/A	Yes	Yes	No	No	No	No
**All regions**	Yes	Yes	Yes	No	Yes	Yes	No	No	No	Yes	Yes	No	No	Yes	Yes	No	No	Yes	Yes	Yes	Yes	Yes	No	No	No	No

	Reptiles				Sweets and sweeteners					Herbs and condiments												
	Lizards	Snakes	Turtle	Iguana	Honey	Pan dulce	Sugar	Desserts	Sweets	Acedera	Achiote	Acuyo	Chile	Coriander	Chipilín	Epazote	Garlic	Onion	Parsley	Pepper	Salt	Vanilla
**All studies**	N/A	N/A	N/A	N/A	Yes	No	Yes	No	No	No	No	No	Yes	No	No	No	No	Yes	No	No	Yes	No
**Reviews**	No	Yes	Yes	Yes	Yes	No	No	No	No	No	No	No	Yes	No	No	Yes	No	Yes	No	No	Yes	Yes
**Original**	N/A	N/A	N/A	N/A	No	No	Yes	No	No	No	No	No	Yes	No	No	No	No	Yes	No	No	No	No
**North**	N/A	N/A	N/A	N/A	No	No	Yes	No	No	No	No	No	Yes	No	No	No	No	Yes	No	No	No	No
**Centre**	Yes	Yes	Yes	Yes	Yes	No	No	No	No	No	No	No	Yes	No	No	Yes	No	Yes	No	No	Yes	Yes
**South**	Yes	No	Yes	Yes	Yes	No	No	No	No	Yes	No	Yes	Yes	No	Yes	Yes	No	Yes	No	No	Yes	No
**All regions**	N/A	N/A	N/A	N/A	Yes	Yes	Yes	Yes	Yes	No	Yes	Yes	Yes	Yes	No	Yes	Yes	Yes	Yes	Yes	No	Yes

Supplementary Materials II

Search strategy

CENTRAL and Cochrane Reviews

Date: 9 July 2019

Search strategy used: 'Mexic* and traditional or native or regional or pre-Hispanic or indigenous or Mesoamerica$ or Aztec or Maya and diet or "dietary pattern$" or "food pattern$" or "eating pattern$" or "food habits" or "eating habits" or "dietary habits" or cuisine in **Cochrane Reviews**'

AND

'Mexic* and traditional or native or regional or pre-Hispanic or indigenous or Mesoamerica$ or Aztec or Maya and diet or "dietary pattern$" or "food pattern$" or "eating pattern$" or "food habits" or "eating habits" or "dietary habits" or cuisine in **Trials**'

Total articles retrieved: **143**

Cumulative Index of Nursing and Allied Health Literature (CINAHL) and Anthropology Plus

Date: 9 July 2019

Search strategy used: Mexic* AND (traditional or native or regional or pre-Hispanic or indigenous or Mesoamerica$ or Aztec or Maya) AND (diet or "dietary pattern$" or "food pattern$" or "eating pattern$" or “food habits” or "eating habits" or "dietary habits" or cuisine)

Total articles retrieved: 112

eHRAF World Cultures

Date: 9 July 2019

Search strategy used:

Mexic*

AND

traditional or native or regional or pre-Hispanic or indigenous or Mesoamerica$ or Aztec or Maya

AND

diet or "dietary pattern$" or "food pattern$" or "eating pattern$" or "food habits" or "eating habits" or "dietary habits" or cuisine

Filters

- Culture (by country)

Included: All cultures from Mexico (Aztecs, Huichol, Mam Maya, Maya, Nahua, Tarahumara, Tzeltal, Zapotec, Eastern Apache, O'odham)

Total articles retrieved: 17

Embase <1974 to 2019 Week 27>/ Ovid MEDLINE(R) <1946 to July Week 2 2019> / PsycINFO <1806 to July Week 2 2019>

Date: 9 July 2019

Search strategy used: (Mexic* and (traditional or native or regional or pre-Hispanic or indigenous or Mesoamerica$ or Aztec or Maya) and (diet or dietary pattern$ or food pattern$ or eating pattern$ or food habits or eating habits or dietary habits or cuisine)).af.

Total articles retrieved: 2,977

LILACS

Date: 9 July 2019

Search strategy used: (tw:(Mexic*)) AND (tw:(traditional or native or regional or pre-Hispanic or indigenous or Mesoamerica$ or Aztec or Maya)) AND (tw:(diet or dietary pattern$ or food pattern$ or eating pattern$ or "food habits" or "eating habits" or "dietary habits" or cuisine))

Filters

- Database

Included: LILACS

- Language

Included: English and Spanish

Total articles retrieved: 138

ProQuest Dissertations & Theses Global

Date: 9 July 2019

Search strategy used: ab(Mexic*) AND (traditional or native or regional or pre-Hispanic or indigenous or Mesoamerica$ or Aztec or Maya) AND ab(diet or "dietary pattern$" or "food pattern$" or "eating pattern$" or "food habits" or "eating habits" or "dietary habits" or cuisine)

Filters

- Full-text
- Language

Included: English and Spanish

Total articles retrieved: 331

Redalyc

Date: 9 July 2019

Search strategy used: (Mexic*) AND (traditional or native or pre-Hispanic or regional or indigenous or Mesoamerica$ or Aztec or Maya) AND (diet or "dietary pattern$" or "food pattern$" or "eating pattern$" or "food habits" or "eating habits" or "dietary habits" or cuisine)

Filters

- Subject area

Included: Biology, multidisciplinary (social sciences), anthropology, medicine, sociology, health, history, cultural studies.

Not Included: Agricultural sciences, agrarian studies, veterinary, Earth studies, politics, chemistry, territorial studies, psychology, education, language and literature, multidisciplinary (natural and exact sciences), environmental studies, education, territorial studies, psychology, communication.

- Language

Included: English and Spanish

Total articles retrieved: 274

SciELO

Date: 9 July 2019

Search strategy used: (Mexic*) AND (traditional or native or regional or pre-Hispanic or indigenous or Mesoamerica* or Aztec or Maya) AND (diet or dietary pattern* or food pattern* or eating pattern* or "food habits" or "eating habits" or "dietary habits" or cuisine)

Filters

- SciELO thematic areas

Included: Health sciences, biological sciences, applied social sciences, human sciences, multidisciplinary

Not Included: Agricultural sciences, exact and Earth sciences, literature and arts, engineering.

- Language

Included: English and Spanish.

Total articles retrieved: 329

Web of Science

Date: 9 July 2019

Search strategy used: **TOPIC:** (Mexic*) *AND***TOPIC:** (traditional or native or regional or pre-Hispanic or indigenous or Mesoamerica$ or Aztec or Maya) *AND***TOPIC:** (diet or "dietary pattern$" or "food pattern$" or "eating pattern$" or "food habits" or "eating habits" or "dietary habits" or cuisine)

Filters:

- Web of Sciences Categories

Included: Nutrition dietetics, public environmental occupational health, archaeology, anthropology, food science technology, endocrinology metabolism, multidisciplinary sciences, biology, medicine general internal, cardiac cardiovascular systems, agronomy, integrative complementary medicine, sociology, behavioural sciences, history, nursing, paediatrics, social sciences biomedical, gastroenterology hepatology, oncology, peripheral vascular disease, demography, social sciences interdisciplinary, ethnic studies, folklore, health care sciences services, health policy services, history philosophy of science, humanities multidisciplinary, urology nephrology.

Not included: Ecology, marine freshwater biology, biodiversity conservation, environmental sciences, agriculture dairy animal sciences, zoology, fisheries, agriculture multidisciplinary, entomology, pharmacology pharmacy, geosciences multidisciplinary, plant sciences, biochemistry molecular biology, horticulture, veterinary sciences, evolutionary biology, oceanography, chemistry medicinal, chemistry applied, education educational research, environmental studies, genetics heredity, medicine research experimental, toxicology, agricultural economics policy, chemistry multidisciplinary, physiology, psychology multidisciplinary, biotechnology applied microbiology, business, education scientific disciplines, forestry, geography, green sustainable science technology, limnology, microbiology, ornithology, parasitology, psychiatry, psychology, respiratory system, water resources, biochemical research methods, biophysics, chemistry analytical, chemistry inorganic nuclear, chemistry physical, communication, critical care medicine, engineering chemical, geography physical, geriatrics gerontology, infectious diseases, information science library science, law, literary theory criticism, materials science characterisation testing, mycology, planning development, psychology clinical, psychology developmental, public administration, rehabilitation, religion, sport sciences, thermodynamics, urban studies, women studies, economics, hospitality leisure sport tourism.

- Document type

Excluded: Meeting abstracts.

Total articles retrieved: 286

Hand-searching:

1. *Revista de Salud Pública y Nutrición: None found*
